# Synthesis and Activity Evaluation of Vinpocetine-Derived Indole Alkaloids

**DOI:** 10.3390/molecules29010014

**Published:** 2023-12-19

**Authors:** Zhang-Chao Dong, Yang Shi, Liang-Liang Zheng, You-Ping Tian, Jian Yang, Ying Wei, Ying Zhou, Bo-Wen Pan

**Affiliations:** 1 College of Pharmacy, Guizhou University of Traditional Chinese Medicine, Guiyang 550025, China; dongzhangchao0824@163.com (Z.-C.D.); xiaoyewater@163.com (Y.S.); zx15117508142@163.com (L.-L.Z.); nanshan-tia@163.com (Y.-P.T.); weiying1969@126.com (Y.W.); 2 State Key Laboratory of Natural and Biomimetic Drugs, School of Pharmaceutical Sciences, Peking University, Beijing 100191, China; 3 College of Pharmacy and Nutrition, University of Saskatchewan, Saskatoon, SK S7N 5E5, Canada

**Keywords:** vinpocetine, PDE1A, structural modification, biological evaluation, molecular docking

## Abstract

This study focuses on the synthesis of novel vinpocetine derivatives (**2**–**25**) and their biological evaluation. The chemical structures of the synthesized compounds were fully characterized using techniques such as ^1^H NMR, ^13^C NMR, and HRMS. The inhibitory activity of the synthesized compounds on PDE1A was evaluated, and the results revealed that compounds **3**, **4**, **5**, **12**, **14**, **21**, and **25** exhibited superior inhibitory activity compared to vinpocetine. Compound **4**, with a para-methylphenyl substitution, showed a 5-fold improvement in inhibitory activity with an IC_50_ value of 3.53 ± 0.25 μM. Additionally, compound **25**, with 3-chlorothiazole substitution, displayed an 8-fold increase in inhibitory activity compared to vinpocetine (IC_50_ = 2.08 ± 0.16 μM). Molecular docking studies were conducted to understand the binding models of compounds **4** and **25** within the active site of PDE1A. The molecular docking study revealed additional binding interactions, such as π–π stacking and hydrogen bonding, contributing to the enhanced inhibitory activity and stability of the ligand–protein complexes. Overall, the synthesized vinpocetine derivatives demonstrated promising inhibitory activity on PDE1A, and the molecular docking studies provided insights into their binding modes, supporting further development of these compounds as potential candidates for drug research and development.

## 1. Introduction

Cerebrovascular disease (CVD) refers to pathological alterations in the arteries supplying the brain or the neck arteries that govern blood flow to the brain. This leads to disruptions in intracranial blood circulation and damage to brain tissue. CVD is a prevalent and frequently occurring health concern, posing a significant threat to human well-being [1]. With the development of society and continuous changes in dietary habits, the incidence of cerebrovascular diseases is on the rise. Due to its high prevalence, high disability rate, and high recurrence rate, cerebrovascular disease stands as a major cause of mortality and disability among the elderly. Pathologically, it can be categorized into hemorrhagic and ischemic cerebrovascular diseases [2,3], with ischemic incidents being the most common, accounting for approximately 70–80% [4]. This imposes a substantial burden on families and society [5,6].

**Figure 1:**
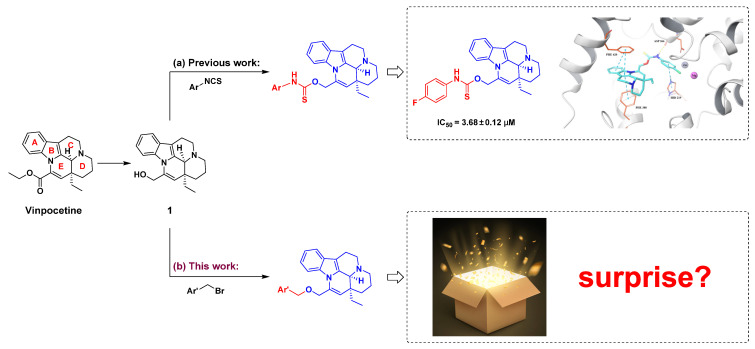
The design route of vinpocetine derivatives.

Vinpocetine (Figure 1), as a semi-synthetic derivative, is derived from the alkaloid vincamine extracted from the lesser periwinkle plant. The basic structure of vinpocetine consists of four hexagonal rings and one pentagonal ring, denoted as A, B, C, D, and E in sequence. Currently, vinpocetine has been on the market for over 30 years for the treatment of ischemic cerebrovascular diseases [7,8].

Vinpocetine has multiple cellular targets, with its initial identified target being phosphodiesterase 1 (PDE1), a member of the phosphodiesterase enzyme superfamily that catalyzes the degradation of cGMP or cAMP [9,10,11,12,13]. PDE1 is categorized into three subtypes based on different gene coding: PDE1A, PDE1B, and PDE1C. As a PDE1A inhibitor, vinpocetine, functioning as a cerebral vasodilator, primarily regulates the levels of cGMP within vascular smooth muscle cells. By elevating cGMP levels [14], it modulates the tone of vascular smooth muscle [15], leading to vasodilation and maintaining or restoring the physiological dilation of cerebral blood vessels. This contributes to improving cerebral blood flow and oxygen utilization, enhancing the supply of glucose and oxygen, as well as ATP generation, consequently further ameliorating cerebral metabolism [16].

Due to these properties, it is widely used in the treatment and prevention of ischemic cerebrovascular diseases. However, some limitations of vinpocetine have gradually become apparent during long-term clinical use. These include the existence of first-pass hepatic metabolism, generating apovincaminic acid and reducing bioavailability. Additionally, its short oral half-life leads to poor patient compliance with multiple dosages [17]. Therefore, there is an urgent need for extensive and in-depth secondary development.

In previous studies, we reduced the ester group of vinpocetine to a hydroxyl group and subsequently subjected it to an addition reaction with isothiocyanate compounds with varying substitutions on the benzene ring. The results indicated that derivatives with para-fluoro substitution on the benzene ring exhibited the most potent inhibitory activity against PDE1A (IC_50_ = 3.68 ± 0.12 μM) [18]. Molecular docking studies revealed that an extended side chain could enhance interactions with the target protein PDE1A, thereby increasing inhibitory activity. Building on this finding, we subjected compound **1** to an addition reaction with halogenated hydrocarbons featuring different substitutions on the benzene ring. Through further structural modifications of vinpocetine, we aim to obtain derivatives that exhibit superior inhibitory activity against PDE1A, potentially leading to new breakthroughs in our research.

## 2. Results and Discussion

### 2.1. Synthesis

**Figure 2:**
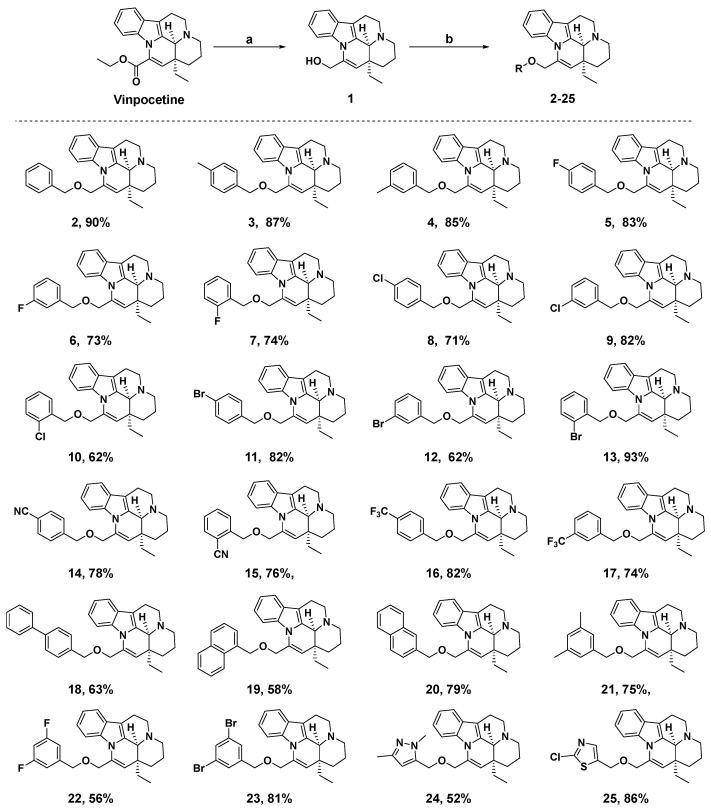
Synthetic route of vinpocetine derivatives **1**–**25**. Reagents and conditions: a. LiAlH_4_, dry THF, rt; b. Halogenated hydrocarbon, NaH (60%), dry THF, rt.

Due to the hydrolysis of the ester moiety in vinpocetine, leading to reduced bioavailability, a design strategy was employed to replace the ester group and extend the side chain, aiming to enhance the interaction between the ligand and the protein. The synthetic route for vinpocetine derivatives **1**–**25** is illustrated in Figure 2. Initially, under the action of lithium aluminum hydride, the ester group at position 14 of vinpocetine was reduced to a hydroxyl group, yielding compound **1**. Subsequently, compound **1** underwent nucleophilic substitution reactions with various halogenated hydrocarbons in the presence of NaH. This synthetic approach resulted in a series of vinpocetine derivatives (**2**–**25**) while preserving the original framework. The chemical structures of all target compounds (**2**–**25**) were thoroughly characterized and confirmed using ^1^H NMR, ^13^C NMR, ^19^F NMR and HRMS. The melting points of the target compounds were also determined. All ^1^H NMR, ^13^C NMR, ^19^F NMR and HRMS spectra were provided in the Supplementary Materials.

### 2.2. Inhibition of PDE1A Activity by Different Compounds 

The synthesized compounds **2**–**25** were evaluated for their inhibitory activity against PDE1A. As shown in Table 1, the results indicate that compounds **3**, **4**, **5**, **12**, **14**, **21**, and **25** exhibit superior inhibitory activity compared to vinpocetine. Structure–activity relationship studies revealed that for derivatives with benzene ring substitutions, the introduction of electron-donating groups to the benzene ring led to enhanced inhibitory activity. Specifically, compound **4**, with para-methyl substitution on the benzene ring, demonstrated a fivefold improvement in inhibitory activity compared to vinpocetine, with an IC_50_ value of 3.53 ± 0.25 μM. Conversely, the introduction of electron-withdrawing groups to the benzene ring resulted in reduced inhibitory activity, with compound **9**, featuring para-chloro substitution, displaying the weakest inhibitory activity with an IC_50_ of 72.14 ± 3.89 μM.

Among heterocyclic substitutions, compound **25**, with a 3-chlorothiazole ring substitution, showed an eightfold increase in inhibitory activity against PDE1A compared to vinpocetine, with an IC_50_ value of 2.08 ± 0.16 μM. This represents a slight improvement in activity compared to the previously synthesized thiocarbamate derivatives. Additionally, compounds with steric hindrance, such as those with biphenyl and naphthalene ring groups, exhibited significantly reduced inhibitory activity. Among them, compound **20** displayed the weakest inhibitory activity with an IC_50_ of 89.64 ± 5.34 μM.

### 2.3. Molecular Docking 

**Figure 3:**
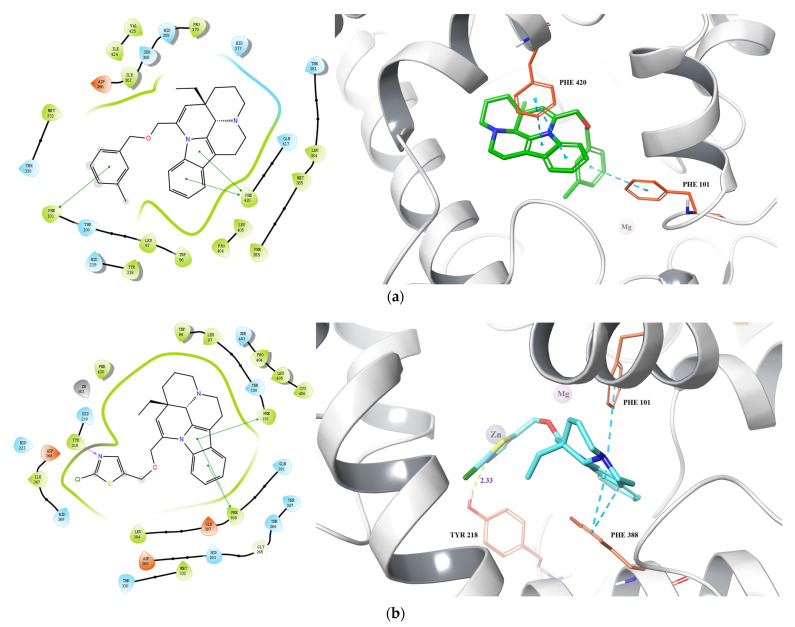
Predicted binding of compounds **4** (**a**) and **25** (**b**) to PDE1A.

To gain a more profound understanding of the binding models of compounds **4** and **25** with the active site of PDE1A and further validate the experimental results, the artificial intelligence tool AlphaFold was employed to obtain the protein model AF-Model of PDE1A. Subsequently, compounds **4** and **25** underwent molecular docking studies with the PDE1A protein model (Table 2 and Figure 3). 

The investigation revealed that the docking scores of compounds **4** and **25** with the PDE1A protein model were −12.027 and −11.326, respectively, while the glide emodel values were −69.613 kcal/mol and −82.876 kcal/mol, respectively. The A and B rings of compound **4** formed two π–π interactions with PHE 420, and the introduced benzene ring stabilized at the binding site through a π–π interaction with PHE 101. For compound **25**, the A and B rings formed three π–π interactions with PHE 388 and PFE 101. Additionally, the introduced thiazole ring formed a hydrogen bond with TYR 218, with a distance of 2.33 Å. Compared to the previously synthesized thiocarbamate derivatives, compounds **4** and **25** similarly increased additional interactions with the target protein through extended side chains, resulting in significantly enhanced inhibitory activity and a more stable ligand–protein complex.

## 3. Materials and Methods

### 3.1. Synthesis of Vinpocetine Derivatives 

Vinpocetine (700 mg, 2 mmol) was dissolved in anhydrous THF (15 mL) and LiAlH_4_ (152 mg, 4 mmol) was added slowly at 0 °C under the protection of argon. The reaction mixture was stirred at room temperature for 10 h until completion, as determined by TLC. Then, it was quenched with wet EtOAc, dried with MgSO_4_, filtered through celite, and washed with abundant EtOAc. The volatiles were removed under reduced pressure to obtain the crude product. The crude product was purified by crystallization to obtain a white solid compound **1** (573 mg, 93%). 

Sodium hydride dispersion (60%) in mineral oil (9 mg, 0.22 mmol) was added in a single portion to a stirred solution (2 mL) of compound **1** (62 mg, 0.2 mmol) in anhydrous tetrahydrofuran at 0 °C. As soon as the hydrogen evolution ceased, appropriately halogenated hydrocarbons (0.3 mmol) were added. The reaction mixture was stirred overnight at room temperature After completion of the reaction monitored by TLC, the mixture was quenched with aqueous ammonium chloride, and then the mixture extracted with ethyl acetate (3 × 10 mL). The organic layer was separated, washed with water and brine, dried over anhydrous sodium sulfate, filtered, and then concentrated under reduced pressure. The residue was purified by silica gel chromatography to give the compounds **2**–**25** (mobile phase: DCM/MeOH).

### 3.2. Compound Characterization

Product **2** ((41S,13aS)-12-((benzyloxy)methyl)-13a-ethyl-2,3,41,5,6,13a-hexahydro-1H-indolo[3,2,1-de]pyrido[3,2,1-ij][1,5]naphthyridine) was a yellow oily substance in 90% yield, mobile phase: DCM/MeOH = 30:1. ^1^H NMR (400 MHz, CDCl_3_) δ 7.65–7.62 (m, 1H), 7.41–7.39 (m, 1H), 7.31–7.28 (m, 2H), 7.25–7.18 (m, 3H), 7.14–7.04 (m, 2H), 5.00 (s, 1H), 4.67 (dd, *J* = 12.7, 0.8 Hz, 1H), 4.62–4.55 (m, 2H), 4.41 (d, *J* = 12.7 Hz, 1H), 4.10 (s, 1H), 3.32–3.27 (m, 1H), 3.22–3.14 (m, 1H), 3.02–2.93 (m, 1H), 2.71–2.58 (m, 2H), 2.48–2.43 (m, 1H), 1.94–1.85 (m, 1H), 1.74–1.62 (m, 2H), 1.41–1.33 (m, 2H), 1.14–1.06 (m, 1H), 0.95 (t, *J* = 7.5 Hz, 3H). ^13^C NMR (100 MHz, CDCl_3_) δ 137.79, 133.98, 131.94, 131.22, 128.97, 128.38, 127.91, 127.70, 122.05, 119.81, 119.36, 118.10, 112.83, 107.96, 71.16, 69.35, 56.10, 51.80, 45.20, 36.85, 30.06, 27.42, 20.66, 16.42, 8.96. HRMS (ESI): Exact mass calcd for C_27_H_30_N_2_O [M+H]^+^: 399.2358, found 399.2428.

Product **3** ((41S,13aS)-13a-ethyl-12-(((4-methylbenzyl)oxy)methyl)-2,3,41,5,6,13a-hexahydro-1H-indolo[3,2,1-de]pyrido[3,2,1-ij][1,5]naphthyridine) was a yellow oily substance in 87% yield, mobile phase: DCM/MeOH = 30:1. ^1^H NMR (400 MHz, CDCl_3_) δ 7.74–7.72 (m, 1H), 7.52–7.50(m, 1H), 7.30–7.28 (m, 1H), 7.24–7.22 (m, 1H), 7.20–7.15 (m, 4H), 5.11 (s, 1H), 4.76 (dd, *J* = 12.8, 0.8 Hz, 1H), 4.67–4.62 (m, 2H), 4.50 (d, *J* = 12.8 Hz, 1H), 4.21 (s, 1H), 3.44–3.39 (m, 1H), 3.33–3.26 (m, 1H), 3.14–3.04 (m, 1H), 2.83–2.73 (m, 2H), 2.60–2.52 (m, 1H), 2.40 (s, 3H), 2.03–1.96 (m, 1H), 1.83–1.78(m, 2H), 1.52–1.45 (m, 2H), 1.25–1.21 (m, 1H), 1.06 (t, *J* = 7.5 Hz, 3H). ^13^C NMR (100 MHz, CDCl_3_) δ 138.15, 137.80, 134.17, 132.22, 128.84, 128.57, 128.38, 125.10, 122.29, 120.00, 119.28, 118.22, 113.06, 107.96, 71.43, 69.47, 56.34, 51.89, 45.27, 37.00, 29.92, 27.51, 21.48, 20.56, 16.50, 8.99. HRMS (ESI): Exact mass calcd for C_28_H_32_N_2_O [M+H]^+^: 413.2515, found 413.2584.

Product **4** ((41S,13aS)-13a-ethyl-12-(((3-methylbenzyl)oxy)methyl)-2,3,41,5,6,13a-hexahydro-1H-indolo[3,2,1-de]pyrido[3,2,1-ij][1,5]naphthyridine) was a yellow oily substance in 85% yield, mobile phase: DCM/MeOH = 25:1. ^1^H NMR (400 MHz, CDCl_3_) δ 7.69–7.67 (m, 1H), 7.46–7.44 (m, 1H), 7.21–7.17 (m, 3H), 7.15–7.13 (m, 1H), 7.09 (s, 2H), 5.06 (s, 1H), 4.74 (d, *J* = 12.7 Hz, 1H), 4.65–4.56 (m, 2H), 4.50 (d, *J* = 12.7 Hz, 1H), 4.21 (s, 1H), 3.46–3.43 (m, 1H), 3.34–3.27 (m, 2H), 3.09–3.00 (m, 2H), 2.82 (s, 1H), 2.66–2.61 (m, 1H), 2.29 (s, 3H), 2.02–1.97 (m, 1H), 1.84–1.81 (m, 2H), 1.47 (s, 1H), 1.26–1.17 (m, 1H), 1.01 (t, *J* = 7.4 Hz, 3H). ^13^C NMR (100 MHz, CDCl_3_) δ 138.17, 137.71, 134.33, 132.47, 128.81, 128.64, 128.60, 128.39, 125.09, 122.66, 120.24, 118.79, 118.32, 113.15, 107.77, 71.58, 69.39, 56.61, 51.95, 45.23, 37.10, 29.50, 27.50, 21.47, 20.09, 16.40, 8.94. HRMS (ESI): Exact mass calcd for C_28_H_32_N_2_O [M+H]^+^: 413.2515, found 413.2579.

Product **5** ((41S,13aS)-13a-ethyl-12-(((4-fluorobenzyl)oxy)methyl)-2,3,41,5,6,13a-hexahydro-1H-indolo[3,2,1-de]pyrido[3,2,1-ij][1,5]naphthyridine) was a yellow oily substance in 83% yield, mobile phase: DCM/MeOH = 28:1. ^1^H NMR (400 MHz, CDCl_3_) δ 7.65–7.63 (m, 1H), 7.44–7.42 (m, 1H), 7.25–7.20 (m, 2H), 7.17–7.07 (m, 2H), 6.98–6.93 (m, 2H), 5.02 (s, 1H), 4.70 (d, *J* = 12.7 Hz, 1H), 4.60–4.52 (m, 2H), 4.42 (d, *J* = 12.7 Hz, 1H), 4.12 (s, 1H), 3.35–3.30 (m, 1H), 3.25–3.17 (m, 1H), 3.05–2.96 (m, 1H), 2.73–2.61 (m, 2H), 2.50–2.44 (m, 1H), 1.97–1.88 (m, 1H), 1.77–1.64 (m, 2H), 1.44–1.36 (m, 2H), 1.16–1.08 (m, 1H), 0.98 (t, *J* = 7.5 Hz, 3H). ^13^C NMR (100 MHz, CDCl_3_) δ 162.38 (d, *J* = 245.7 Hz), 133.99, 133.65, 133.62, 131.90, 131.30, 129.63 (d, *J* = 8.1 Hz), 122.07, 119.88, 119.49, 118.18, 115.25 (d, *J* = 21.4 Hz), 112.78, 108.09, 70.47, 69.51, 56.12, 51.82, 45.23, 36.89, 30.10, 27.45. ^19^F NMR (377 MHz, CDCl_3_) δ -114.70. HRMS (ESI): Exact mass calcd for C_27_H_29_FN_2_O [M+H]^+^: 417.2264 found 417.2328.

Product **6** ((41S,13aS)-13a-ethyl-12-(((3-fluorobenzyl)oxy)methyl)-2,3,41,5,6,13a-hexahydro-1H-indolo[3,2,1-de]pyrido[3,2,1-ij][1,5]naphthyridine) was a yellow oily substance in 73% yield, mobile phase: DCM/MeOH = 30:1. ^1^H NMR (400 MHz, CDCl_3_) δ 7.69–7.67 (m, 1H), 7.47–7.45 (m, 1H), 7.28–7.24 (m, 1H), 7.21–7.17 (m, 1H), 7.14–7.11 (m, 1H), 7.07–7.05 (m, 1H), 7.03–7.00 (m, 1H), 6.98–6.93 (m, 1H), 5.05 (s, 1H), 4.76 (d, *J* = 12.8 Hz, 1H), 4.66–4.58 (m, 2H), 4.47 (d, *J* = 12.8 Hz, 1H), 4.18 (s, 1H), 3.40–3.35 (m, 1H), 3.30–3.22 (m, 1H), 3.07–3.01 (m, 1H), 2.76–2.71 (m, 2H), 2.58–2.53 (m, 1H), 1.99–1.94 (m, 1H), 1.79–1.74 (m,2H), 1.45–1.41 (m, 1H), 1.22–1.13 (m, 1H), 1.00 (t, *J* = 7.5 Hz, 3H). ^13^C NMR (100 MHz, CDCl_3_): δ 163.00 (d, *J* = 246.3 Hz), 140.59 (d, *J* = 7.2 Hz), 134.13, 131.96, 130.77, 130.02, 129.94, 128.96, 123.23 (d, *J* = 2.9 Hz), 122.37, 120.10, 119.47, 118.33, 114.64 (d, *J* = 21.2 Hz), 112.86, 108.11, 70.53, 69.68, 56.27, 51.88, 45.26, 37.04, 29.96, 27.49, 20.53, 16.46, 8.99; ^19^F NMR (376 MHz, CDCl_3_): δ -113.11. HRMS (ESI): Exact mass calcd for C_27_H_29_FN_2_O [M+H]^+^: 417.2264 found 417.2344.

Product **7** ((41S,13aS)-13a-ethyl-12-(((2-fluorobenzyl)oxy)methyl)-2,3,41,5,6,13a-hexahydro-1H-indolo[3,2,1-de]pyrido[3,2,1-ij][1,5]naphthyridine) was a yellow oily substance in 74% yield, mobile phase: DCM/MeOH = 25:1. ^1^H NMR (400 MHz, CDCl_3_) δ 7.66–7.64 (m, 1H), 7.43–7.41 (m, 1H), 7.36–7.32 (m, 1H), 7.24–7.19 (m, 1H), 7.16–7.12 (m, 1H), 7.11–6.98 (m, 3H), 5.07 (s, 1H), 4.72–4.65 (m, 3H), 4.46 (d, *J* = 12.7 Hz, 1H), 4.12 (s, 1H), 3.34–3.30 (m, 1H), 3.24–3.16 (m, 1H), 3.04–2.95 (m, 1H), 2.73–2.67 (m, 1H), 2.63–2.61 (m, 1H), 2.50–2.44 (m, 1H), 1.95–1.90 (m, 1H), 1.76–1.68 (m, 2H), 1.44–1.35 (m, 2H), 1.16–1.08 (m, 1H), 0.98 (t, *J* = 7.5 Hz, 3H). ^13^C NMR (100 MHz, CDCl_3_) δ 160.63 (d, *J* = 246.6 Hz), 133.98, 131.84, 131.24, 130.24 (d, *J* = 4.2 Hz), 129.40 (d, *J* = 8.1 Hz), 128.98, 124.96 (d, *J* = 14.6 Hz), 124.12 (d, *J* = 3.6 Hz), 122.07, 119.83, 119.55, 118.11, 115.19 (d, *J* = 21.5 Hz) 112.78, 107.99, 69.74, 64.48 (d, *J* = 3.9 Hz), 56.14, 51.81, 45.22, 36.88, 29.98, 27.42, 20.67, 16.43, 8.89. ^19^F NMR (377 MHz, CDCl3) δ -137.39. HRMS (ESI): Exact mass calcd for C_27_H_29_FN_2_O [M+H]^+^: 417.2264 found 417.2338.

Product **8** ((41S,13aS)-12-(((4-chlorobenzyl)oxy)methyl)-13a-ethyl-2,3,41,5,6,13a-hexahydro-1H-indolo[3,2,1-de]pyrido[3,2,1-ij][1,5]naphthyridine) was a yellow oily substance in 71% yield, mobile phase: DCM/MeOH = 25:1. ^1^H NMR (400 MHz, CDCl_3_) δ 7.69–7.67 (m, 1H), 7.48–7.46 (m, 1H), 7.27–7.23 (m, 2H), 7.21–7.19 (m, 2H), 7.17–7.14 (m, 1H), 7.13–7.11 (m, 1H), 5.04 (s, 1H), 4.76 (d, *J* = 12.8 Hz, 1Hz), 4.64–4.52 (m, 2H), 4.44 (d, *J* = 12.8 Hz, 1H), 4.11 (s, 1H), 3.38–3.33 (m, 1H), 3.27–3.21 (m, 1H), 3.08–3.99 (m, 1H), 2.75–2.64 (m, 2H), 2.53–2.48 (m, 1H), 1.98–1.93 (m, 1H), 1.77–1.72 (m, 2H), 1.46–1.40 (m, 2H), 1.18–1.11 (m, 1H), 1.01 (t, *J* = 7.5 Hz, 3H). ^13^C NMR (100 MHz, CDCl_3_) δ 136.44, 133.95, 133.35, 131.84, 131.24, 129.07, 129.03, 128.45, 122.05, 119.87, 119.51, 118.17, 112.74, 108.08, 70.39, 69.67, 56.03, 51.78, 45.19, 36.86, 30.06, 27.42, 20.70, 16.42, 8.99. HRMS (ESI): Exact mass calcd for C_27_H_29_ClN_2_O [M+H]^+^: 433.1968, found 433.2034.

Product **9** ((41S,13aS)-12-(((3-chlorobenzyl)oxy)methyl)-13a-ethyl-2,3,41,5,6,13a-hexahydro-1H-indolo[3,2,1-de]pyrido[3,2,1-ij][1,5]naphthyridine) was a yellow oily substance in 82% yield, mobile phase: DCM/MeOH = 30:1. ^1^H NMR (400 MHz, CDCl_3_) δ 7.57–7.55 (m, 1H), 7.36–7.34 (m, 1H), 7.16 (s, 1H), 7.14–7.10 (m, 3H), 7.08–7.05 (m, 1H), 7.03–6.99 (m, 1H), 4.93 (s, 1H), 4.65 (d, *J* = 12.9 Hz, 1H), 4.54–4.44 (m, 2H), 4.33 (d, *J* = 12.9 Hz, 1H), 4.02 (s, 1H), 3.28–3.21 (m, 1H), 3.17–3.10 (m, 1H), 2.96–2.87 (m, 1H), 2.66–2.55 (m, 2H), 2.45–2.39 (m, 1H), 1.86–1.78 (m, 1H), 1.68–1.59 (m, 2H), 1.36–1.28 (m, 2H), 1.07–0.99 (m, 1H), 0.88 (t, *J* = 7.5 Hz, 3H). ^13^C NMR (100 MHz, CDCl_3_) δ 140.07, 134.34, 134.06, 131.92, 130.85, 129.71, 128.98, 127.86, 125.81, 122.30, 120.04, 119.51, 118.29, 112.83, 108.12, 70.52, 69.76, 56.21, 51.84, 45.22, 37.00, 29.94, 27.44, 20.55, 16.46, 8.98. HRMS (ESI): Exact mass calcd for C_27_H_29_ClN_2_O [M+H]^+^: 433.1968, found 433.2035.

Product **10** ((41S,13aS)-12-(((2-chlorobenzyl)oxy)methyl)-13a-ethyl-2,3,41,5,6,13a-hexahydro-1H-indolo[3,2,1-de]pyrido[3,2,1-ij][1,5]naphthyridine) was a yellow oily substance in 62% yield, mobile phase: DCM/MeOH = 30:1. ^1^H NMR (400 MHz, CDCl_3_) δ 7.69–7.67 (m, 1H), 7.48–7.46 (m, 1H), 7.26–7.11 (m, 6H), 5.04 (s, 1H), 4.76 (d, *J* = 12.8 Hz, 1H), 4.64–4.52 (m, 2H), 4.44 (d, *J* = 12.8 Hz, 1H), 4.11 (s, 1H), 3.38–3.33 (m, 1H), 3.27–3.21 (m, 1H), 3.08–2.99 (m, 1H), 2.75–2.64 (m, 2H), 2.53–2,48 (m, 1H), 1.98–1.93 (m, 1H), 1.77–1.72 (m, 2H), 1.46–1.40 (m, 2H), 1.18–1.13 (m, 1H), 1.01 (t, *J* = 7.5 Hz, 3H). ^13^C NMR (100 MHz, CDCl_3_) δ 140.08, 134.30, 133.99, 131.82, 131.20, 129.67, 129.06, 127.82, 127.80, 125.78, 122.15, 119.94, 119.62, 118.23, 112.78, 108.15, 70.43, 69.75, 56.10, 51.82, 45.22, 36.92, 30.05, 27.44, 20.69, 16.46, 8.98. HRMS (ESI): Exact mass calcd for C_27_H_29_ClN_2_O [M+H]^+^: 433.1968, found 433.2042.

Product **11** ((41S,13aS)-12-(((4-bromobenzyl)oxy)methyl)-13a-ethyl-2,3,41,5,6,13a-hexahydro-1H-indolo[3,2,1-de]pyrido[3,2,1-ij][1,5]naphthyridine) was a yellow oily substance in 82% yield, mobile phase: DCM/MeOH = 30:1. ^1^H NMR (400 MHz, CDCl_3_) δ 8.36–8.34 (m, 2H), 7.41–7.36 (m, 2H), 7.32–7.27 (m, 2H), 7.26–7.24 (m, 1H), 7.21–7.08 (m, 1H), 3.87 (s, 1H), 3.31–3.26 (m, 1H), 3.21–3.13 (m, 1H), 2.91–2.81 (m, 2H), 2.64–2.52 (m, 2H), 2.45–2.33 (m, 3H), 2.04–1.99 (m, 1H), 1.74–1.70 (m, 1H), 1.64–1.59 (m, 1H), 1.47–1.44 (m, 2H), 1.38–1.33 (m, 2H), 1.02–0.98 (m, 1H), 0.91 (t, *J* = 7.6 Hz, 3H). ^13^C NMR (100 MHz, CDCl_3_) δ 137.79, 133.99, 131.94, 131.22, 128.97, 128.38, 127.91, 127.70, 122.06, 119.82, 119.36, 118.10, 112.83, 107.96, 71.16, 69.35, 56.10, 51.79, 45.20, 36.84, 30.05, 27.42, 20.66, 16.42, 8.95. HRMS (ESI): Exact mass calcd for C_27_H_29_BrN_2_O [M+H]^+^: 477.1463, found 477.1524.

Product **12** ((41S,13aS)-12-(((3-bromobenzyl)oxy)methyl)-13a-ethyl-2,3,41,5,6,13a-hexahydro-1H-indolo[3,2,1-de]pyrido[3,2,1-ij][1,5]naphthyridine) was a yellow oily substance in 62% yield, mobile phase: DCM/MeOH = 28:1. ^1^H NMR (400 MHz, CDCl_3_) δ 7.68–7.66 (m, 1H), 7.47–7.43 (m, 2H), 7.40–7.38 (m, 1H), 7.21–7.10 (m, 4H), 5.04 (s, 1H), 4.77 (d, *J* = 12.8 Hz, 1H), 4.64–4.55 (m, 2H), 4.44 (d, *J* = 12.8 Hz, 1H), 4.11 (s, 1H), 3.38–3.33 (m, 1H), 3.28–3.21 (m, 1H), 3.08–2.99 (m, 1H), 2.77–2.70 (m, 1H), 2.67–2.64 (m, 1H), 2.54–2.49 (m, 1H), 1.96–1.91 (m, 1H), 1.77–1.70 (m, 2H), 1.46–1.40 (m, 2H), 1.18–1.10 (m, 1H), 1.00 (t, *J* = 7.5 Hz, 3H). ^13^C NMR (100 MHz, CDCl_3_) δ 140.43, 134.05, 131.88, 131.28, 130.82, 130.81, 130.04, 129.12, 126.33, 122.59, 122.23, 120.01, 119.71, 118.30, 112.85, 108.24, 70.45, 69.85, 56.20, 51.90, 45.31, 36.99, 30.09, 27.51, 20.75, 16.53, 9.03. HRMS (ESI): Exact mass calcd for C_27_H_29_BrN_2_O [M+H]^+^: 477.1463, found 477.1526.

Product **13** ((41S,13aS)-12-(((2-bromobenzyl)oxy)methyl)-13a-ethyl-2,3,41,5,6,13a-hexahydro-1H-indolo[3,2,1-de]pyrido[3,2,1-ij][1,5]naphthyridine) was a yellow oily substance in 93% yield, mobile phase: DCM/MeOH = 30:1. ^1^H NMR (400 MHz, CDCl_3_) δ 7.71–7.68 (m, 1H), 7.49–7.46 (m, 1H), 7.44–7.38 (m, 2H), 7.21–7.05 (m, 4H), 5.08 (s, 1H),4.78 (d, *J* = 12.8 Hz, 1H), 4.72–4.65 (m, 2H), 4.53 (d, *J* = 12.8 Hz, 1H), 4.12 (s, 1H), 3.36–3.31 (m, 1H), 3.25–3.14 (m, 1H), 3.05–2.96 (m, 1H), 2.74–2.67 (m, 1H), 2.65–2.62 (m, 1H), 2.51–2.45 (m, 1H), 1.97–1.90 (m, 1H), 1.75–1.70 (m, 2H), 1.45–1.37 (m, 2H), 1.17–1.091 (m, 1H), 0.99 (t, *J* = 7.5 Hz, 3H). ^13^C NMR (100 MHz, CDCl_3_) δ 137.33, 134.01, 132.41, 131.83, 131.19, 129.13, 128.99, 128.89, 127.40, 122.49, 122.15, 119.88, 119.68, 118.16, 112.81, 108.03, 70.45, 70.13, 56.08, 51.85, 45.25, 36.91, 30.06, 27.44, 20.67, 16.44, 8.98. HRMS (ESI): Exact mass calcd for C_27_H_29_BrN_2_O [M+H]^+^: 477.1463, found 477.1526.

Product **14** (4-((((41S,13aS)-13a-ethyl-2,3,41,5,6,13a-hexahydro-1H-indolo[3,2,1-de]pyrido[3,2,1-ij][1,5]naphthyridin-12-yl)methoxy)methyl)benzonitrile) was a yellow oily substance in 78% yield, mobile phase: DCM/MeOH = 25:1. ^1^H NMR (400 MHz, CDCl_3_) δ 7.68–7.66 (m, 1H), 7.55–7.53 (m, 2H), 7.48–7.46 (m, 1H), 7.36–7.34 (m, 2H), 7.20–7.10 (m, 2H), 5.05 (s, 1H), 4.82 (d, *J* = 12.8 Hz, 1H), 4.72–4.62J (m, 2H), 4.48 (d, *J* = 12.8 Hz, 1H), 4.10 (s, 1H), 3.43–3.34 (m, 1H), 3.29–3.20 (m, 1H), 3.07–2.98 (m, 1H), 2.75–2.65 (m, 2H), 2.54–2.48 (m, 1H), 1.97–1.90 (m, 1H), 1.78–1.69 (m, 2H), 1.46–1.39 (m, 2H), 1.16–1.09 (m, 1H), 0.99 (t, *J* = 7.5 Hz, 3H). ^13^C NMR (100 MHz, CDCl_3_) δ 143.62, 133.98, 132.16, 131.64, 131.17, 129.09, 127.84, 122.20, 120.05, 119.88, 118.85, 118.34, 112.67, 111.31, 108.30, 70.28, 70.17, 56.05, 51.84, 45.25, 37.00, 30.12, 27.46, 20.68, 16.45, 9.03. HRMS (ESI): Exact mass calcd for C_28_H_29_N_3_O [M+H]^+^: 424.2311, found 424.2390.

Product **15** (2-((((41S,13aS)-13a-ethyl-2,3,41,5,6,13a-hexahydro-1H-indolo[3,2,1-de]pyrido[3,2,1-ij][1,5]naphthyridin-12-yl)methoxy)methyl)benzonitrile) was a yellow oily substance in 76% yield, mobile phase: DCM/MeOH = 25:1. ^1^H NMR (400 MHz, CDCl_3_) δ 7.70–7.68 (m, 1H), 7.61–7.59 (m, 1H), 7.46–7.45 (m, 3H), 7.35–7.31 (m, 1H), 7.21–7.17 (m, 1H), 7.14–7.10 (m, 1H), 5.14 (s, 1H), 4.86–4.79 (m, 3H), 4.56 (d, *J* = 12.9 Hz, 1H), 4.16 (s, 1H), 3.42–3.37 (m, 1H), 3.30–3.22 (m, 1H), 3.07–2.98 (m, 1H), 2.76–2.72 (m, 2H), 2.59–2.53 (m, 1H), 2.01–1.93 (m, 1H), 1.82–1.77 (m, 2H), 1.51–1.41 (m, 2H), 1.18–1.10 (m, 1H), 1.02 (t, *J* = 7.5 Hz, 3H). ^13^C NMR (100 MHz, CDCl_3_) δ 141.62, 134.00, 132.82, 132.51, 131.62, 130.36, 128.77, 128.60, 128.01, 122.37, 120.03, 119.67, 118.23, 117.23, 112.69, 111.07, 107.95, 70.36, 68.68, 56.21, 51.78, 45.15, 36.96, 29.63, 27.31, 20.27, 16.31, 8.87. HRMS (ESI): Exact mass calcd for C_28_H_29_N_3_O [M+H]^+^: 424.2311, found 424.2393.

Product **16** ((41S,13aS)-13a-ethyl-12-(((4-(trifluoromethyl)benzyl)oxy)methyl)-2,3,41,5,6,13a-hexahydro-1H-indolo[3,2,1-de]pyrido[3,2,1-ij][1,5]naphthyridine) was a yellow oily substance in 82% yield, mobile phase: DCM/MeOH = 20:1. ^1^H NMR (400 MHz, CDCl_3_) δ 7.76–7.74 (m, 1H), 7.57–7.55 (m, 2H), 7.52–7.50 (m, 1H), 7.41–7.39 (m,2H), 7.25–7.21 (m, 1H), 7.19–7.15 (m, 1H), 5.09 (s, 1H), 4.85 (d, *J* = 12.9 Hz, 1H), 4.76–4.66 (m, 2H), 4.51 (d, *J* = 12.9 Hz, 1H), 4.13 (s, 1H), 3.40–3.36 (m, 1H), 3.30–3.22 (m, 1H), 3.09–3.03 (m, 1H), 2.79–2.73 (m, 1H), 2.70 (s, 1H), 2.56–2.50 (m, 1H), 2.02–1.97 (m, 1H), 1.80–1.75 (m, 2H), 1.49–1.42 (m, 2H), 1.21–1.14 (m, 1H), 1.04 (t, *J* = 7.5 Hz, 3H). ^13^C NMR (100 MHz, CDCl_3_): δ 142.19, 133.98, 131.77, 131.19, 129.71 (q, *J* = 32.6 Hz), 129.07, 127.61, 125.21 (q, *J* = 3.9 Hz), 124.20 (q, *J* = 270.5 Hz), 122.11, 119.95, 119.67, 118.24, 112.72, 108.15, 70.33, 70.02, 55.96, 51.75, 45.18, 36.90, 30.10, 27.41, 20.65, 16.38, 8.96; ^19^F NMR (376 MHz, CDCl_3_): δ -62.30. HRMS (ESI): Exact mass calcd for C_28_H_29_F_3_N_2_O [M+H]^+^: 467.2232, found 467.2327.

Product **17** ((41S,13aS)-13a-ethyl-12-(((3-(trifluoromethyl)benzyl)oxy)methyl)-2,3,41,5,6,13a-hexahydro-1H-indolo[3,2,1-de]pyrido[3,2,1-ij][1,5]naphthyridine) was a yellow oily substance, in 74% yield, mobile phase: DCM/MeOH = 20:1. ^1^H NMR (400 MHz, CDCl_3_) δ 7.76–7.74 (m, 1H), 7.60 (s, 1H), 7.55–7.48 (m, 3H), 7.43–7.40 (m, 1H), 7.25–7.21 (m, 1H), 7.18–7.14 (m, 1H), 5.09 (s, 1H), 4.82 (d, *J* = 12.8 Hz, 1H), 4.74–4.66 (m, 2H), 4.51 (d, *J* = 12.8 Hz, 1H), 4.16 (s, 1H), 3,41–3.36 (m, 1H), 3.30–3.22 (m, 1H), 3.11–3.02 (m, 1H), 2.79–2.67 (m, 2H), 2.56–2.50 (m, 1H), 2.01–2.96 (m, 1H), 1.82–1.74 (m, 2H), 1.50–1.42 (m, 2H), 1.21–1.14 (m, 1H), 1.04 (t, *J* = 7.5 Hz, 3H). ^13^C NMR (100 MHz, CDCl_3_) δ 139.05, 133.98, 131.71, 131.19, 130.92, 130.71 (q, *J* = 32.3 Hz), 129.07, 128.86, 124.44 (q, *J* = 3.8 Hz), 124.32 (q, *J* = 3.8 Hz), 124.13 (q, *J* = 272.4 Hz), 122.14, 119.97, 119.70, 118.24, 112.72, 108.19, 70.30, 69.86, 56.11, 51.82, 45.23, 36.94, 30.06, 27.43, 20.68, 16.43, 8.91. ^19^F NMR (377 MHz, CDCl_3_) δ -62.55. HRMS (ESI): Exact mass calcd for C_28_H_29_F_3_N_2_O [M+H]^+^: 467.2232, found 467.2294.

Product **18** ((41S,13aS)-12-(([1,1′-biphenyl]-4-ylmethoxy)methyl)-13a-ethyl-2,3,41,5,6,13a-hexahydro-1H-indolo[3,2,1-de]pyrido[3,2,1-ij][1,5]naphthyridine) was a yellow oily substance in 63% yield, mobile phase: DCM/MeOH = 22:1. ^1^H NMR (400 MHz, CDCl_3_) δ 7.76–7.74 (m, 1H), 7.62–7.56 (m, 4H), 7.51–7.36 (m, 6H), 7.24–7.20 (m, 1H), 7.18–7.14 (m, 1H), 5.11 (s, 1H), 4.80 (d, *J* = 12.8 Hz, 1H), 4.75–4.70 (m, 2H), 4.52 (d, *J* = 12.8 Hz, 1H), 4.18 (s, 1H), 3.38–3.34 (m, 1H), 3.29–3.21 (m, 1H), 3.10–3.03 (m, 1H), 2.79–2.73 (m, 1H), 2.70 (s, 1H), 2.56–2.51 (m, 1H), 2.02–1.96 (m, 1H), 1.81–1.76 (m, 2H), 1.50–1.42 (m, 2H), 1.23–1.16 (m, 1H), 1.05 (t, *J* = 7.5 Hz, 3H). ^13^C NMR (100 MHz, CDCl_3_) δ 140.88, 140.70, 136.94, 134.08, 132.06, 131.24, 129.04, 128.86, 128.44, 127.39, 127.17, 127.13, 122.16, 119.92, 119.48, 118.20, 112.93, 108.05, 71.01, 69.56, 56.16, 51.82, 45.25, 36.95, 30.09, 27.49, 20.70, 16.48, 9.03. HRMS (ESI): Exact mass calcd for C_33_H_34_N_2_O [M+H]^+^: 475.2671, found 475.2754.

Product **19** ((41S,13aS)-13a-ethyl-12-((naphthalen-1-ylmethoxy)methyl)-2,3,41,5,6,13a-hexahydro-1H-indolo[3,2,1-de]pyrido[3,2,1-ij][1,5]naphthyridine) was a yellow solid in 58% yield (m.p. 86–88 °C), mobile phase: DCM/MeOH = 30:1. ^1^H NMR (400 MHz, CDCl_3_) δ 8.03–8.01 (m, 1H), 7.87–7.81 (m, 2H), 7.69–7.67 (m, 1H), 7.52–7.41 (m, 4H), 7.38–7.34 (m, 1H), 7.14–7.07 (m, 2H), 5.14–5.09 (m, 3H), 4.83 (dd, *J* = 12.9, 0.6 Hz, 1H), 4.52 (d, *J* = 12.9 Hz, 1H), 4.13 (s, 1H), 3.39–3.34 (m, 1H), 3.28–3.21 (m, 1H), 3.07–2.99 (m, 1H), 2.75–2.68 (m, 2fH), 2.55–2.49 (m, 1H), 1.96–1.88 (m, 1H), 1.80–1.72 (m, 2H), 1.48–1.40 (m, 2H), 1.32–1.12 (m, 1H), 1.01 (t, *J* = 7.5 Hz, 3H). ^13^C NMR (100 MHz, CDCl_3_) δ 133.95, 133.67, 133.24, 132.02, 131.72, 131.17, 128.95, 128.70, 128.36, 126.74, 126.04, 125.74, 125.05, 124.13, 121.99, 119.75, 119.36, 118.01, 112.90, 107.91, 69.79, 69.43, 56.07, 51.73, 45.14, 36.76, 29.90, 27.35, 20.66, 16.38, 8.90. HRMS (ESI): Exact mass calcd for C_31_H_32_N_2_O [M+H]^+^: 449.2515, found 449.2588.

Product **20** ((41S,13aS)-13a-ethyl-12-((naphthalen-2-ylmethoxy)methyl)-2,3,41,5,6,13a-hexahydro-1H-indolo[3,2,1-de]pyrido[3,2,1-ij][1,5]naphthyridine) was a yellow solid in 79% yield, MP: 89–91 °C, mobile phase: DCM/MeOH = 30:1. ^1^H NMR (400 MHz, CDCl_3_) δ 7.84–7.81 (m, 2H), 7.77–7.75 (m, 2H), 7.72–7.70 (m, 2H), 7.49–7.47 (m, 2H), 7.41–7.39 (m, 1H), 7.23–7.14 (m, 2H), 5.06 (s, 1H), 4.88–4.84 (m, 2H), 4.77 (d, *J* = 12.9 Hz, 1H), 4.47 (d, *J* = 12.9 Hz, 1H), 3.96 (s, 1H), 3.32–3.25 (m, 1H), 3.11–2.95 (m, 2H), 2.74–2.63 (m, 2H), 2.48–2.41 (m, 1H), 1.97–1.89 (m, 1H), 1.78–1.66 (m, 2H), 1.45–1.38 (m, 2H), 1.17–1.10 (m, 1H), 1.00 (t, *J* = 7.5 Hz, 3H). ^13^C NMR (100 MHz, CDCl_3_) δ δ 135.53, 134.08, 133.24, 133.02, 132.16, 131.23, 129.08, 128.09, 127.94, 127.69, 126.64, 126.08, 125.96, 125.84, 122.13, 119.90, 119.46, 118.18, 112.98, 108.03, 71.54, 69.78, 56.07, 51.74, 45.22, 36.85, 29.98, 27.44, 20.72, 16.44, 9.01. HRMS (ESI): Exact mass calcd for C_31_H_32_N_2_O [M+H]^+^: 449.2515, found 449.2609.

Product **21** ((41S,13aS)-12-(((3,5-dimethylbenzyl)oxy)methyl)-13a-ethyl-2,3,41,5,6,13a-hexahydro-1H-indolo[3,2,1-de]pyrido[3,2,1-ij][1,5]naphthyridine) was a yellow oily substance in 75% yield, mobile phase: DCM/MeOH = 25:1. ^1^H NMR (400 MHz, CDCl_3_) δ 7.70–7.68 (m, 1H), 7.46–7.44 (m, 1H), 7.19–7.09 (m, 3H), 6.90 (s, 2H), 5.05 (s, 1H), 4.74 (d, *J* = 12.8 Hz, 1H), 4.65–4.52 (m, 2H), 4.43 (d, *J* = 12.8 Hz, 1H), 4.11 (s, 1H), 3.42–3.33 (m, 1H), 3.28–3.20 (m, 1H), 3.08–2.98 (m, 2H), 2.77–2.65 (m, 1H), 2.54–2.49 (m, 1H), 2.26 (s, 6H), 1.95–1.88 (m, 1H), 1.77–1.68 (m, 2H), 1.46–1.39 (m, 2H), 1.18–1.14 (m, 1H), 0.99 (t, *J* = 7.5 Hz, 3H).^13^C NMR (100 MHz, CDCl_3_) δ 137.81, 137.72, 134.00, 132.09, 131.25, 129.25, 128.96, 125.80, 121.99, 119.77, 119.26, 118.04, 112.96, 107.87, 71.34, 69.43, 56.15, 51.82, 45.19, 36.79, 29.94, 27.41, 21.25, 20.68, 16.44, 8.90. HRMS (ESI): Exact mass calcd for C_29_H_34_N_2_O [M+H]^+^: 427.2671, found 427.2759.

Product **22** ((41S,13aS)-12-(((3,5-difluorobenzyl)oxy)methyl)-13a-ethyl-2,3,41,5,6,13a-hexahydro-1H-indolo[3,2,1-de]pyrido[3,2,1-ij][1,5]naphthyridine) was a yellow oily substance in 56% yield, mobile phase: DCM/MeOH = 30:1. ^1^H NMR (400 MHz, CDCl_3_) δ 7.71–7.69 (m, 1H), 7.49–7.48 (m, 1H), 7.23–7.19 (m, 1H), 7.16–7.13 (m, 1H), 6.83–6.82 (m, 2H), 6.73–6.68 (m, 1H), 5.06 (s, 1H), 4.79 (d, *J* = 12.8 Hz, 1H), 4.66–4.56 (m, 2H), 4.48 (d, *J* = 12.8 Hz, 1H), 4.16 (s, 1H), 3.40–3.35 (m, 1H), 3.29–3.22 (m, 1H), 3.09–3.00 (m, 1H), 2.78–2.72 (m, 1H), 2.68–2.66 (m, 1H), 2.55–2.50 (m, 1H), 2.00–2.95 (m, 1H), 1.79–1.73 (m, 2H), 1.49–1.42 (m, 2H), 1.21–1.13 (m, 1H), 1.02 (t, *J* = 7.5 Hz, 3H). ^13^C NMR (100 MHz, CDCl_3_) δ 163.04 (dd, *J* = 248.7, 12.6 Hz), 142.17 (t, *J* = 8.8 Hz), 133.96, 131.62, 131.19, 129.08, 122.19, 120.01, 119.79, 118.29, 112.66, 110.17 (d, *J* = 6.9 Hz), 109.98 (d, *J* = 6.8 Hz), 108.24, 102.88 (t, *J* = 25.3 Hz), 69.88, 69.84, 56.03, 51.81, 45.22, 36.98, 30.14, 27.44, 20.69, 16.44, 8.97. ^19^F NMR (377 MHz, CDCl_3_) δ -109.69. HRMS (ESI): Exact mass calcd for C_27_H_28_F_2_N_2_O[M+H]^+^: 435.2170, found 435.2242.

Product **23** ((41S,13aS)-12-(((3,5-dibromobenzyl)oxy)methyl)-13a-ethyl-2,3,41,5,6,13a-hexahydro-1H-indolo[3,2,1-de]pyrido[3,2,1-ij][1,5]naphthyridine) was a yellow oily substance in 81% yield, mobile phase: DCM/MeOH = 22:1. ^1^H NMR (400 MHz, CDCl_3_) δ 7.67–7.65 (m, 1H), 7.54–7.53 (m, 1H), 7.47–7.45 (m, 1H), 7.30–7.29 (m, 2H), 7.22–7.18 (m, 1H), 7.15–7.11 (m, 1H), 5.02 (s, 1H), 4.84 (d, *J* = 12.9 Hz, 1H), 4.65–4.59 (m, 1H), 4.51 (d, *J* = 12.9 Hz, 1H), 4.43–4.39 (m, 1H), 4.05 (s, 1H), 3.38–3.33 (m, 1H), 3.28–3.20 (m, 1H), 3.07–2.99 (m, 1H), 2.73–2.65 (m, 2H), 2.55–2.49 (m, 1H), 1.95–1.90 (m, 1H), 1.77–1.70 (m, 2H), 1.43–1.42 (m, 2H), 1.15–1.07 (m, 1H), 0.99 (t, *J* = 7.5 Hz, 3H). ^13^C NMR (100 MHz, CDCl_3_) δ 142.26, 133.98, 133.17, 131.77, 131.07, 129.27, 129.15, 128.43, 122.93, 122.28, 120.08, 119.83, 118.42, 112.75, 108.38, 70.27, 69.83, 56.14, 51.88, 45.26, 37.00, 30.01, 27.48, 20.70, 16.53, 9.04. HRMS (ESI): Exact mass calcd for C_27_H_28_Br_2_N_2_O [M+H]^+^: 555.0568, found 555.0635.

Product **24** ((41S,13aS)-12-(((1,3-dimethyl-1H-pyrazol-5-yl)methoxy)methyl)-13a-ethyl-2,3,41,5,6,13a-hexahydro-1H-indolo[3,2,1-de]pyrido[3,2,1-ij][1,5]naphthyridine) was a yellow oily substance, in 52% yield, mobile phase: DCM/MeOH = 20:1. ^1^H NMR (400 MHz, CDCl_3_) δ 7.53–7.51 (m, 1H), 7.45–7.43 (m, 1H), 7.16–7.08 (m, 3H), 5.99–5.96 (m, 1H), 5.05 (s, 1H), 4.71 (d, *J* = 12.8 Hz, 1H), 4.61–4.54 (m, 1H), 4.38 (d, *J* = 12.8 Hz, 1H), 4.20 (s, 1H), 3.83 (s, 1H), 3.61 (s, 3H), 3.40–3.35 (m, 1H), 3.31–3.23 (m, 1H), 3.07–3.00 (m, 1H), 2.77–2.71 (m, 1H), 2.57–2.52 (m, 1H), 2.23 (s, 3H), 1.98–1.91 (m, 1H), 1.81–1.74 (m, 2H), 1.50–1.41 (m, 2H), 1.18–1.13 (m, 1H), 1.01 (t, *J* = 7.5 Hz, 3H). ^13^C NMR (100 MHz, CDCl_3_) δ 147.14, 138.59, 134.05, 131.71, 128.97, 122.40, 120.15, 119.89, 118.35, 112.69, 108.21, 107.03, 105.30, 69.08, 61.15, 56.36, 51.89, 45.29, 37.09, 36.32, 29.93, 27.49, 20.57, 16.49, 13.53, 8.99. HRMS (ESI): Exact mass calcd for C_26_H_32_N_4_O [M+H]^+^: 417.5690, found 417.2663.

Product **25** (2-chloro-5-((((41S,13aS)-13a-ethyl-2,3,41,5,6,13a-hexahydro-1H-indolo[3,2,1-de]pyrido[3,2,1-ij][1,5]naphthyridin-12-yl)methoxy)methyl)thiazole) was a yellow oily substance in 86% yield, mobile phase: DCM/MeOH = 25:1. ^1^H NMR (400 MHz, CDCl_3_) δ 7.43–7.41 (m, 1H), 7.38–7.36 (m, 1H), 7.16–7.07 (m, 2H), 7.04 (s, 1H), 5.54 (d, *J* = 12.8 Hz, 1H), 5.25 (d, *J* = 12.8 Hz, 1H), 4.46 (s, 1H), 4.43 (d, *J* = 0.8 Hz, 1H), 3.57–3.52 (m, 1H), 3.43–3.39 (m, 1H), 3.31 (s, 2H), 3.08–3.00 (m, 2H), 2.89–2.82 (m, 1H), 2.79–2.73 (m, 1H), 2.12–2.06 (m, 1H), 1.94–1.87 (m, 1H), 1.51 (s, 2H),1.22–1.14 (m, 2H), 1.00 (t, *J* = 7.4 Hz, 1H). ^13^C NMR (100 MHz, CDCl_3_) δ 173.60, 135.43, 134.13, 130.84, 128.20, 128.08, 123.41, 120.60, 119.92, 118.63, 111.94, 107.74, 69.27, 66.90, 57.59, 51.72, 44.97, 37.25, 28.55, 27.15, 19.07, 16.04, 8.65. HRMS (ESI): Exact mass calcd for C_24_H_26_ClN_3_OS [M+H]^+^: 440.1485, found 440.2039.

### 3.3. Assessment of PDE1A Enzyme Inhibition

The assays were carried out as described previously. All of the enzymatic reactions were conducted at 25 °C for 1 h. The reaction mixture contains 40 mM MOPS (pH 7.5), 0.5 mM EDTA, 15 mM MgCl_2_, 0.15 mg/mL BSA, 1 mM DTT, 0.05% Proclin 200, 15 ng/mL PDE1A, and 100 nM FAM-cyclic-3′, 5′-AMP. The compounds were dissolved in 10% DMSO, and 5 μL of the dilution was added to a 50 μL reaction for a final concentration of 1% DMSO in all reactions. The reaction mixture was incubated at 25 °C for 1 h. Then, 100 μL of diluted binding agent was added to each well and incubated at 25 °C for 1 h with slow shaking. The fluorescence polarization of the sample was detected using an excitation filter of 360 nm and an emission filter of 480 nm. The IC_50_ values were calculated using non-linear regression with a normalized dose–response fit using Prism 6 (GraphPad Software, San Diego, CA, USA).

### 3.4. Docking Studies

The LigPrep module in Schrödinger Suite was employed for the preparation of compounds, with OPLS3 utilized for energy minimization while preserving the molecular chirality. The protein model for PDE1A was obtained using AlphaFold. Subsequently, the Protein Preparation Wizard module was utilized to prepare the protein, and the protein’s active site was defined as the docking site (inner box size = 10 Å; outer box = 20 Å). Molecular docking was conducted using the Ligand Docking module. Following the initial docking, additional precision (XP) was applied in the redocking phase, with default cutoff thresholds. Ligand Docking scores were computed using the default scoring function [19].

## 4. Conclusions

This study designed and synthesized 25 novel vinpocetine derivatives, evaluating their inhibitory activity against PDE1A. The investigation revealed that seven compounds (**3**, **4**, **5**, **12**, **14**, **21**, and **25**) exhibited superior inhibition of PDE1A compared to vinpocetine, with compounds **4** and **25**, featuring meta-methylbenzene and 3-chlorothiazole substitutions, demonstrating optimal inhibitory activity with IC_50_ values of 3.53 ± 0.25 μM and 2.08 ± 0.16 μM, respectively. Molecular docking studies indicated that compounds **4** and **25**, in addition to the A and B rings interacting with the PDE1A target protein, formed extra π–π interactions and hydrogen bonds with the extended side chain, enhancing the stability of the ligand–protein complex and consequently elevating their inhibitory activity against PDE1A. In summary, based on the findings of this research, compounds **4** and **25** have been identified as promising candidates targeting the PDE1A protein. These compounds provide valuable insights for further investigations aimed at developing improved inhibitors.

## Figures and Tables

**Table 1:** Inhibition of PDE1A Activity by Compounds **2**–**25**.

Compound	IC_50_ (μM)	Compound	IC_50_ (μM)
**2**	19.22 ± 2.25	**15**	45.15 ± 2.27
**3**	8.52 ± 0.68	**16**	57.74 ± 2.96
**4**	3.53 ± 0.25	**17**	48.37 ± 1.44
**5**	17.18 ± 3.75	**18**	44.82 ± 2.26
**6**	25.58 ± 1.23	**19**	54.27 ± 1.84
**7**	28.72 ± 1.15	**20**	89.64 ± 5.34
**8**	48.82 ± 2.55	**21**	10.04 ± 0.85
**9**	72.14 ± 3.89	**22**	46.39 ± 1.94
**10**	35.02 ± 1.85	**23**	58.19 ± 3.20
**11**	54.85 ± 3.42	**24**	63.65 ± 3.85
**12**	12.47 ± 1.06	**25**	2.08 ± 0.16
**13**	88.26 ± 3.26	Vinpocetine	17.25 ± 1.21
**14**	15.65 ± 1.09		

**Table 2:** Docking of compounds **4** and **25** against PDE1A.

Compound	Structure	Docking Score	Glide Emodel (kcal/mol)
4	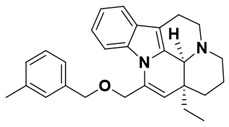	−12.027	−69.613
25	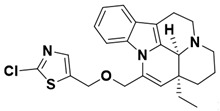	−11.326	−82.876

## Data Availability

The data presented in this study are available in this article and on request from the first author and corresponding author.

## Supplementary Materials

The following supporting information can be downloaded at: https://www.mdpi.com/article/10.3390/molecules29010014/s1, NMR and HRMS spectra.
