# Supplementary material for: Synthesis and Activity Evaluation of Vinpocetine-Derived Indole Alkaloids
Source: Molecules. 2023 Dec 19;29(1):14. doi: 10.3390/molecules29010014 (PMC10779641; doi:10.3390/molecules29010014)
Supplement: Supplementary file 1 [file molecules-29-00014-s001.zip › molecules-2772985-supplementary.pdf]

Synthesis and Activity Evaluation of Vinpocetine-Derived Indole Alkaloids

Zhang-Chao Dong[1,†], Yang Shi[1,†], Liang-Liang Zheng[1], You-Ping Tian[1], Jian Yang[3], Ying Wei[1], Ying Zhou[1,*] and Bo-Wen Pan[1,2*]

Zhang-Chao Dong [1,†], Yang Shi [1,†], Liang-Liang Zheng [1], You-Ping Tian [1], Jian Yang [2], Ying Wei [1], Ying Zhou [1,*] and Bo-Wen Pan [1,3,*]

[1] College of Pharmacy, Guizhou University of Traditional Chinese Medicine, Guiyang 550025, China; weiying1969@126.com (Y.W.); dongzhangchao0824@163.com (Z.-C.D.); xiaoyewater@163.com (Y.S.); zx15117508142@163.com (L.-L.Z.); nanshan-tia@163.com (Y.-P.T.); weiying479@gzy.edu.cn (Y.W.)

[2] College of Pharmacy and Nutrition, University of Saskatchewan, Saskatoon, SK S7N 5E5, Canada; jian.yang@usask.ca

[3] State Key Laboratory of Natural and Biomimetic Drugs, School of Pharmaceutical Sciences, Peking University, Beijing 100191, China

* Correspondence: bwpan@gzy.edu.cn (B.W.P.); zhouying067@gzy.edu.cn (Y.Z.); Tel.: +86-0851-88233090 (Y.Z.)

† These authors contributed equally to this work.

Contents   Page

1. NMR and HRMS spectra 2–79

1. NMR and HRMS spectra

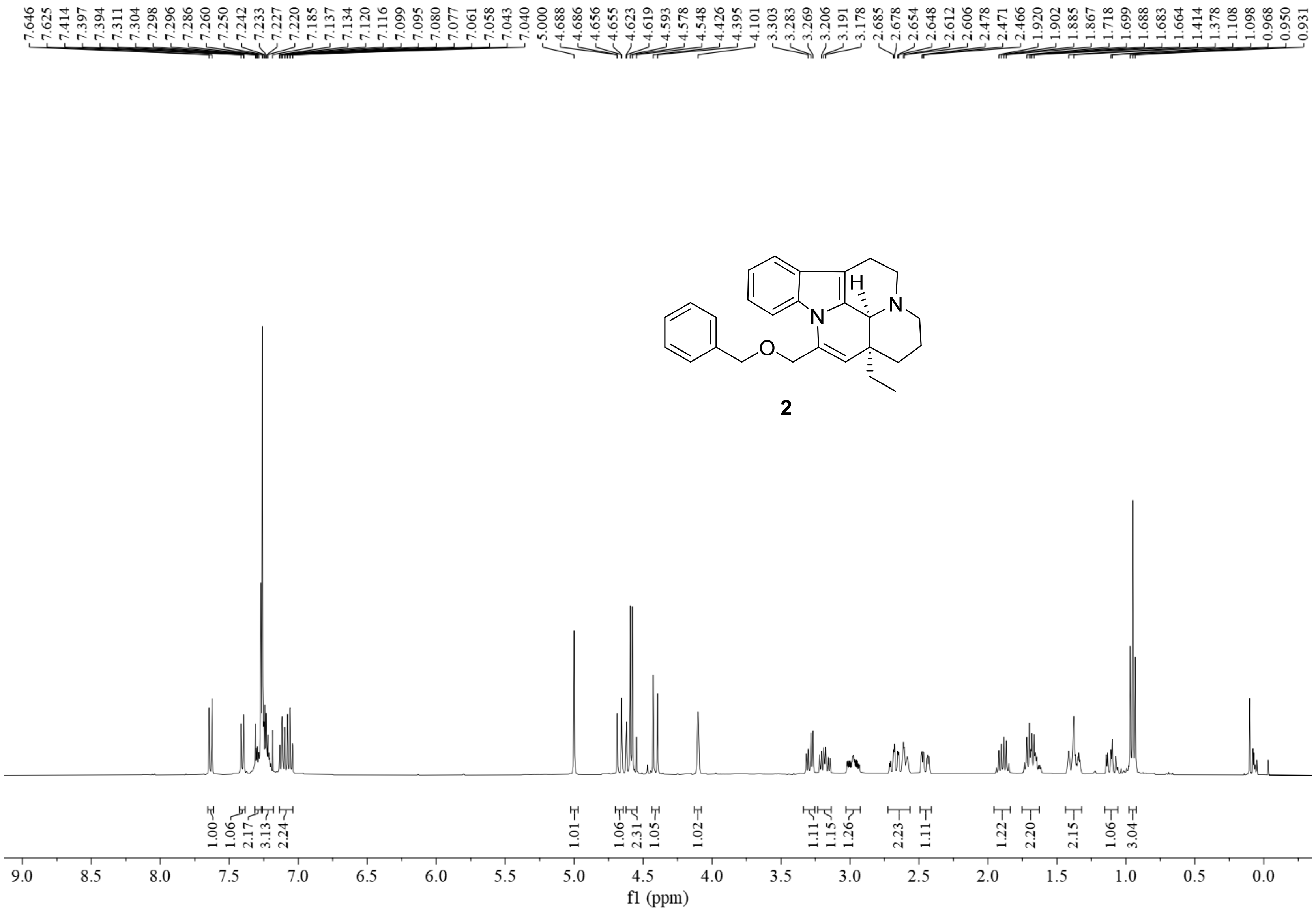

^{1}H NMR of Compound **2** (400 MHz, $CDCl_3$)

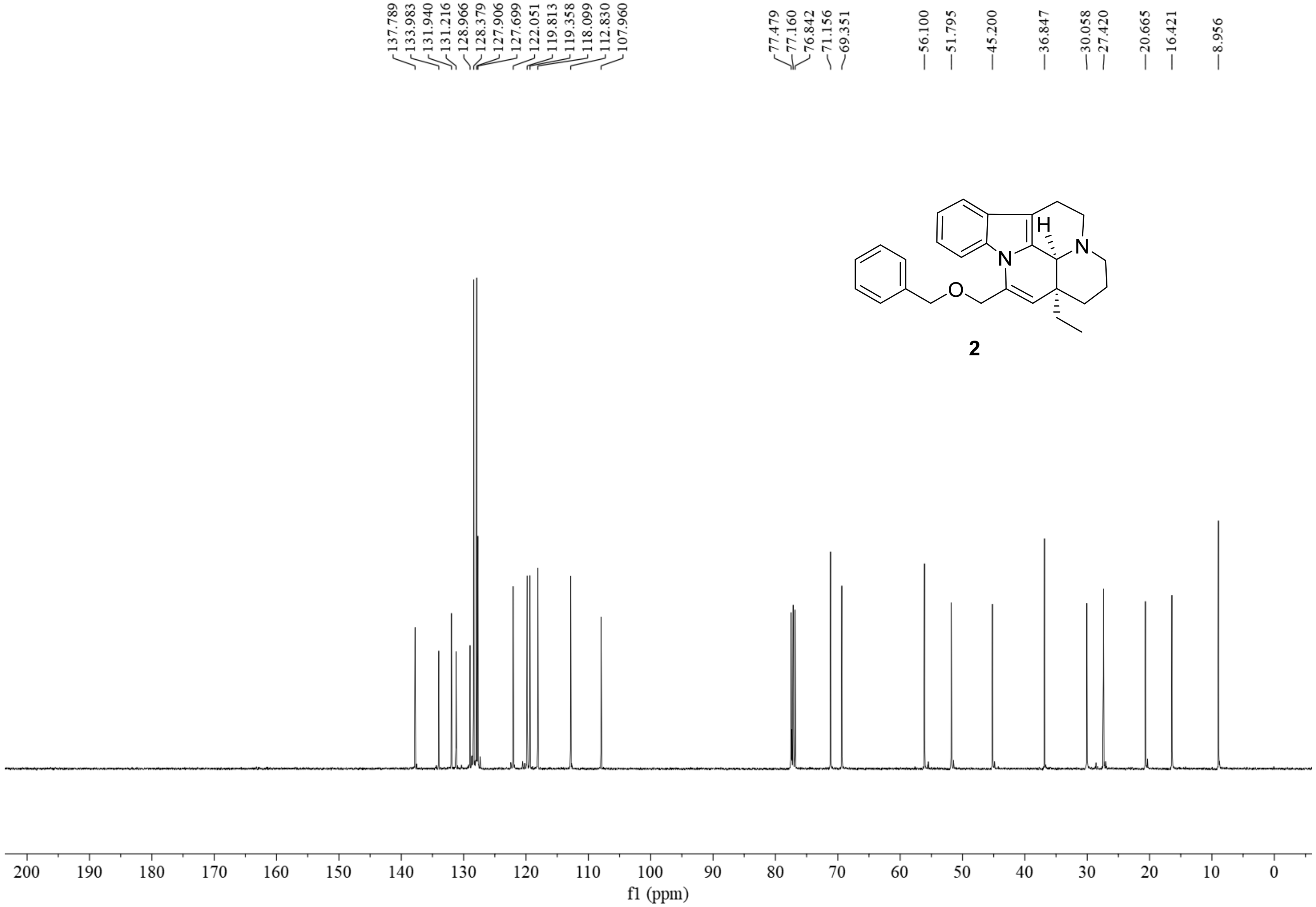

^{13}C NMR of Compound **2** (100 MHz, $CDCl_3$)

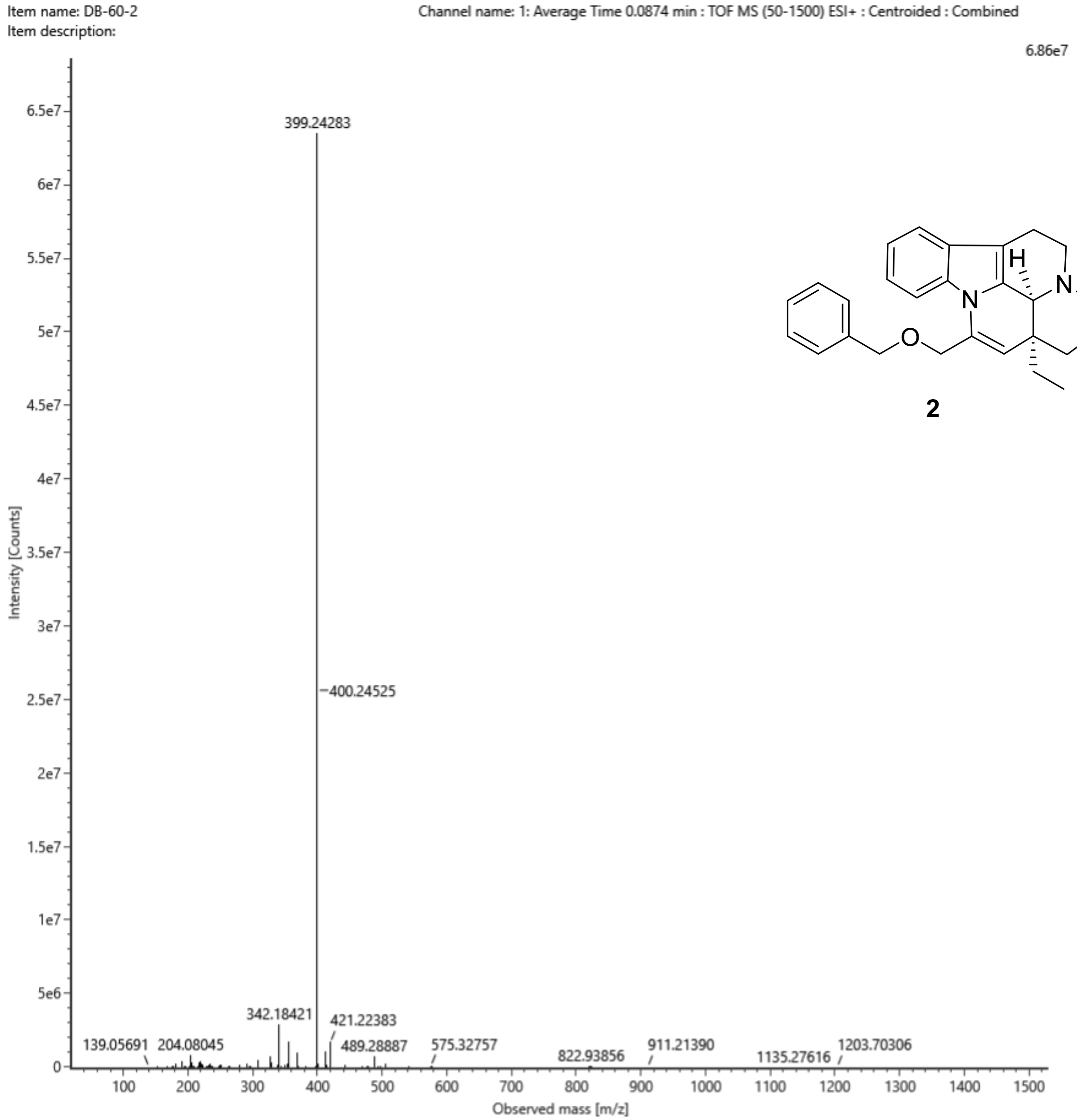

2

HRMS of Compound **2**

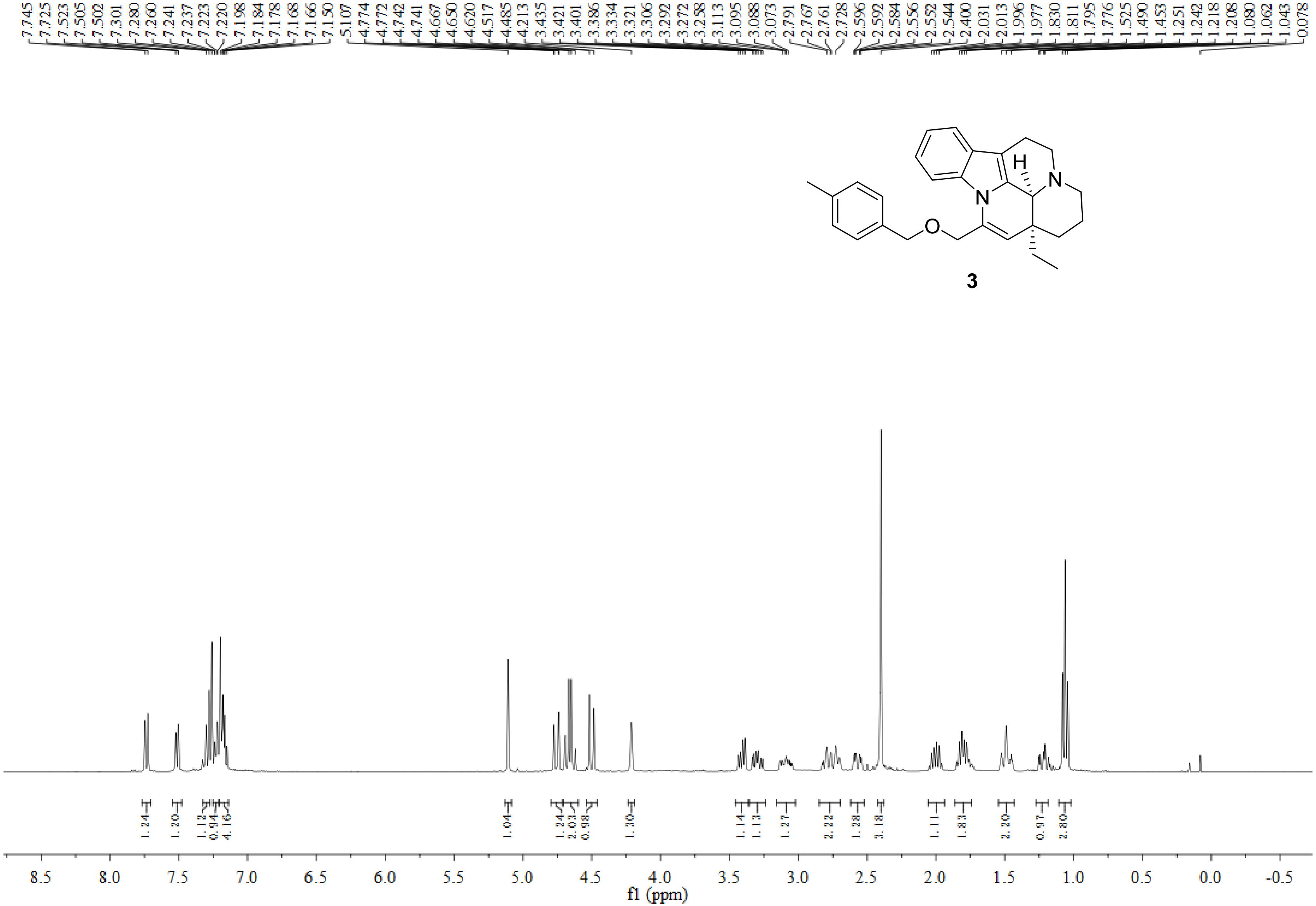

^{1}H NMR of Compound **3** (400 MHz, $CDCl_3$)

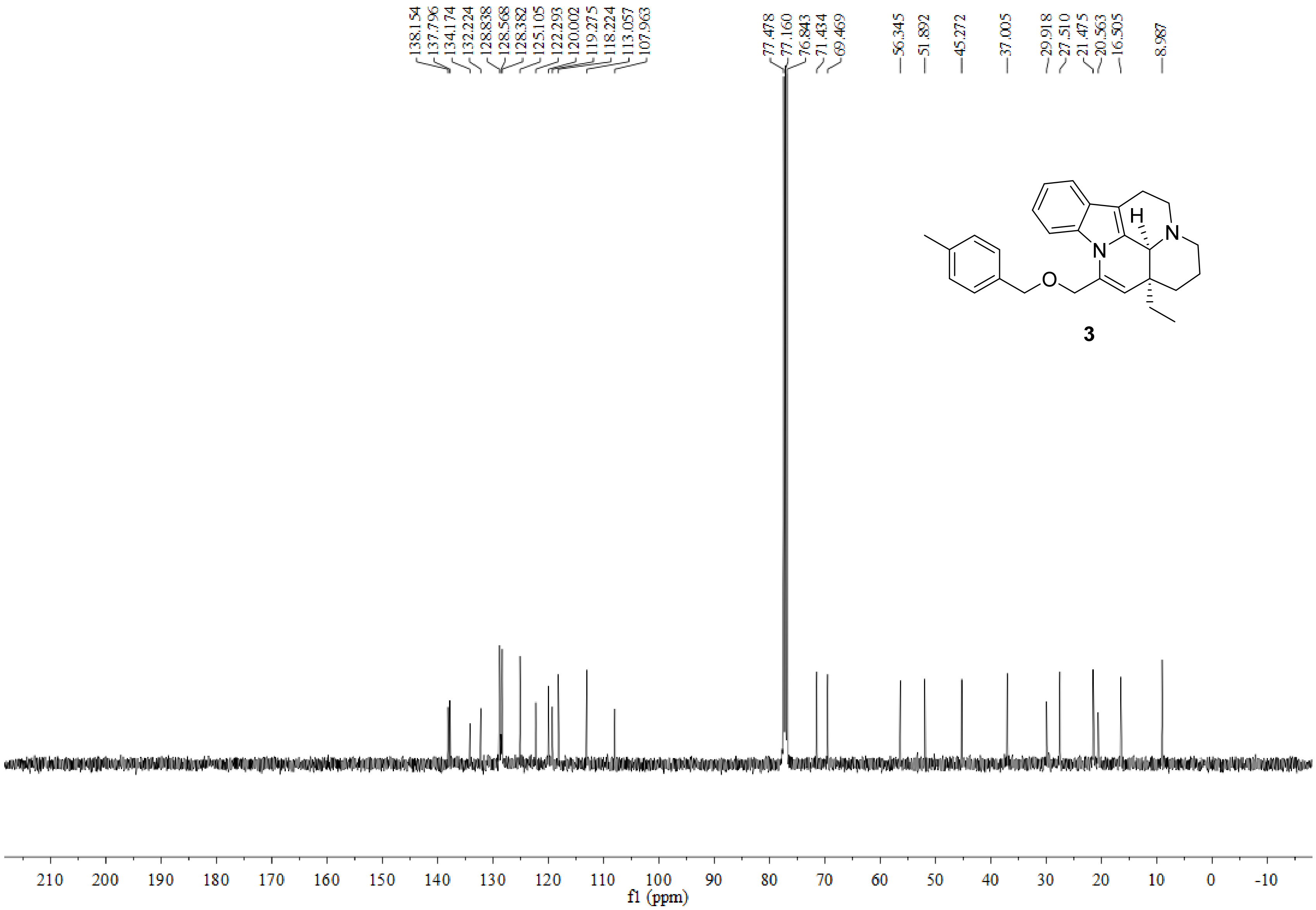

^{13}C NMR of Compound **3** (100 MHz, $CDCl_3$)

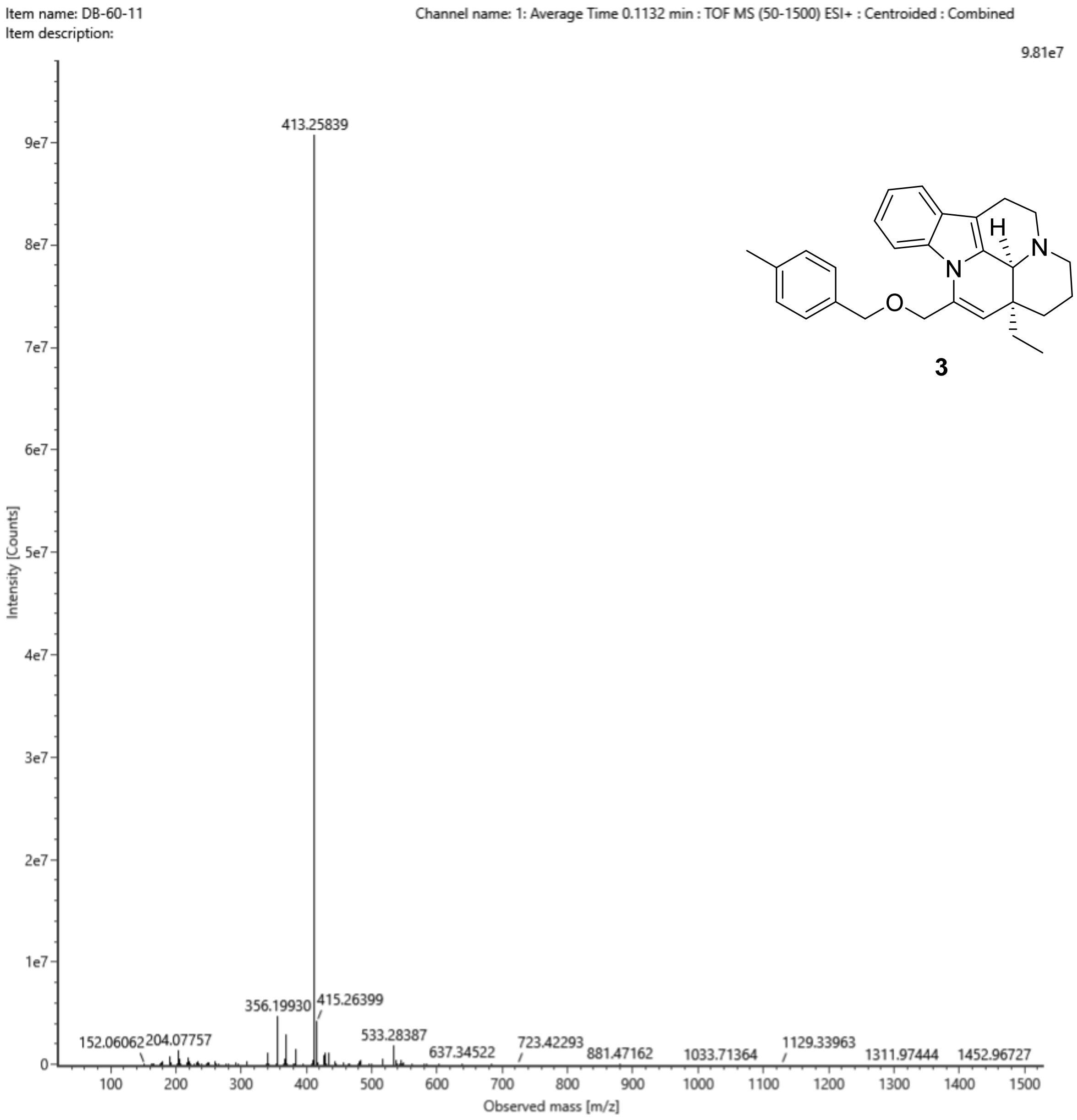

HRMS of Compound **3**

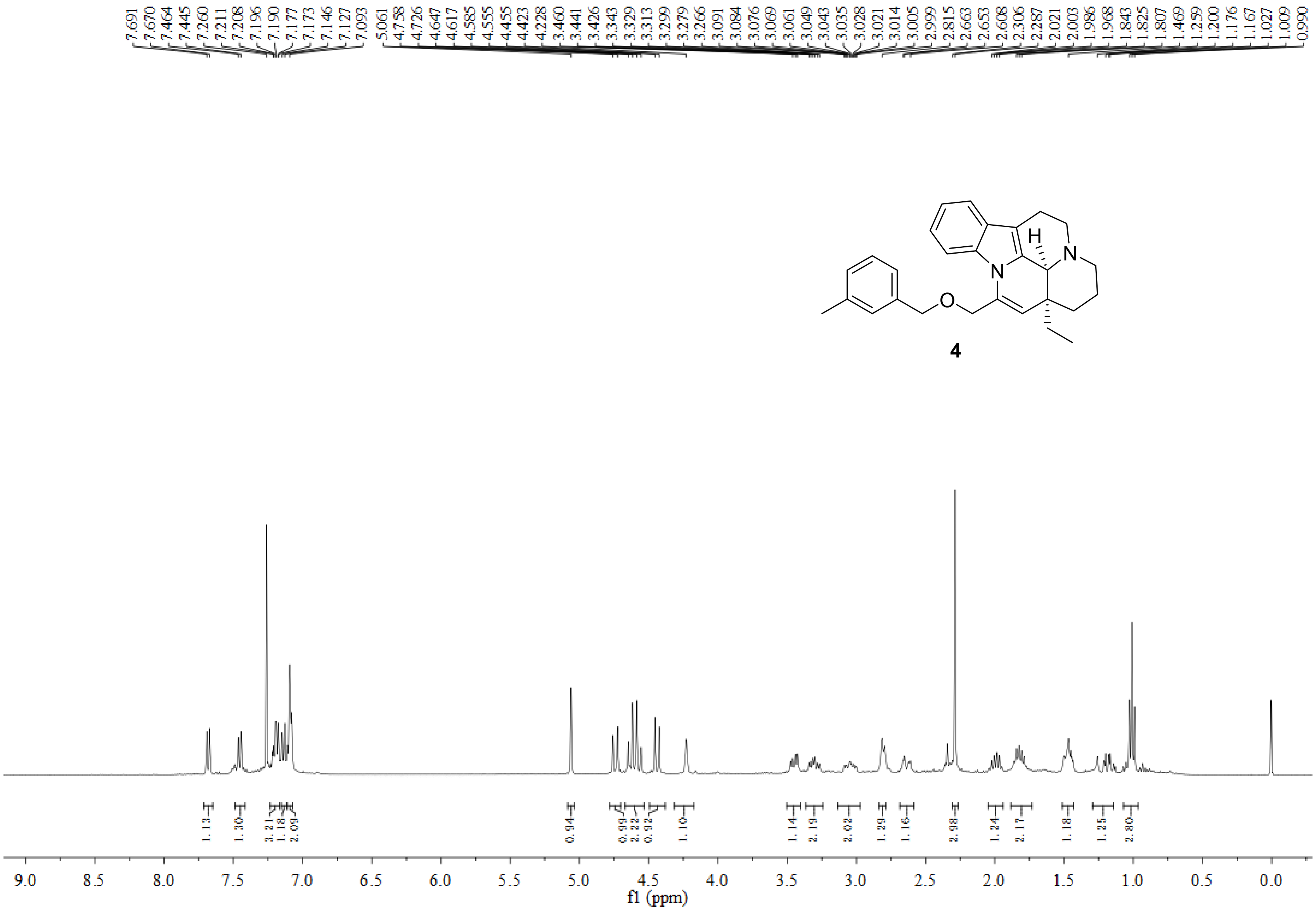

^{1}H NMR of Compound **4** (400 MHz, $CDCl_3$)

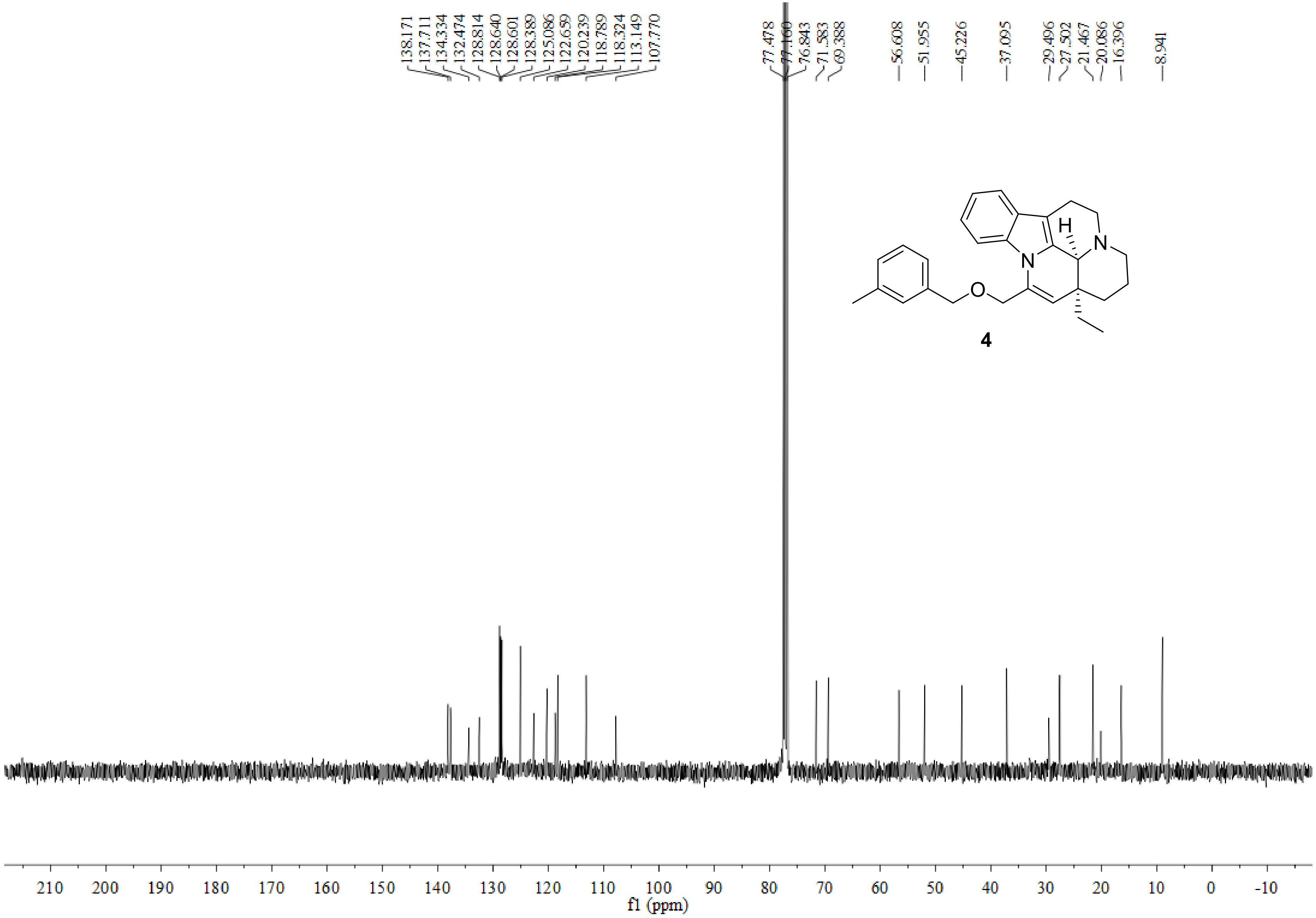

^{13}C NMR of Compound **4** (100 MHz, $CDCl_3$)

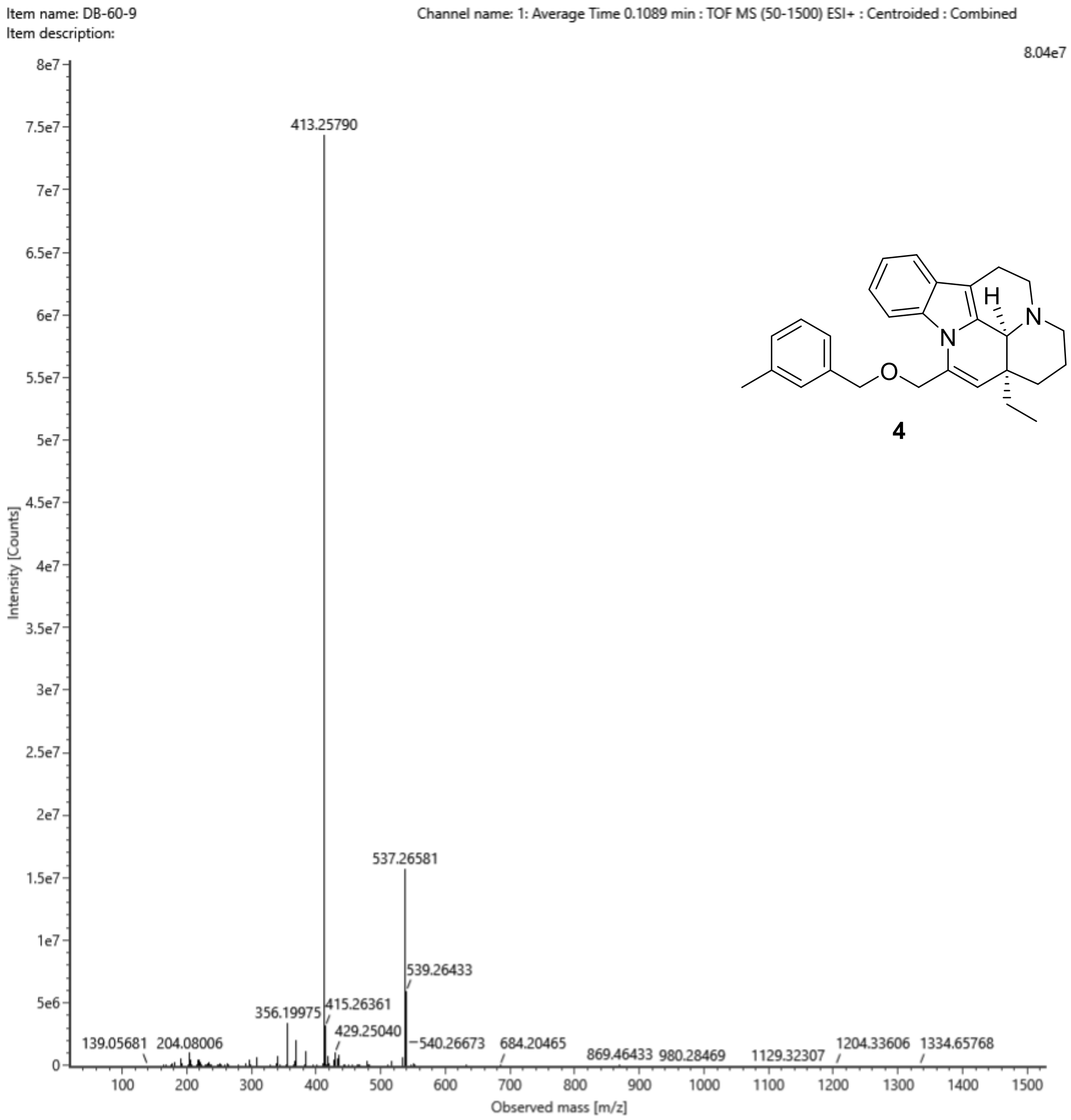

HRMS of Compound **4**

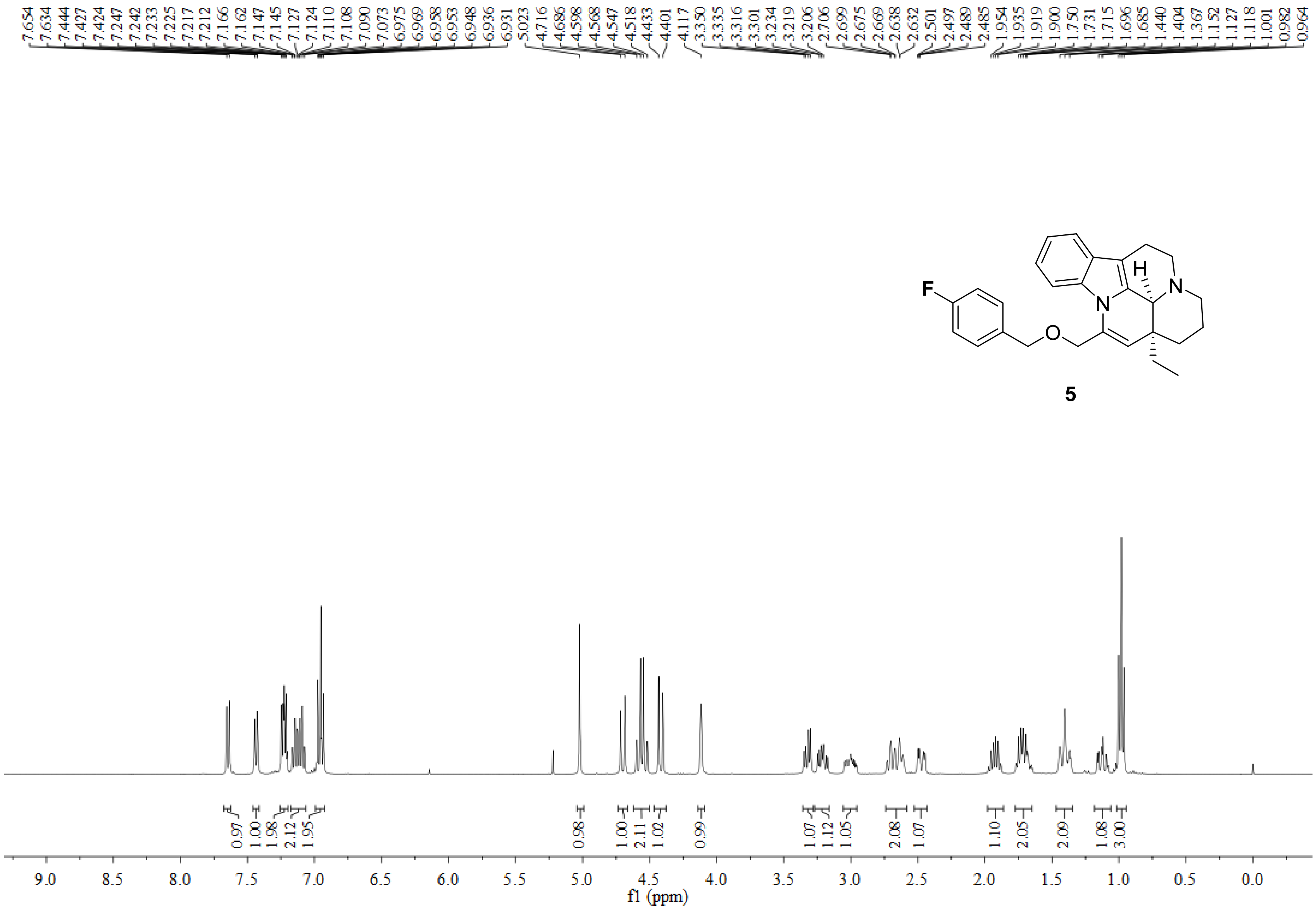

^{1}H NMR of Compound **5** (400 MHz, $CDCl_3$)

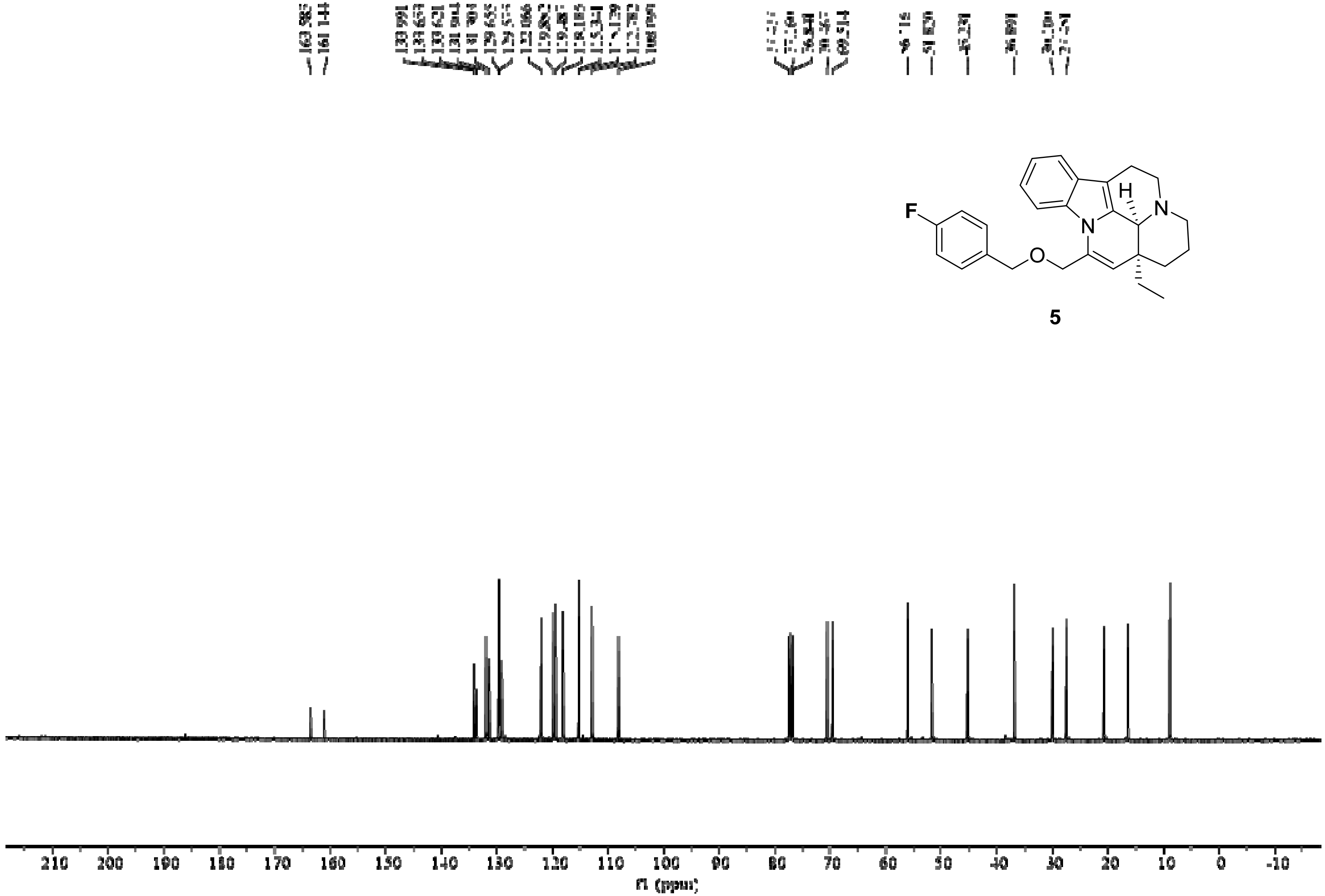

5

^{13}C NMR of Compound **5** (100 MHz, $CDCl_3$)

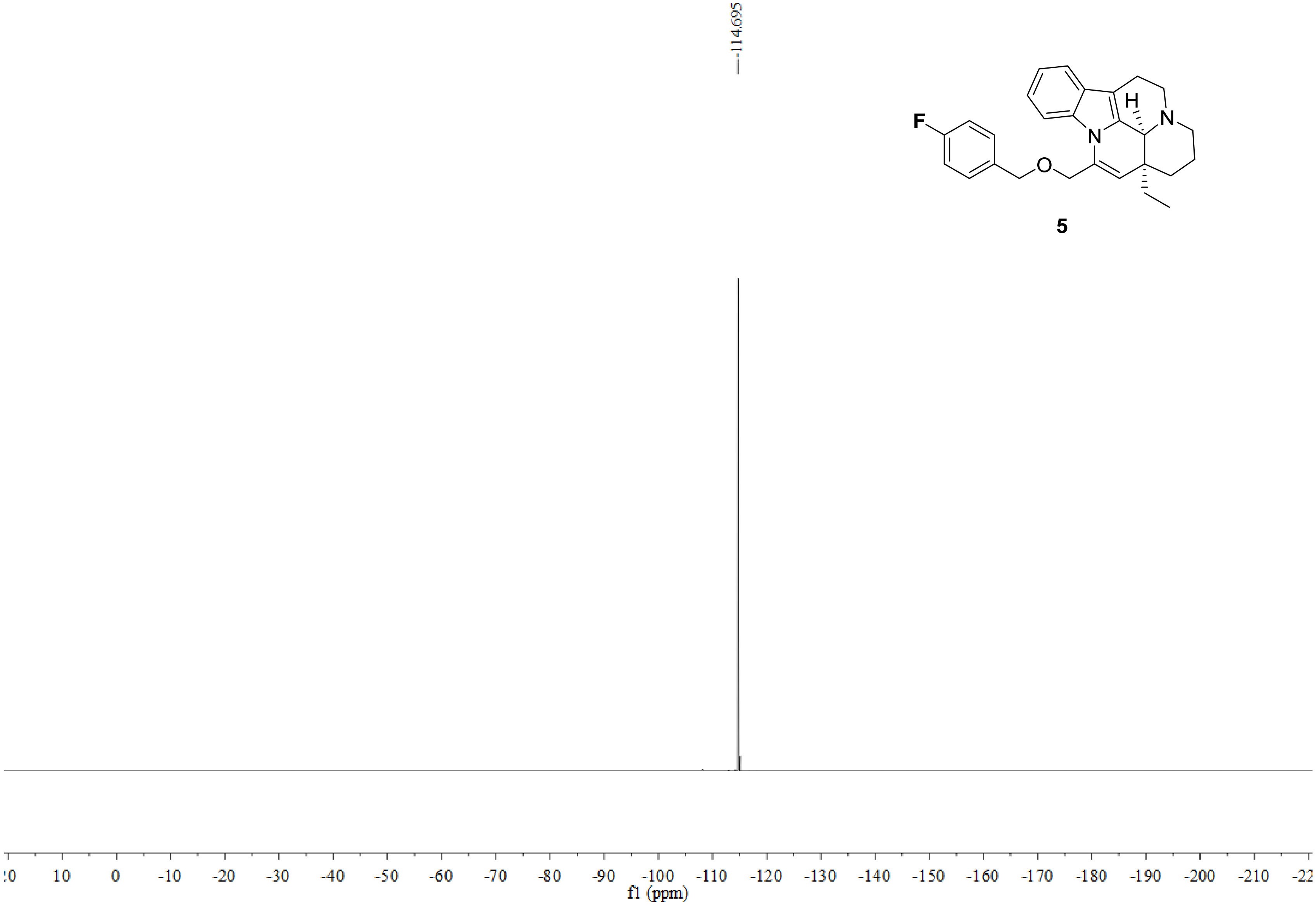

^{19}F NMR of Compound **5** (377 MHz, $CDCl_3$)

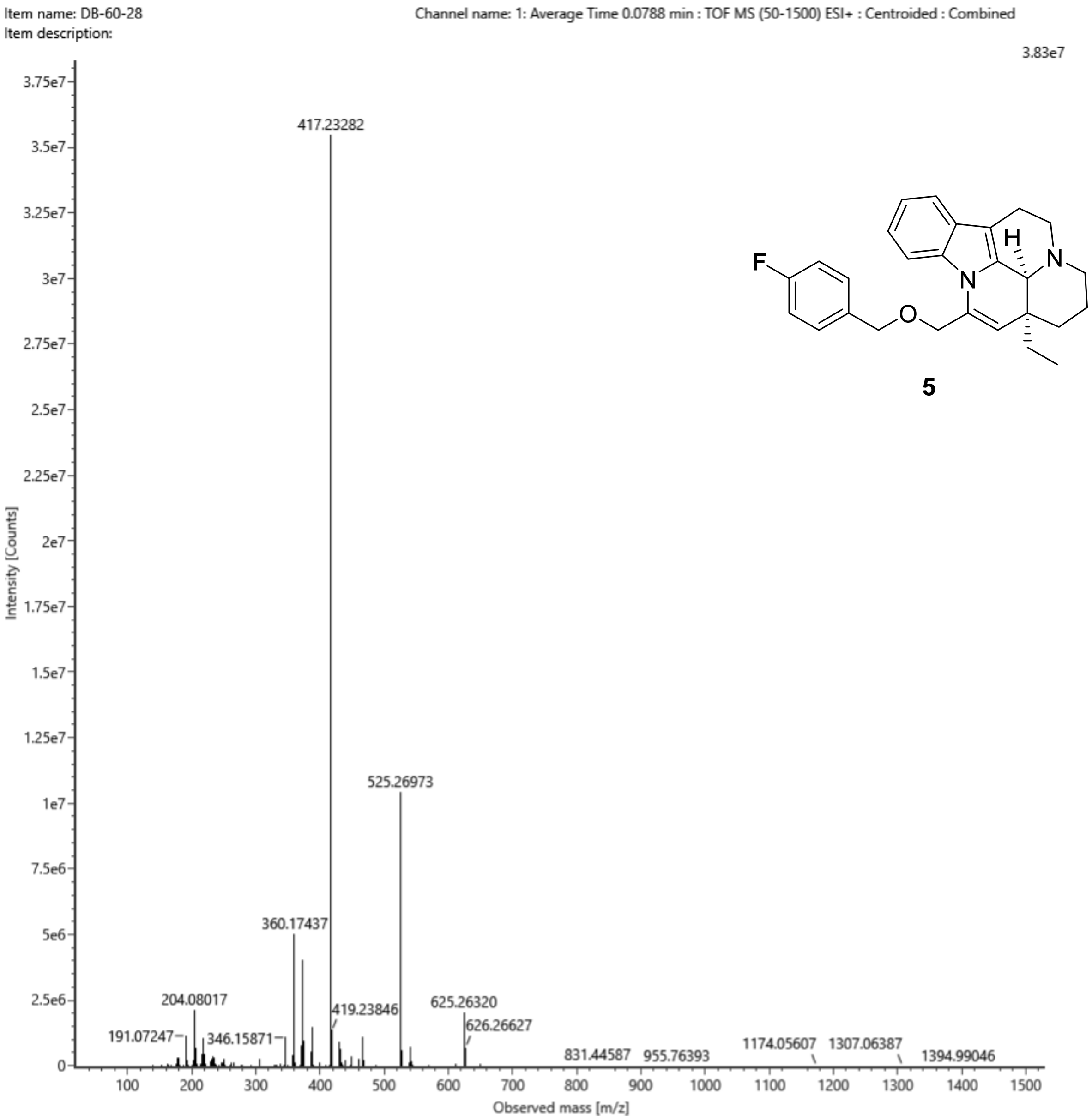

5

HRMS of Compound **5**

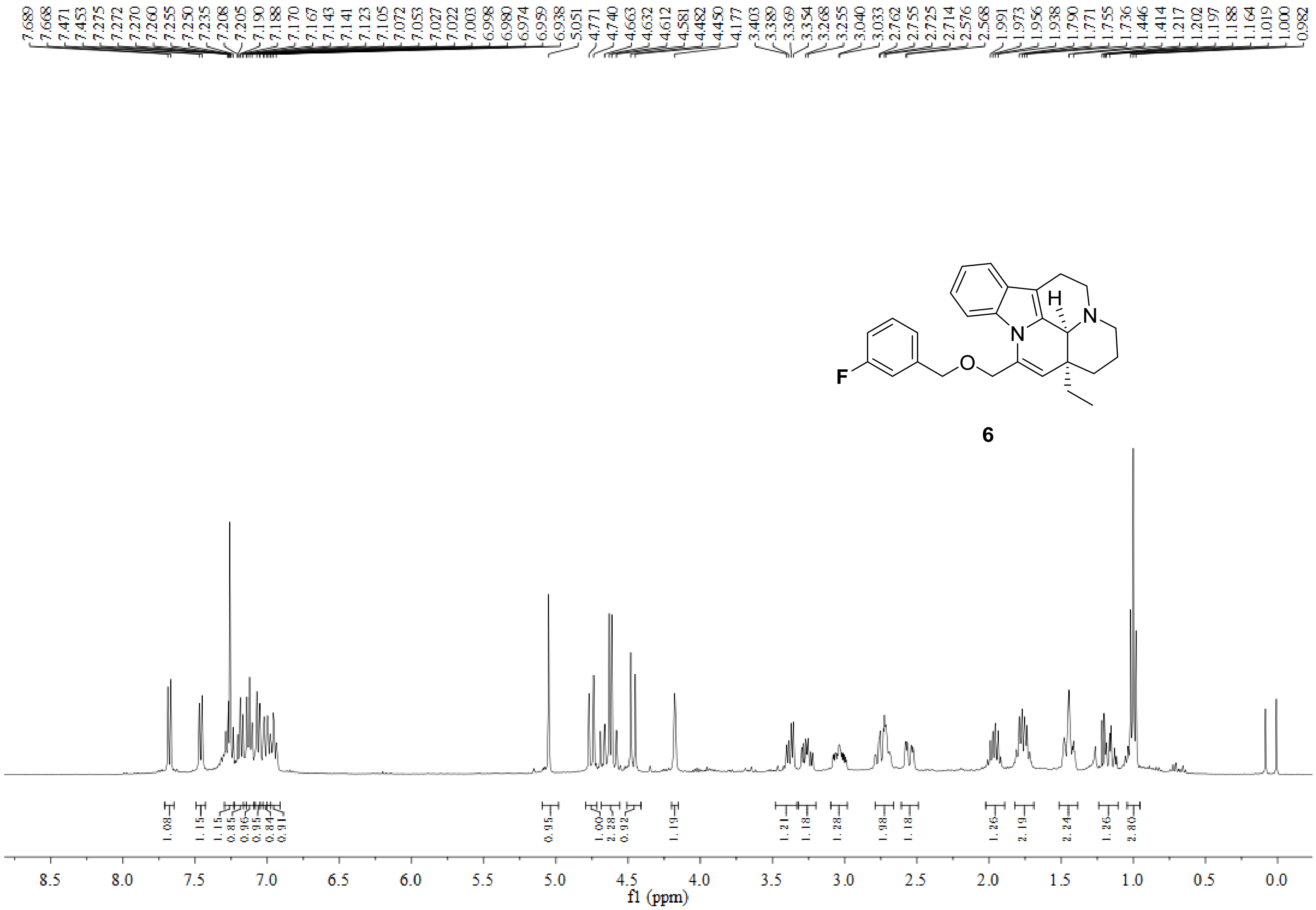

6

^{1}H NMR of Compound **6** (400 MHz, $CDCl_3$)

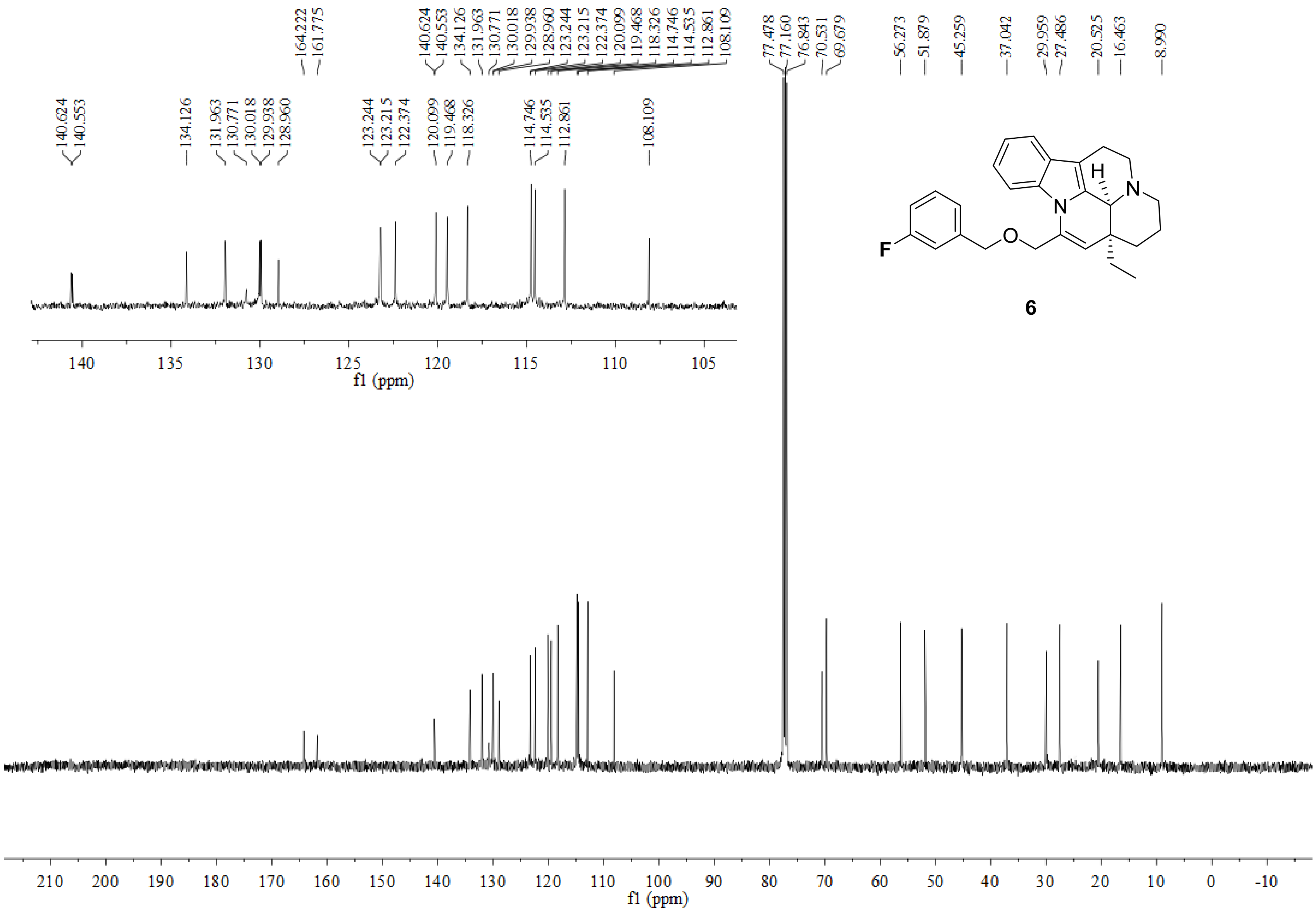

^{13}C NMR of Compound **6** (100 MHz, $CDCl_3$)

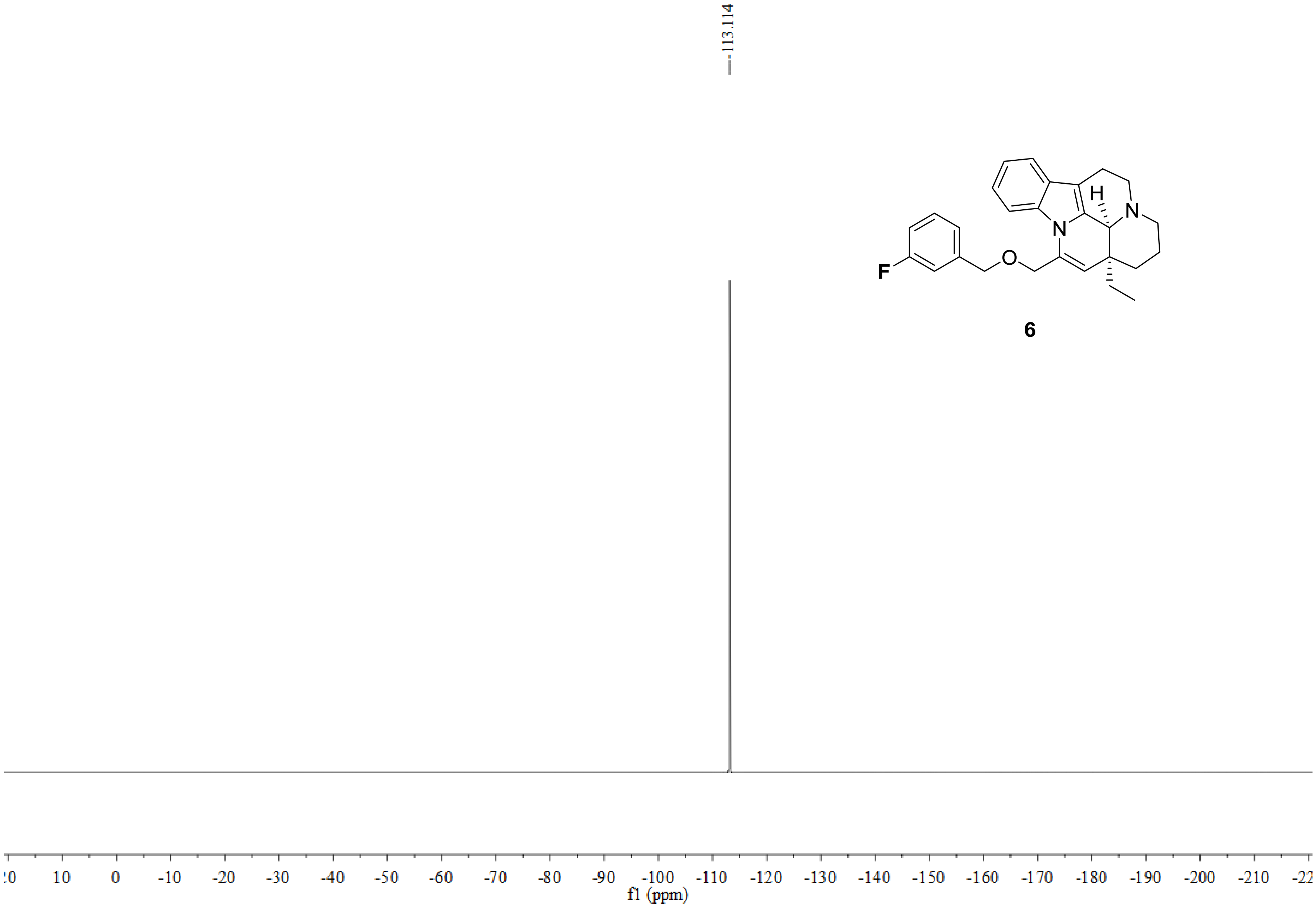

^{19}F NMR of Compound **6** (377 MHz, $CDCl_3$)

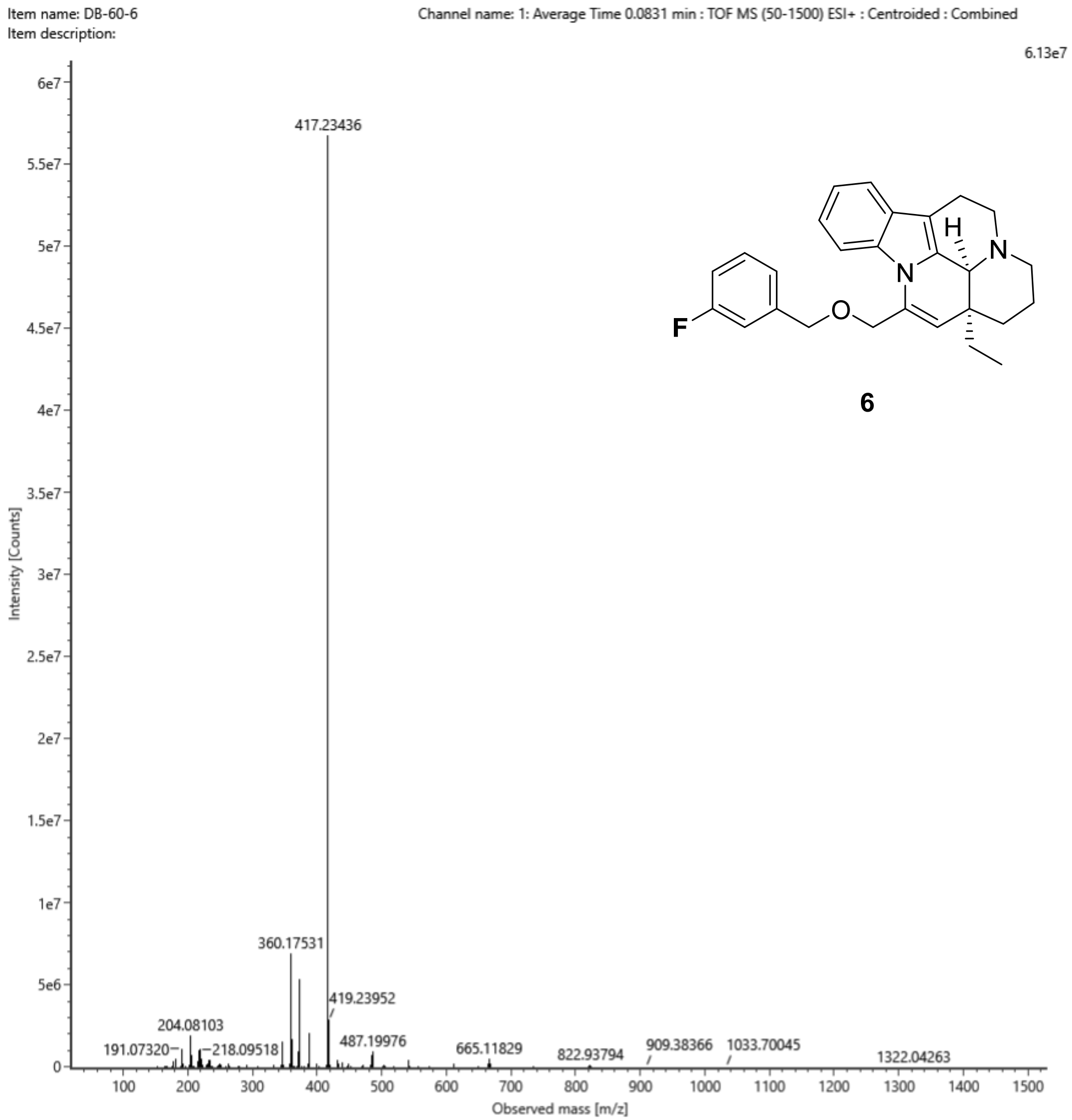

HRMS of Compound **6**

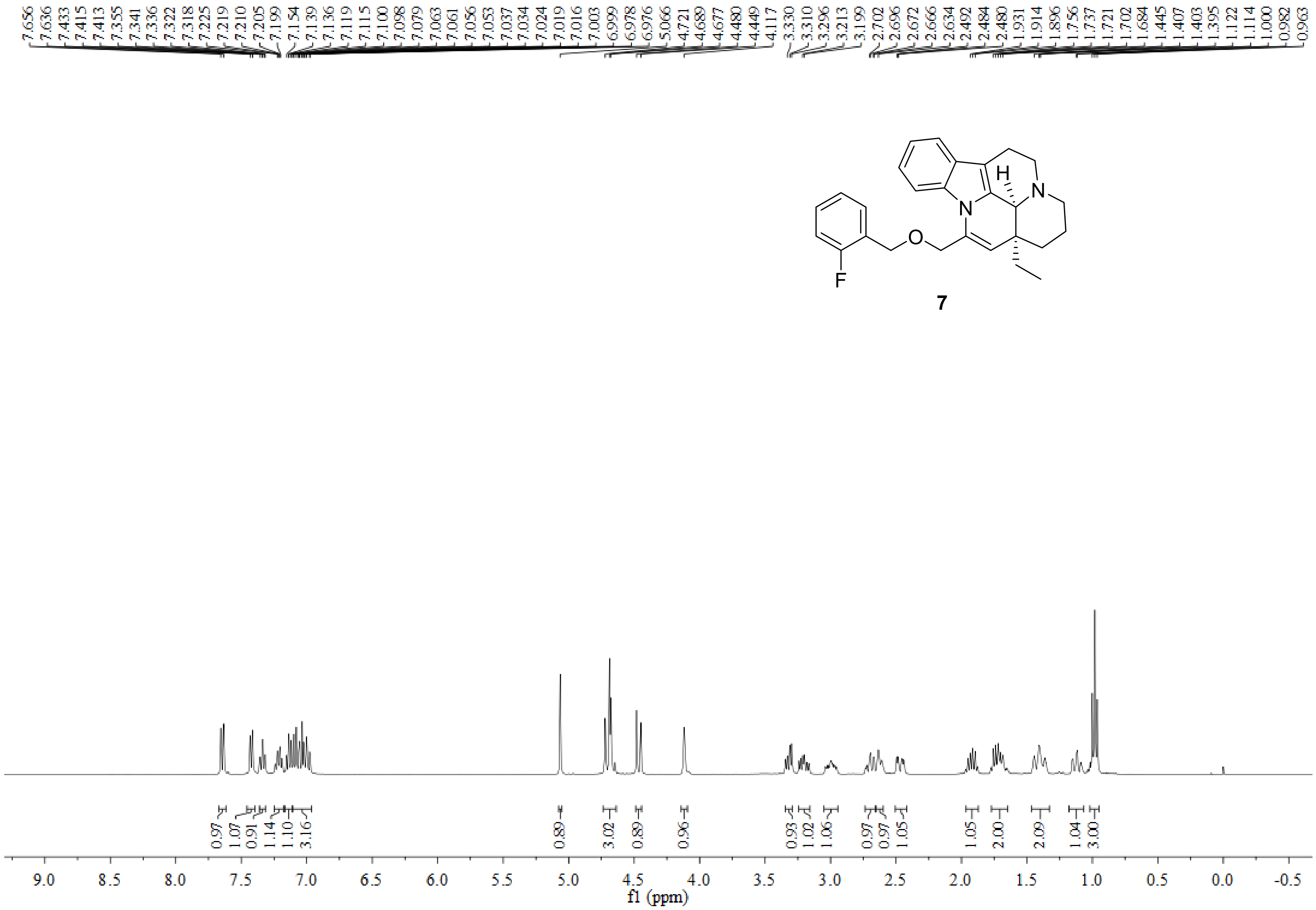

^{1}H NMR of Compound **7** (400 MHz, $CDCl_3$)

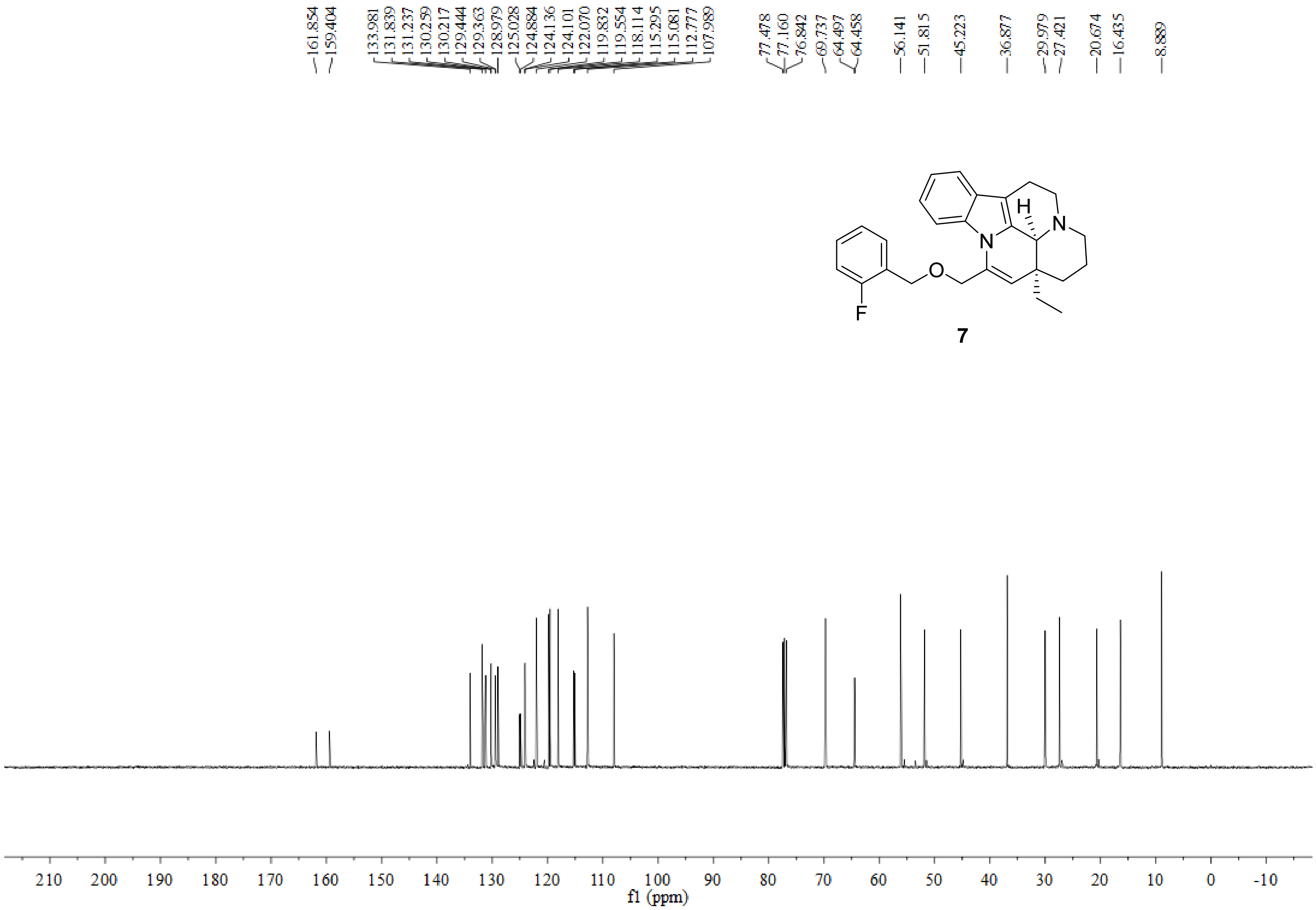

^{13}C NMR of Compound **7** (100 MHz, $CDCl_3$)

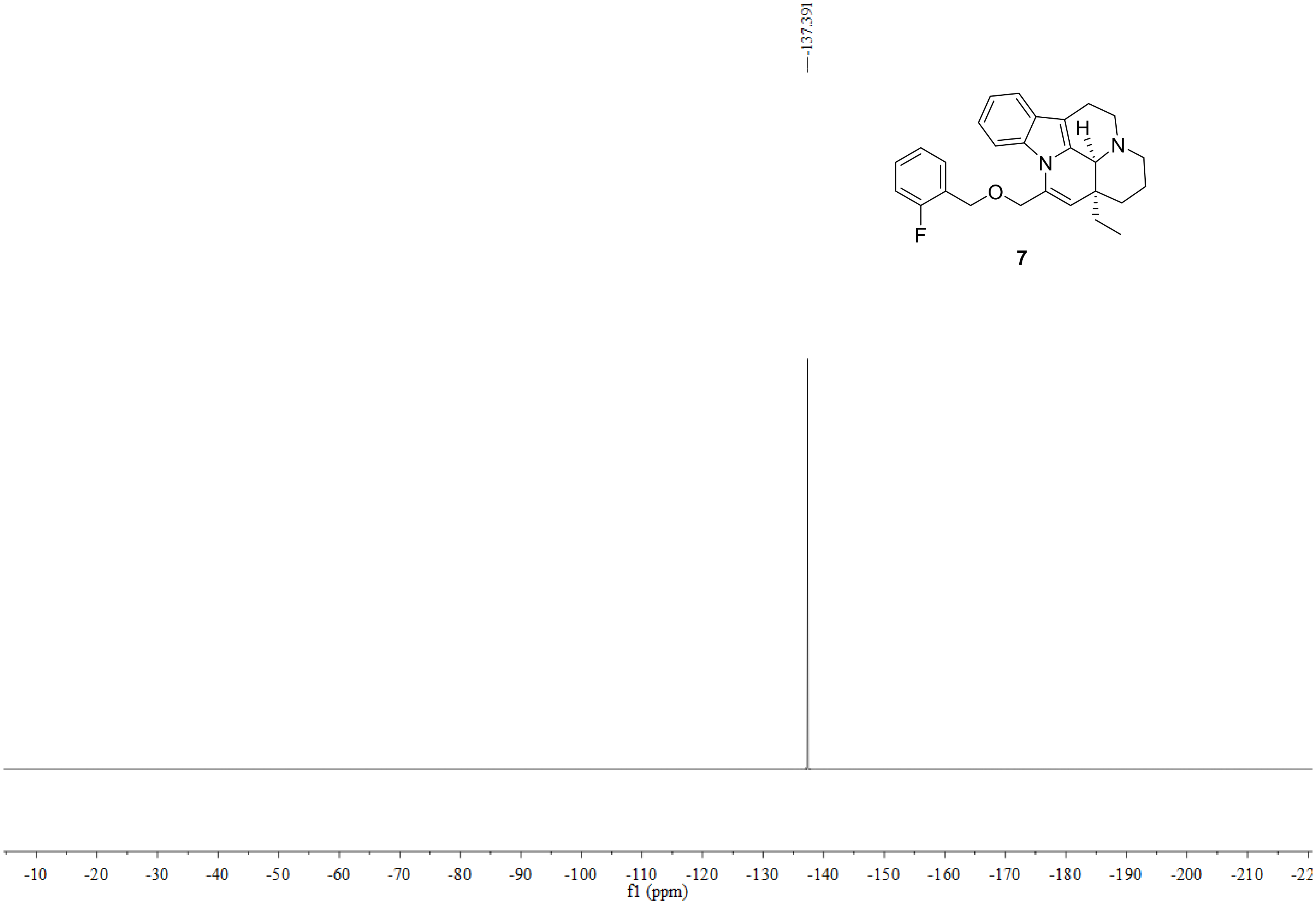

^{19}F NMR of Compound **7** (377 MHz, $CDCl_3$)

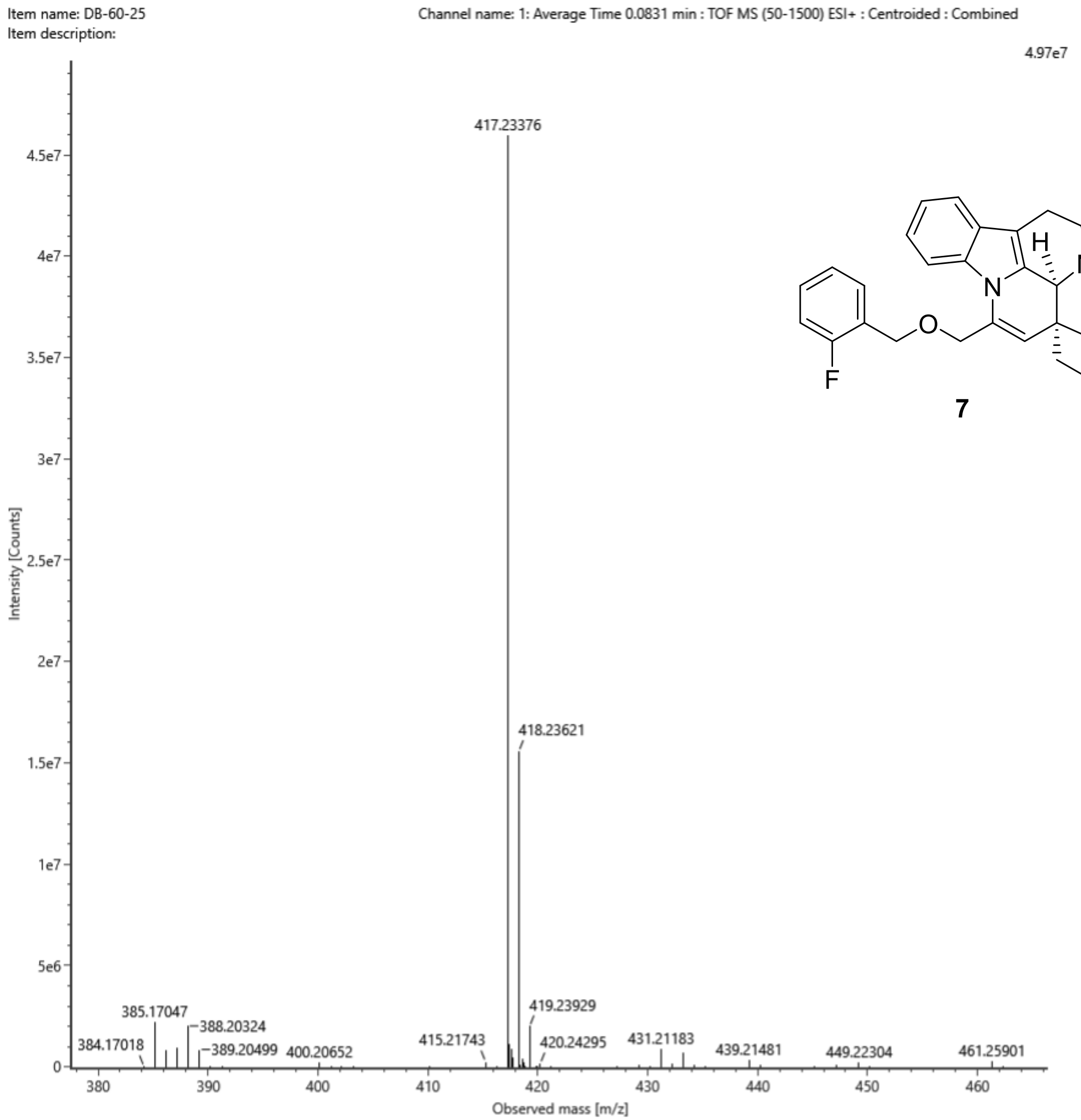

HRMS of Compound **7**

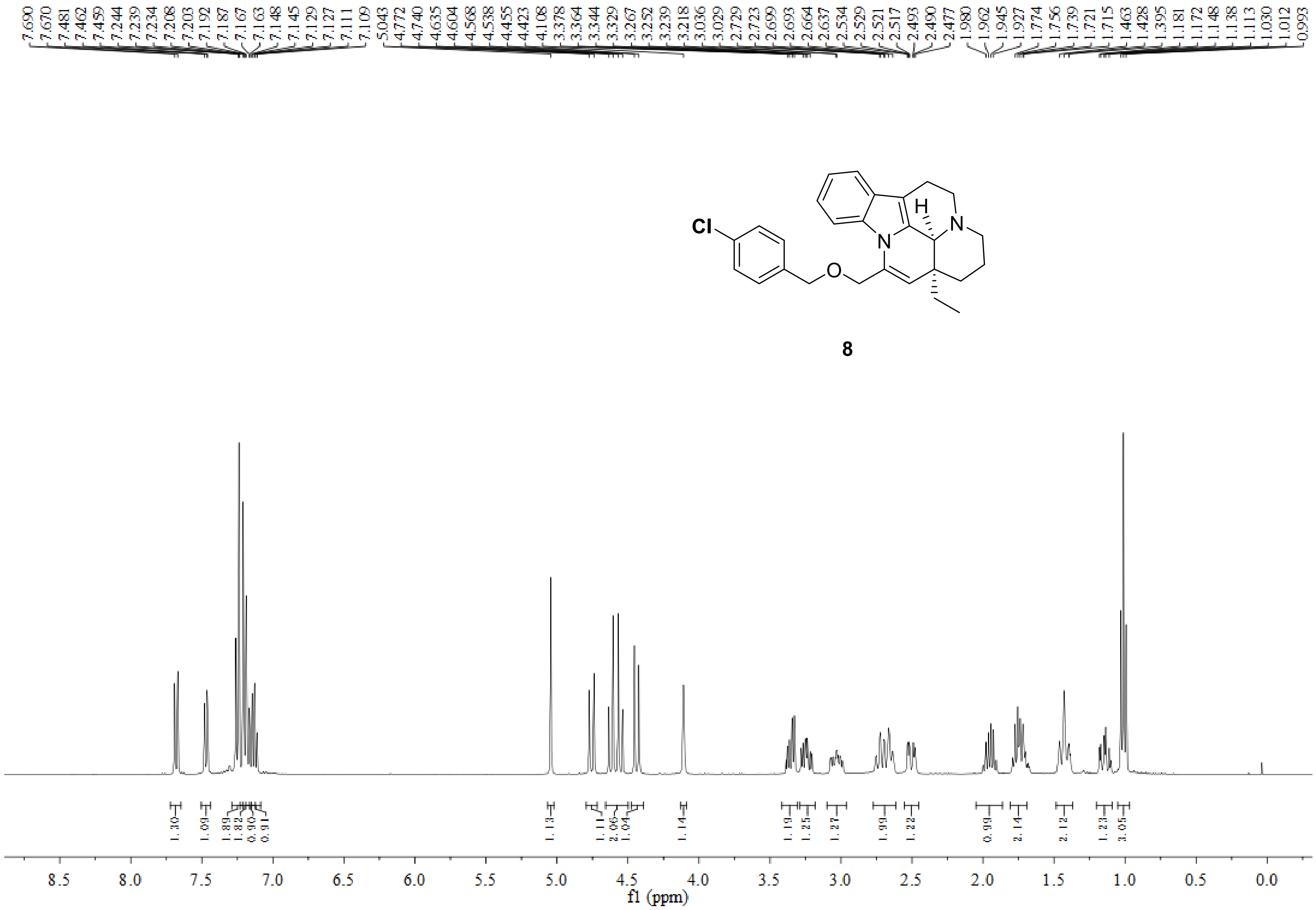

^{1}H NMR of Compound **8** (400 MHz, $CDCl_3$)

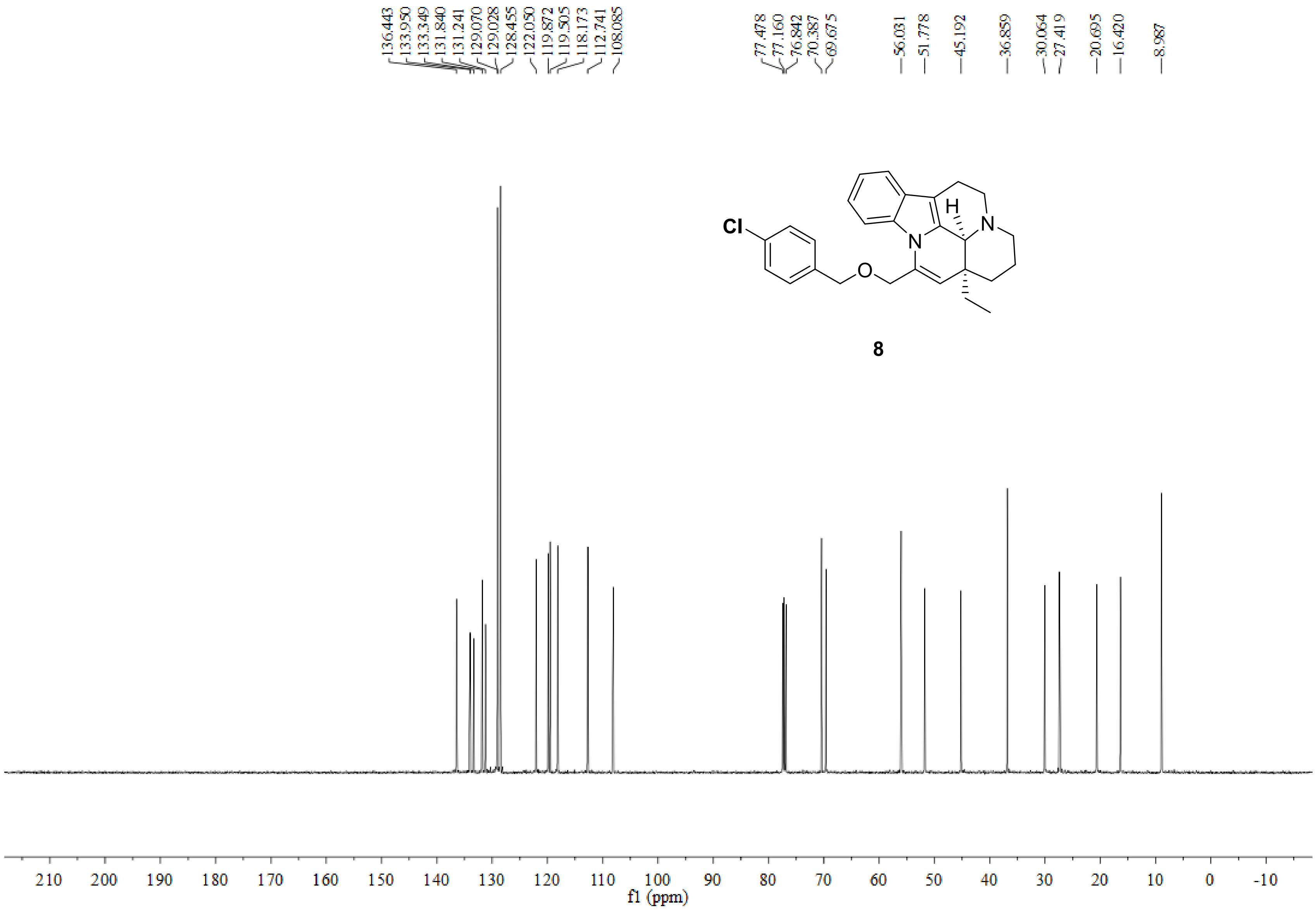

^{13}C NMR of Compound 8 (100 MHz, $CDCl_3$)

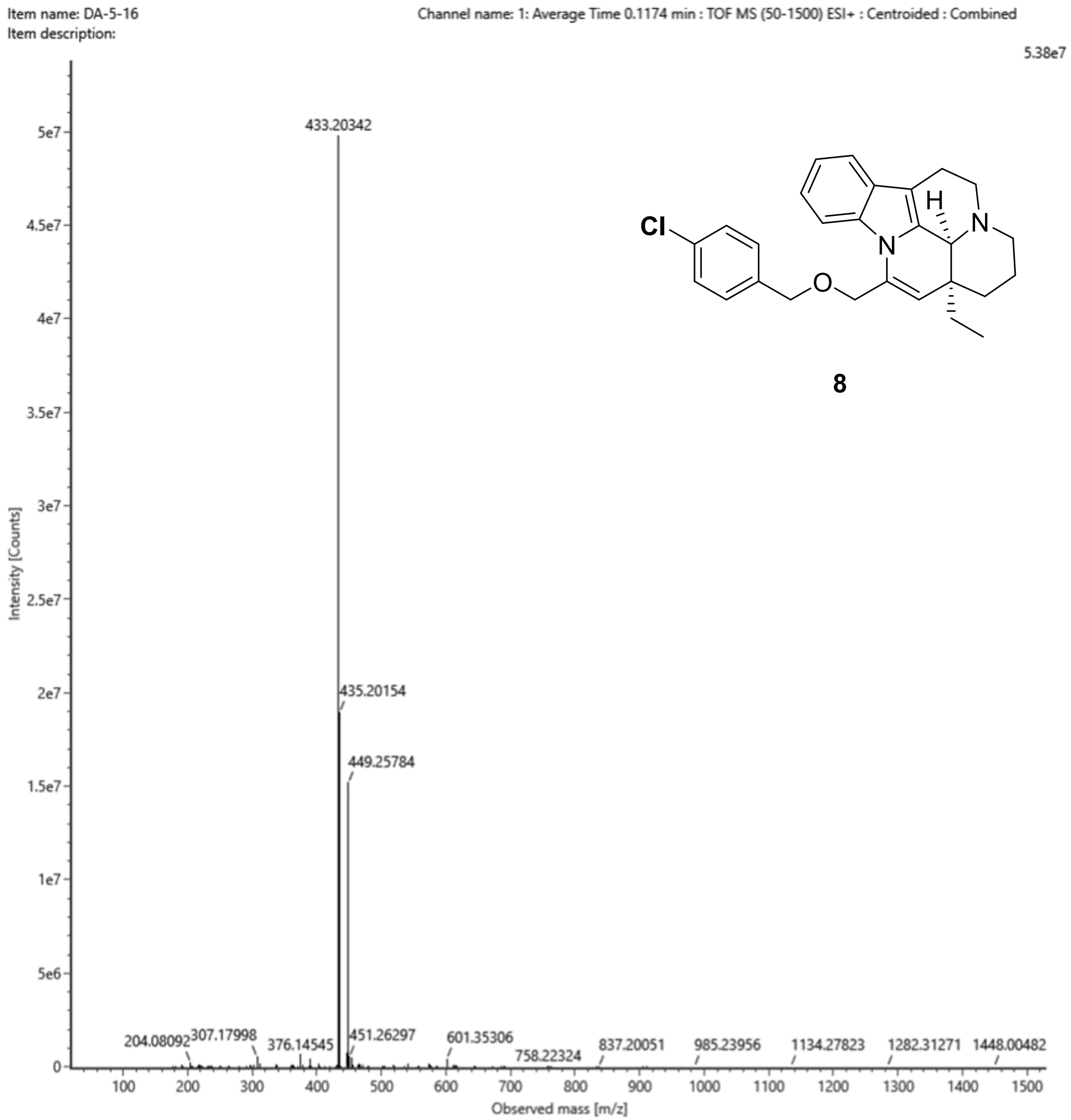

HRMS of Compound **8**

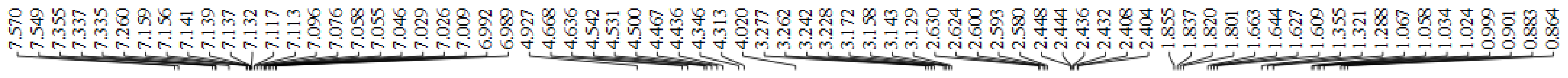

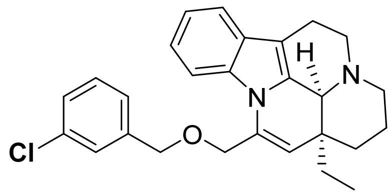

9

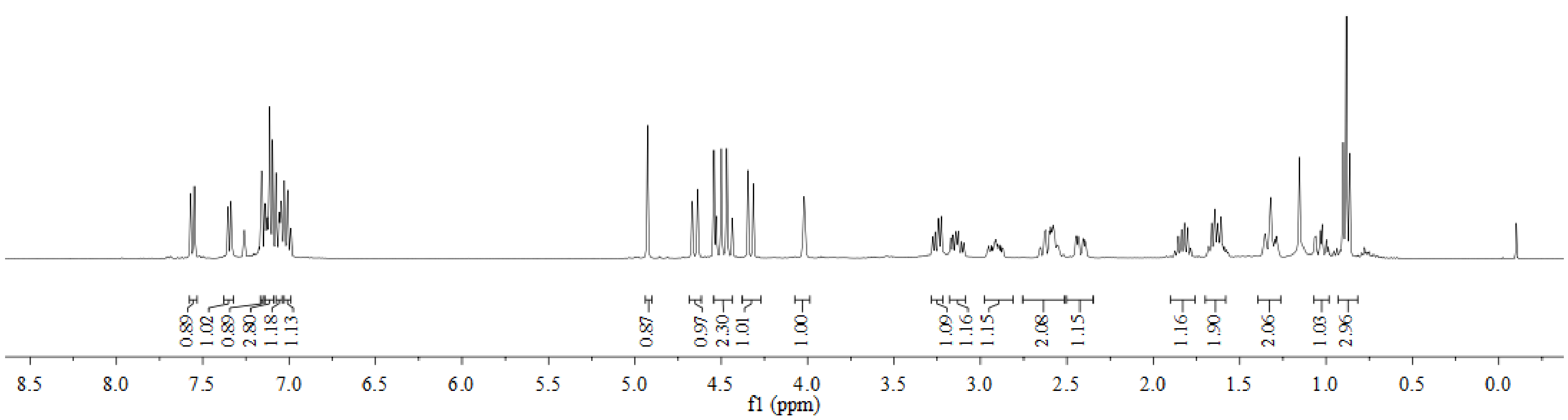

^{1}H NMR of Compound **9** (400 MHz, $CDCl_3$)

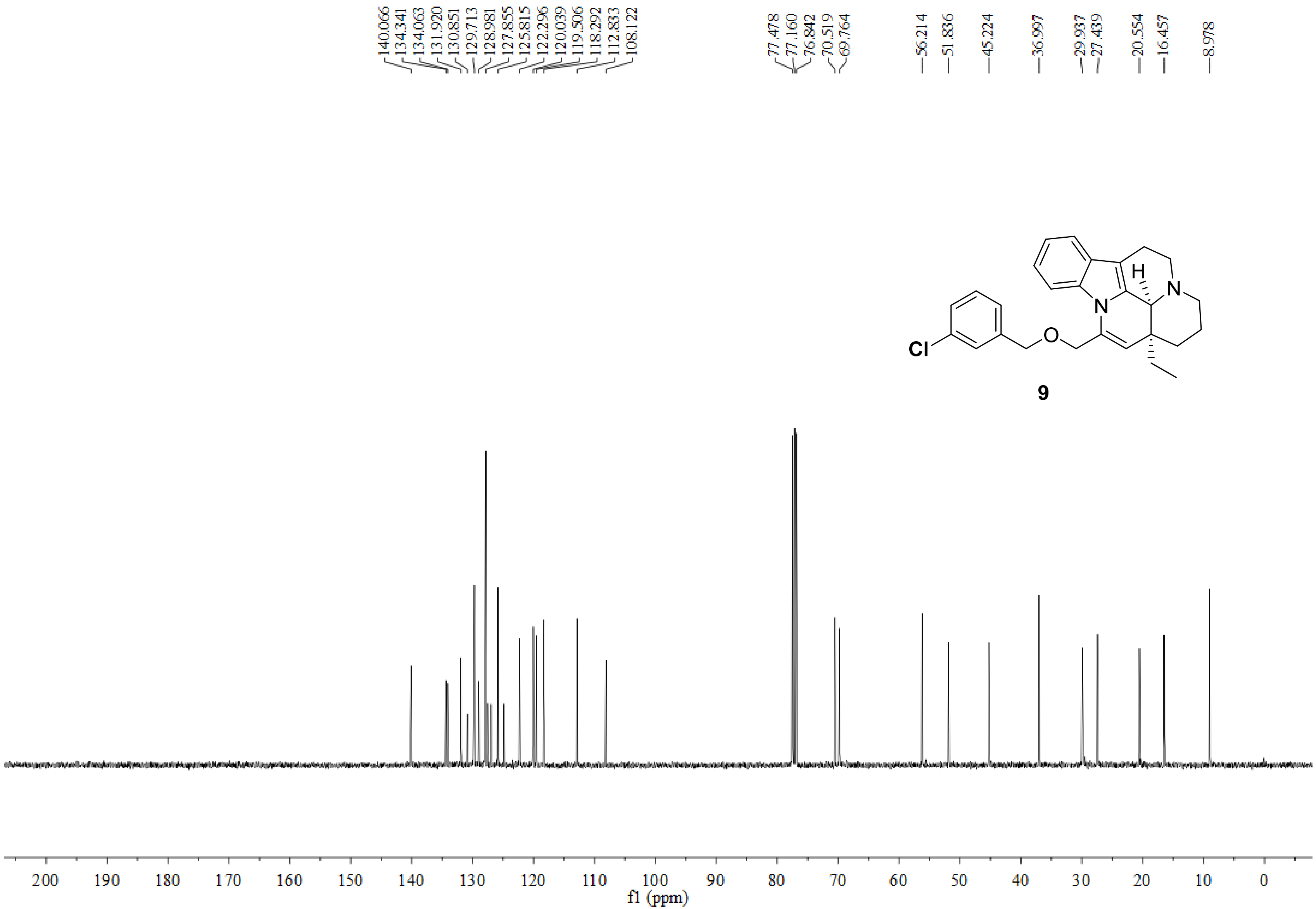

^{13}C NMR of Compound **9** (100 MHz, $CDCl_3$)

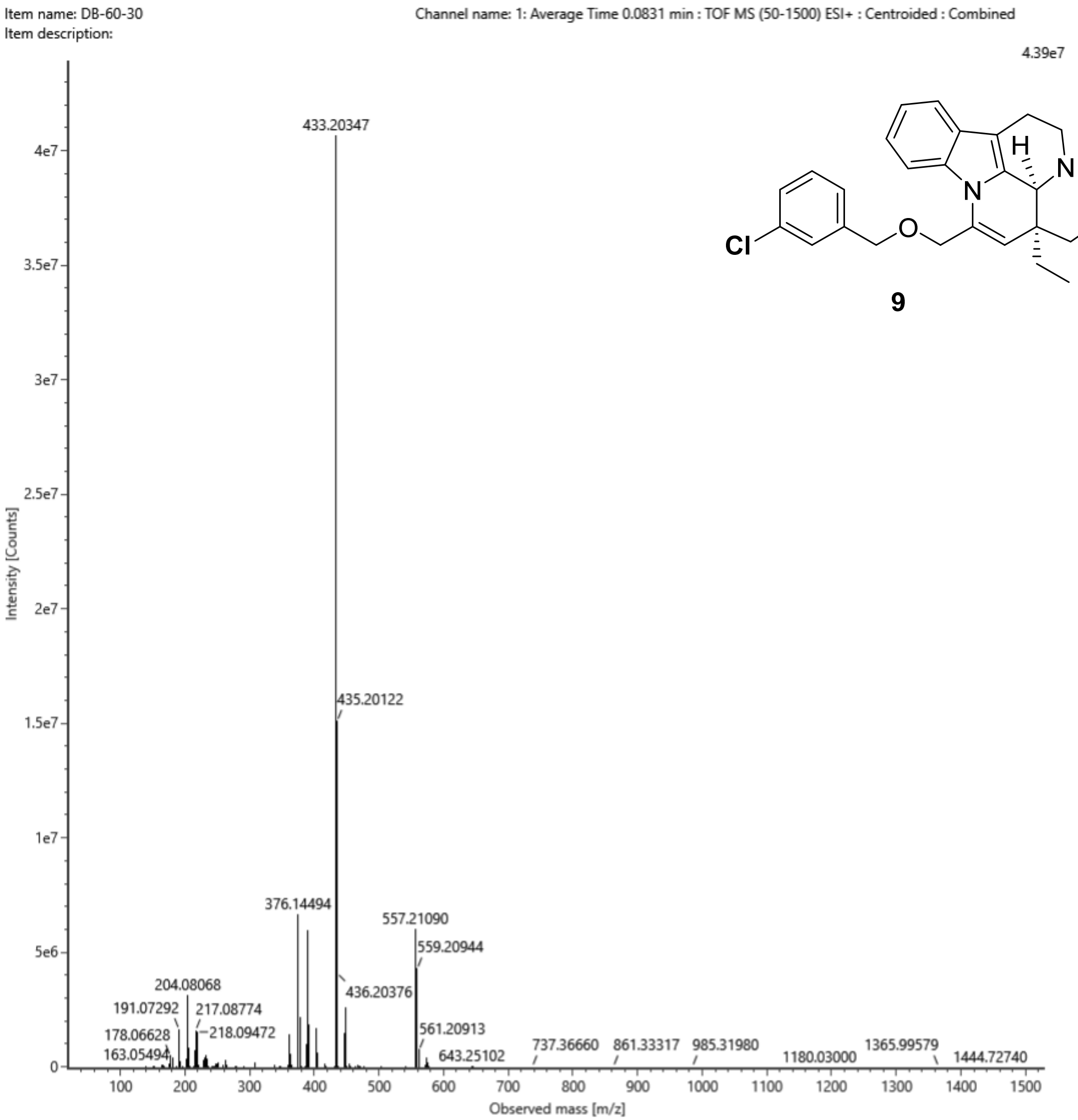

HRMS of Compound **9**

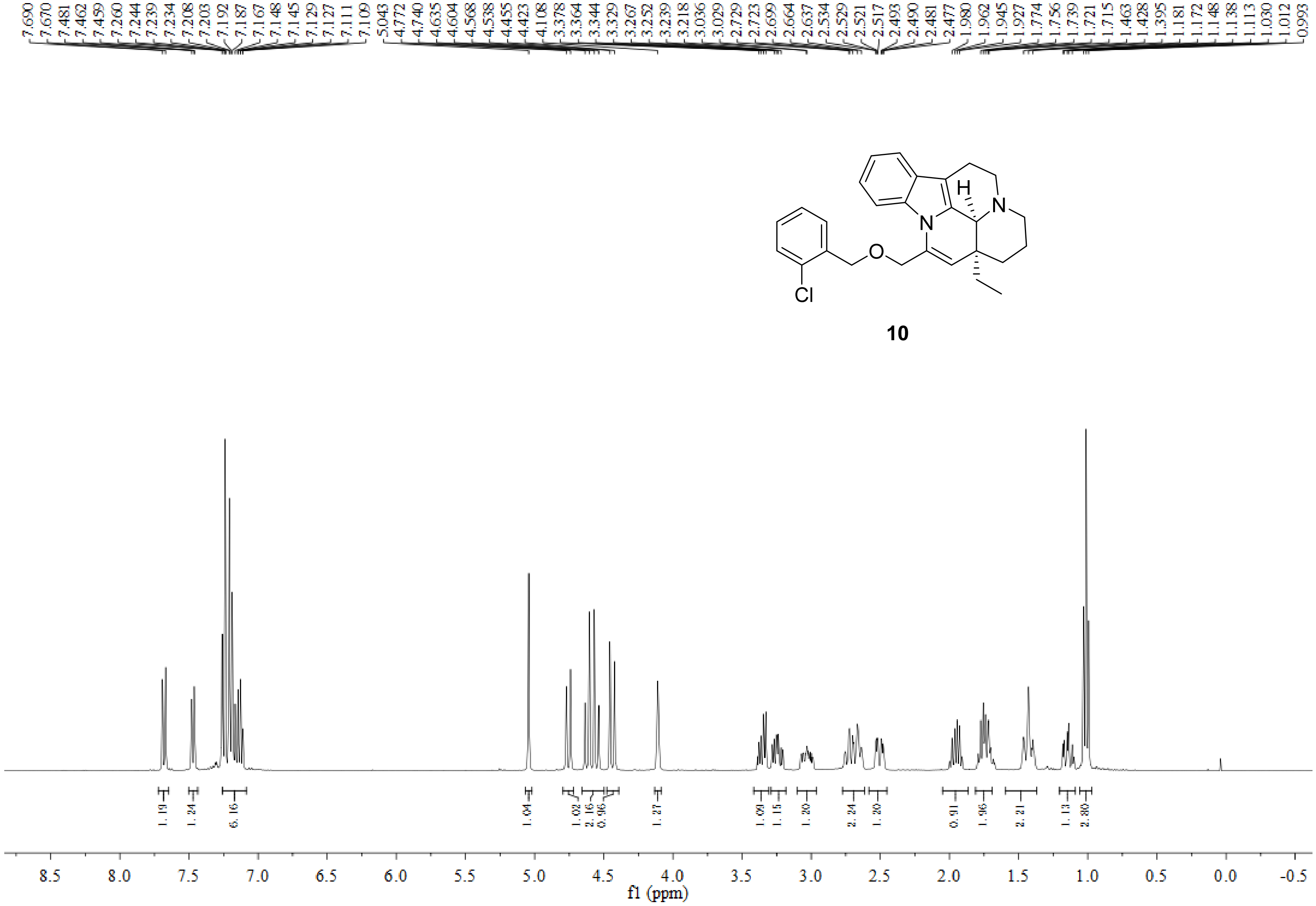

^{1}H NMR of Compound **10** (400 MHz, $CDCl_3$)

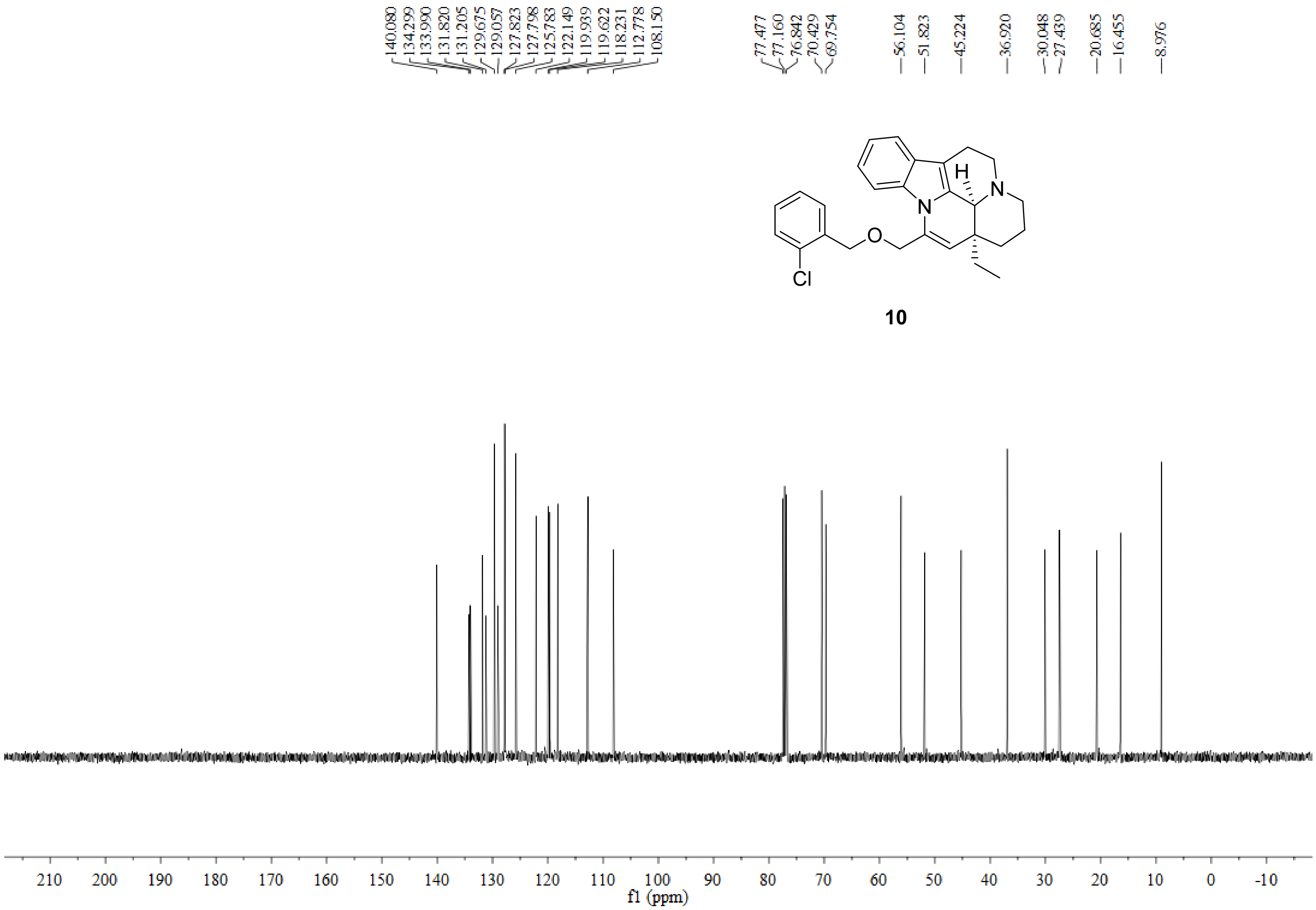

^{13}C NMR of Compound **10** (100 MHz, $CDCl_3$)

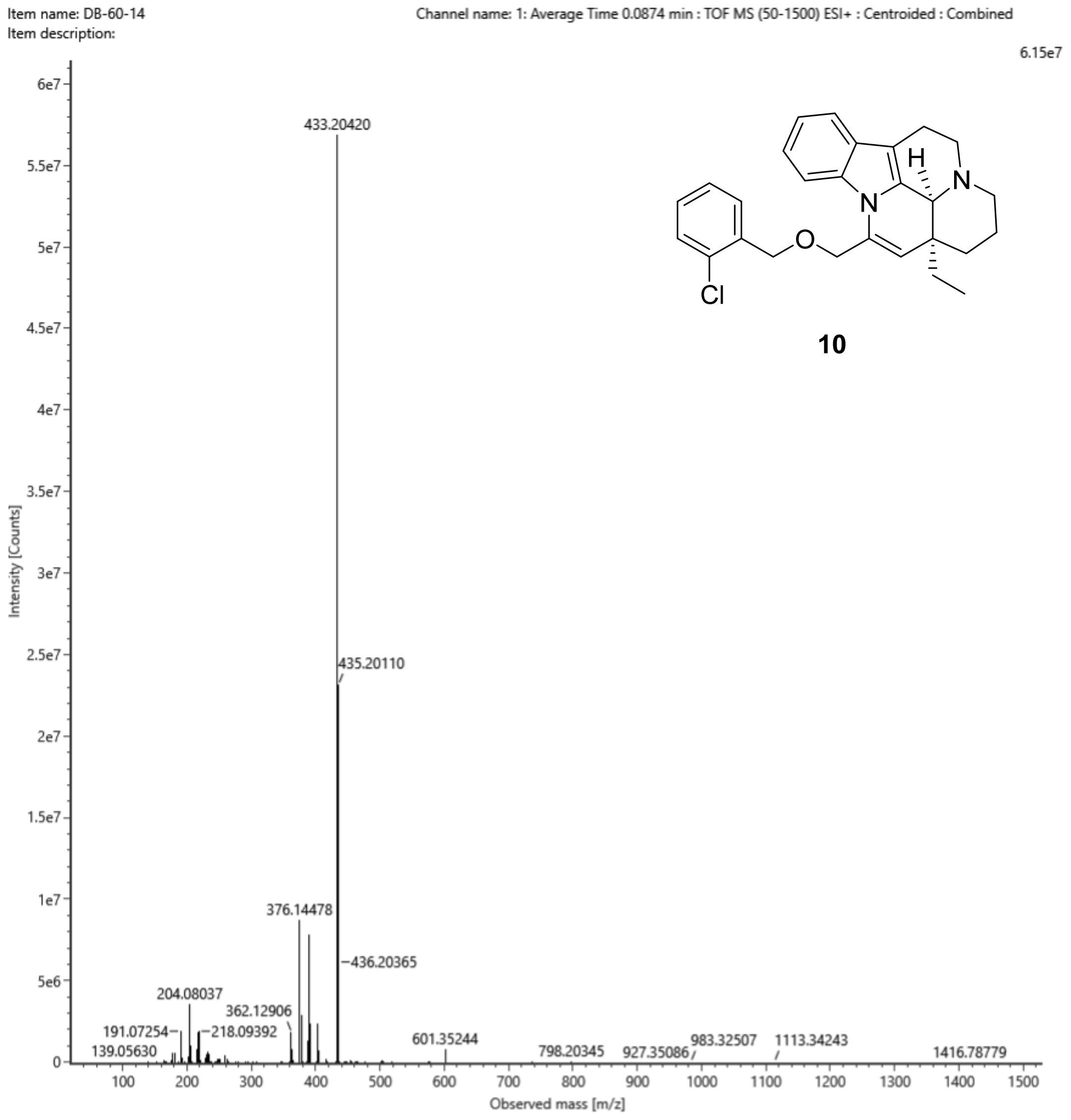

HRMS of Compound **10**

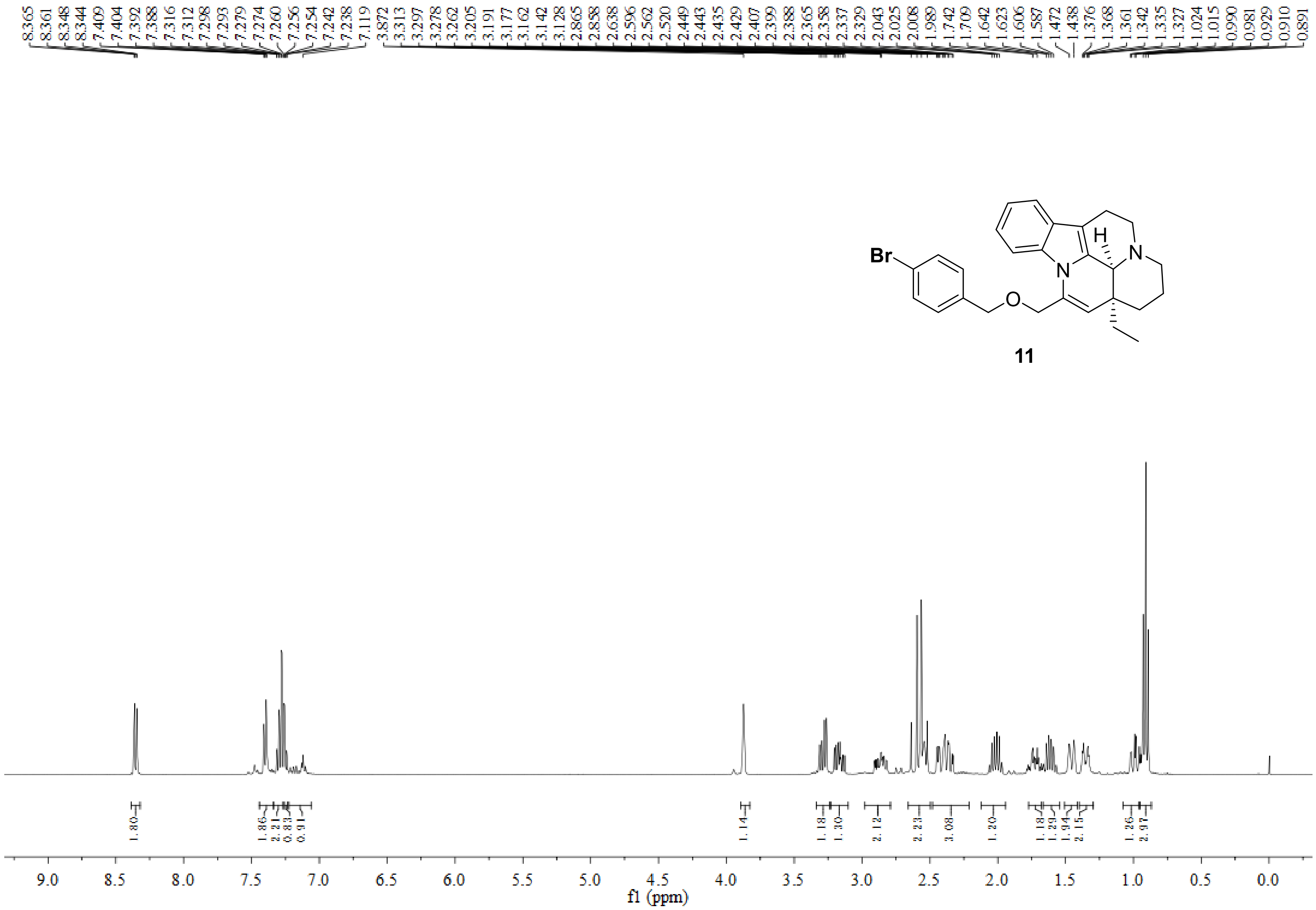

^{1}H NMR of Compound **11** (400 MHz, $CDCl_3$)

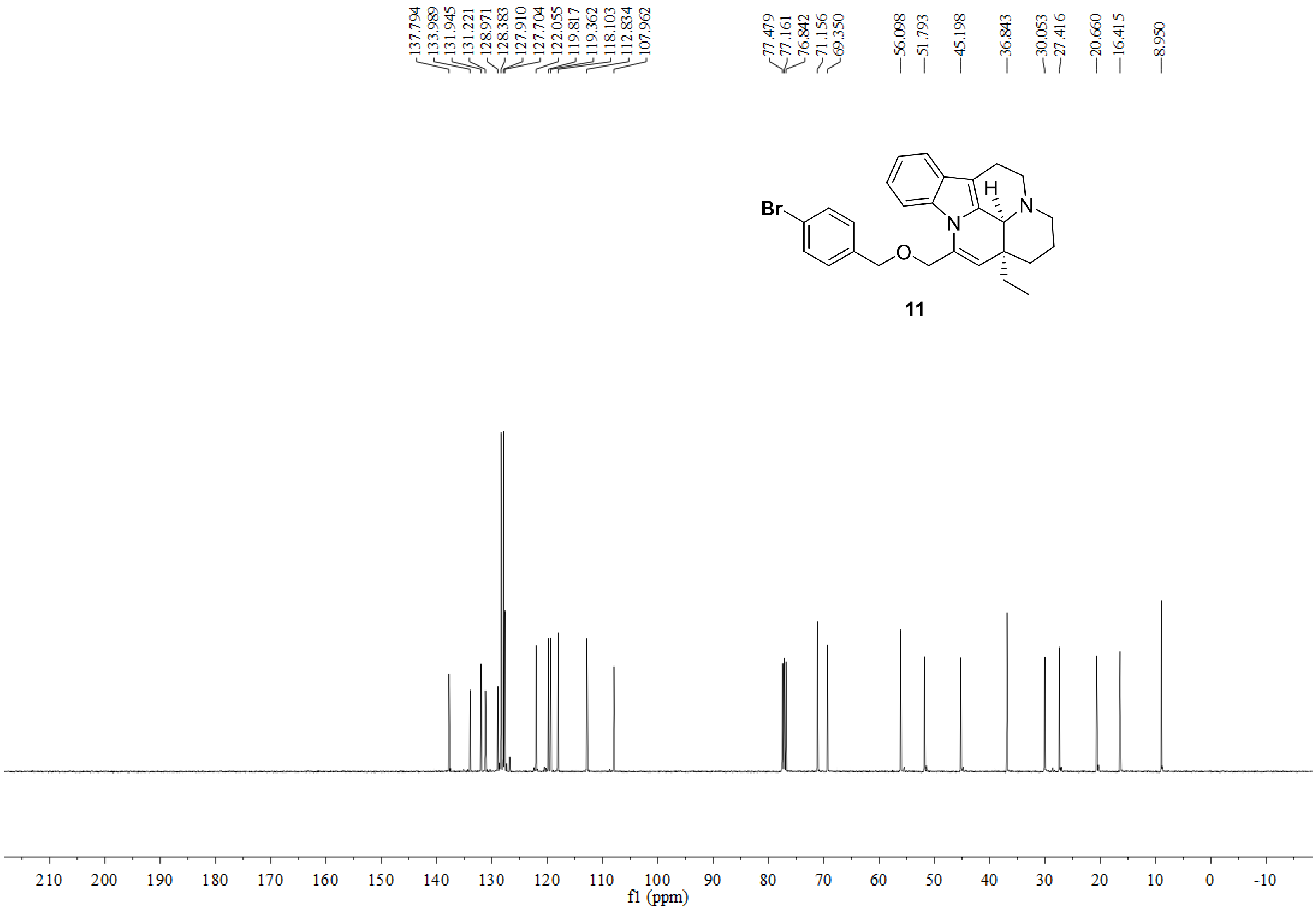

^{13}C NMR of Compound **11** (100 MHz, $CDCl_3$)

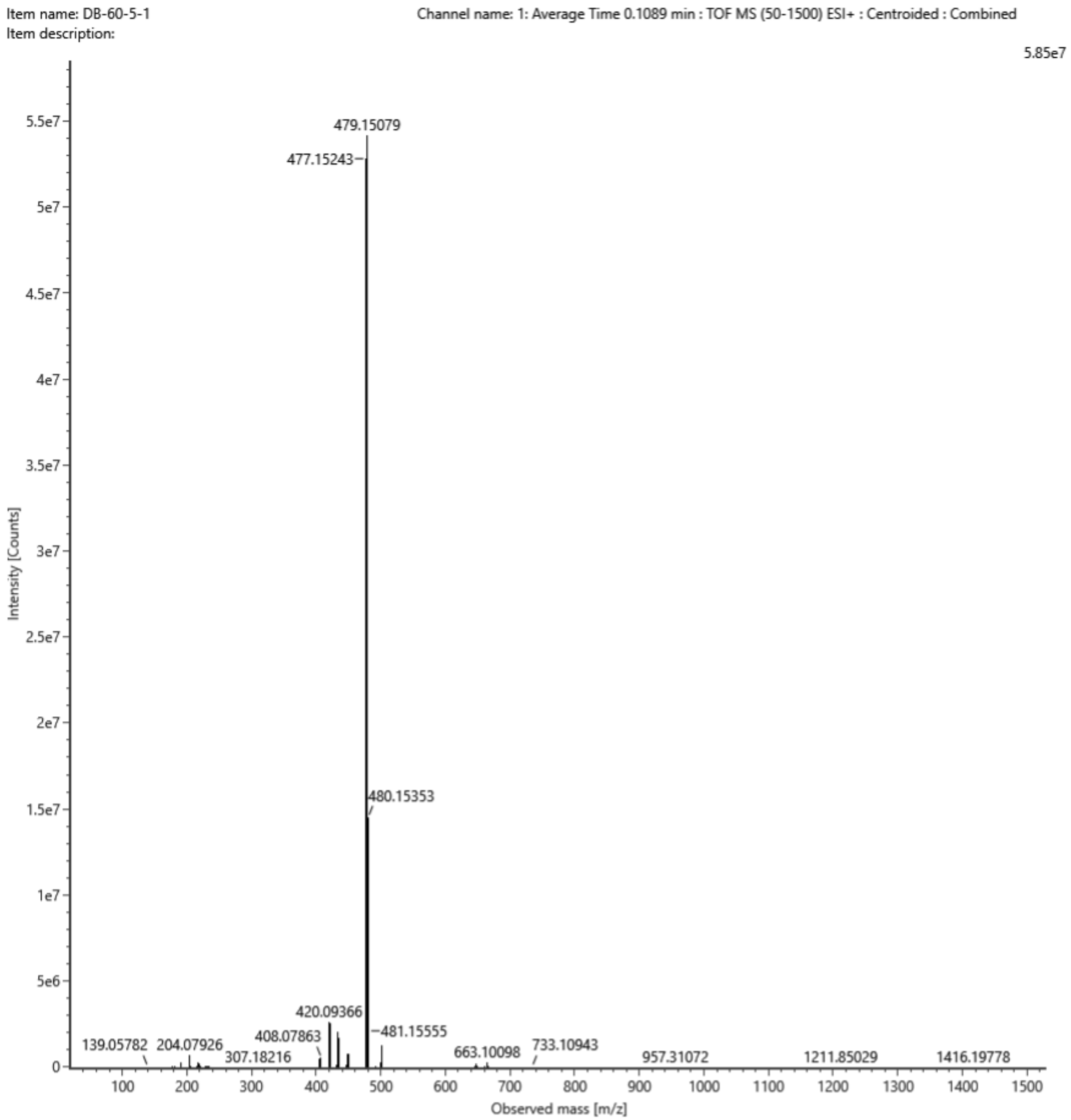

HRMS of Compound **11**

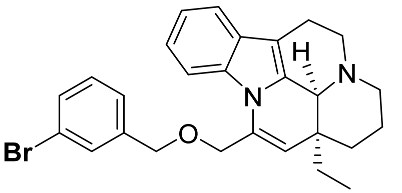

12

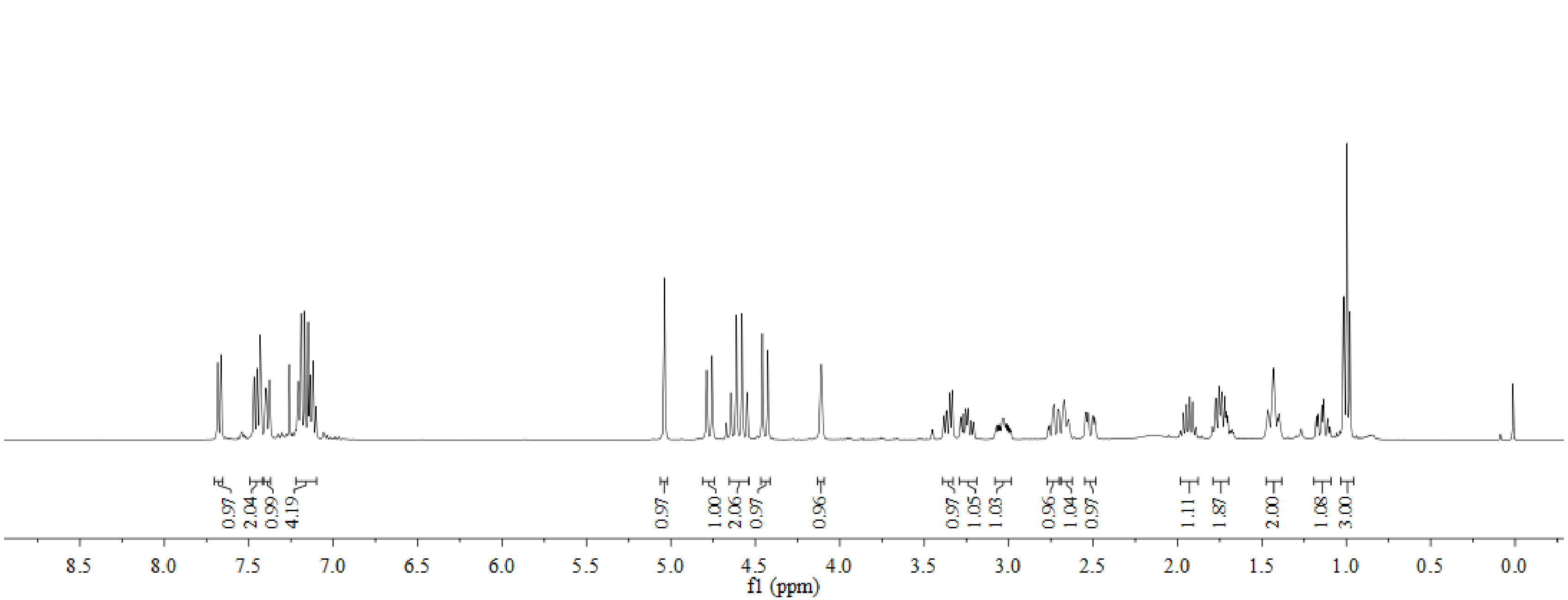

^{1}H NMR of Compound **12** (400 MHz, $CDCl_3$)

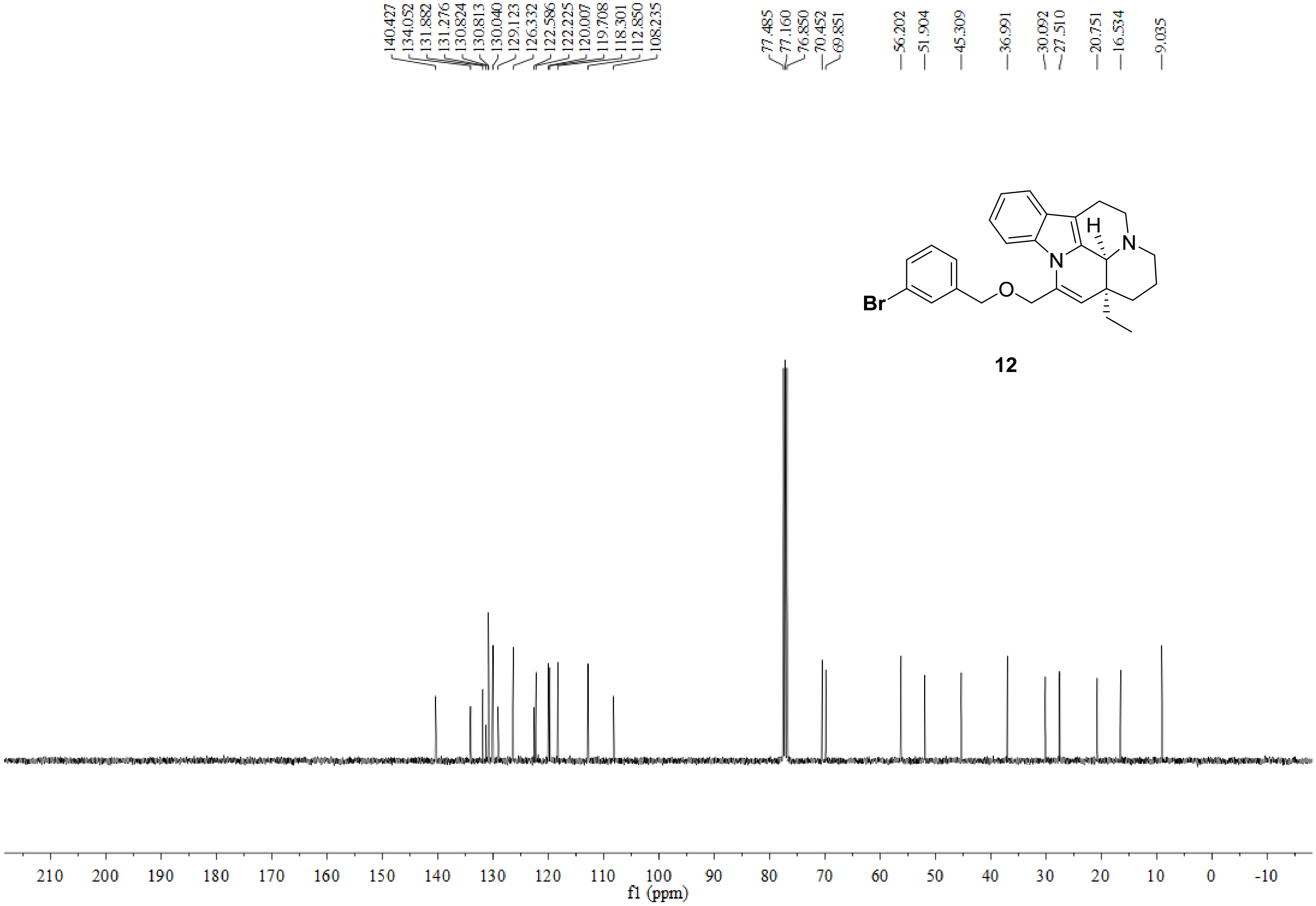

^{13}C NMR of Compound **12** (100 MHz, $CDCl_3$)

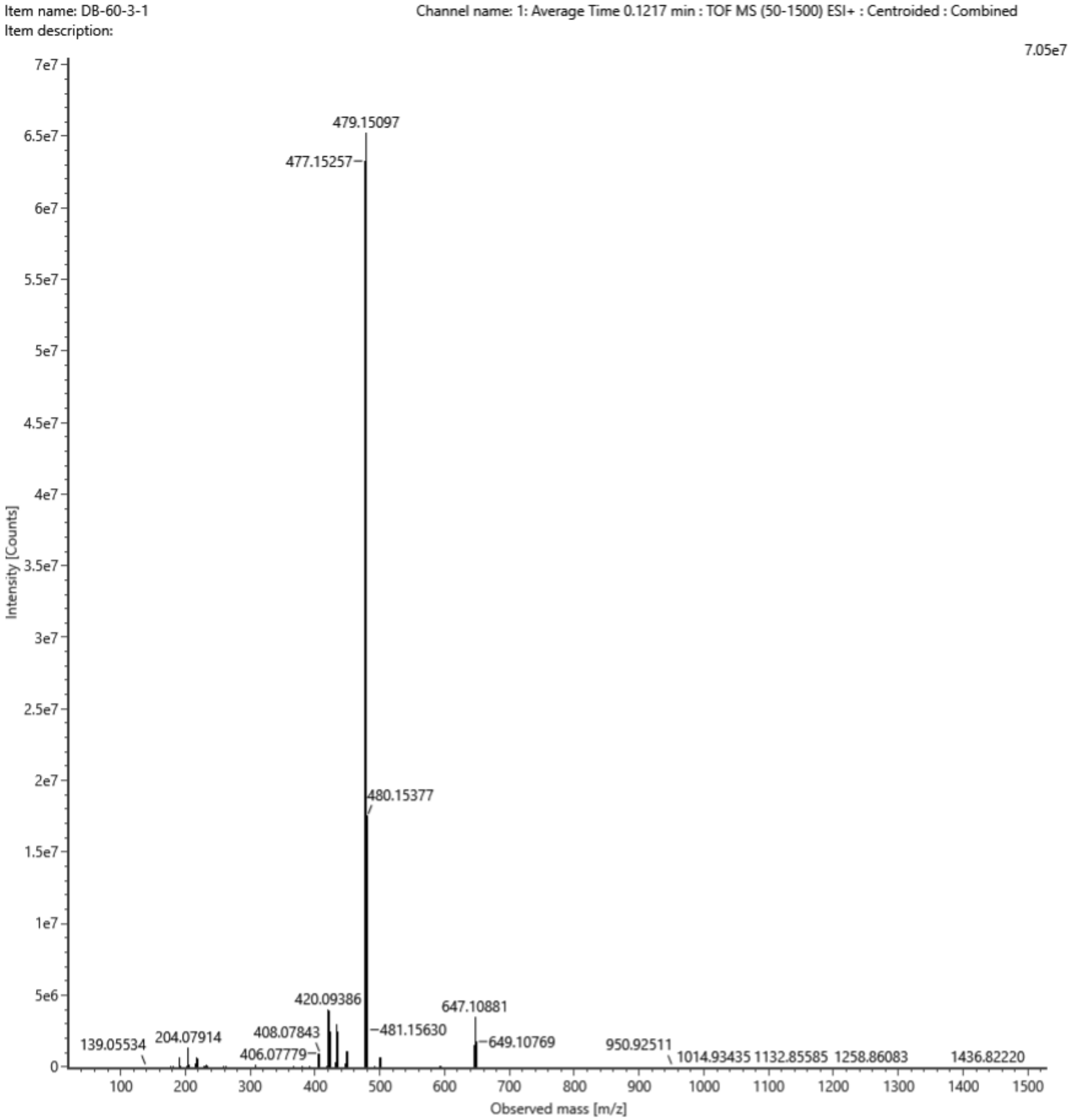

HRMS of Compound **12**

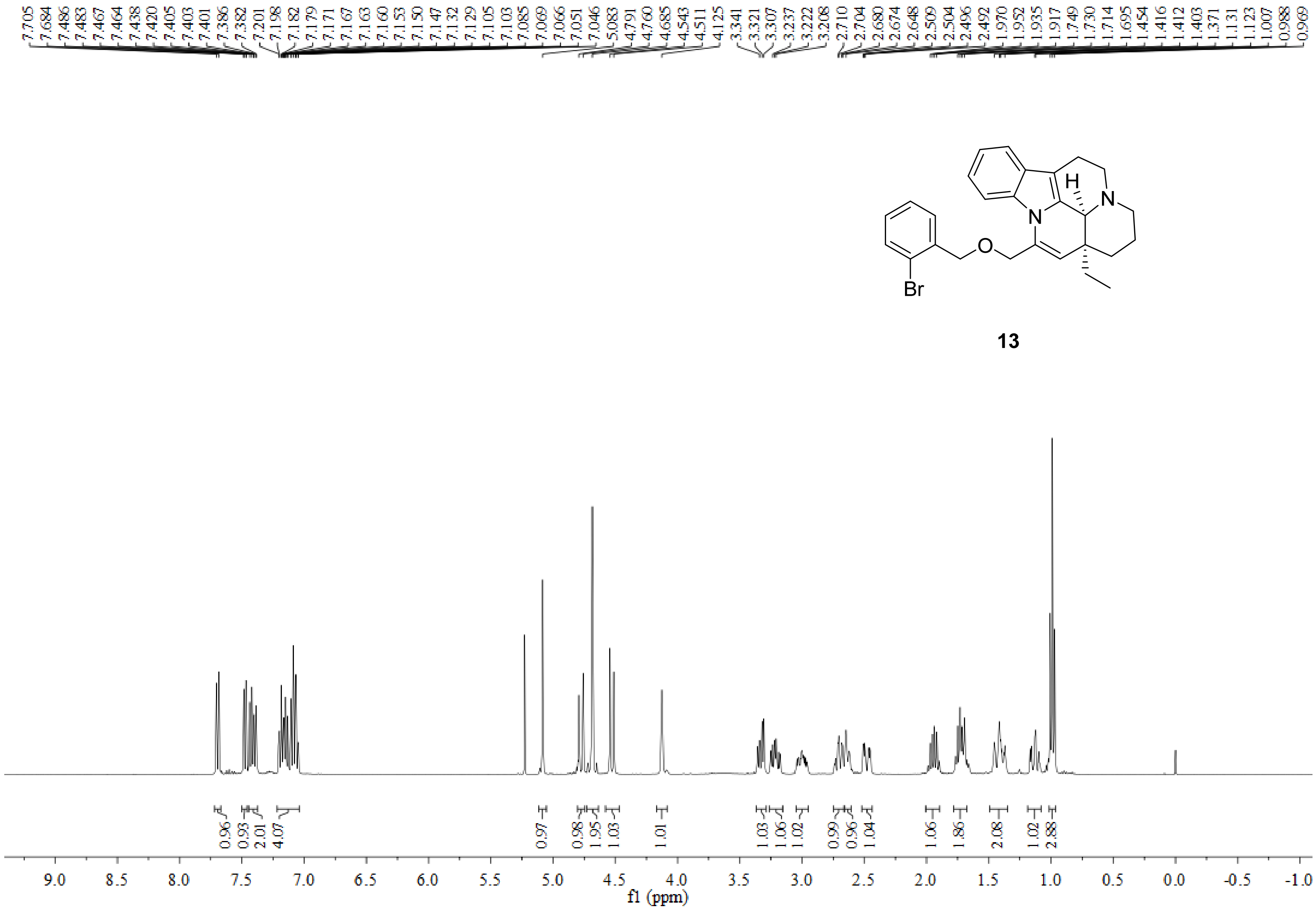

13

^{1}H NMR of Compound **13** (400 MHz, $CDCl_3$)

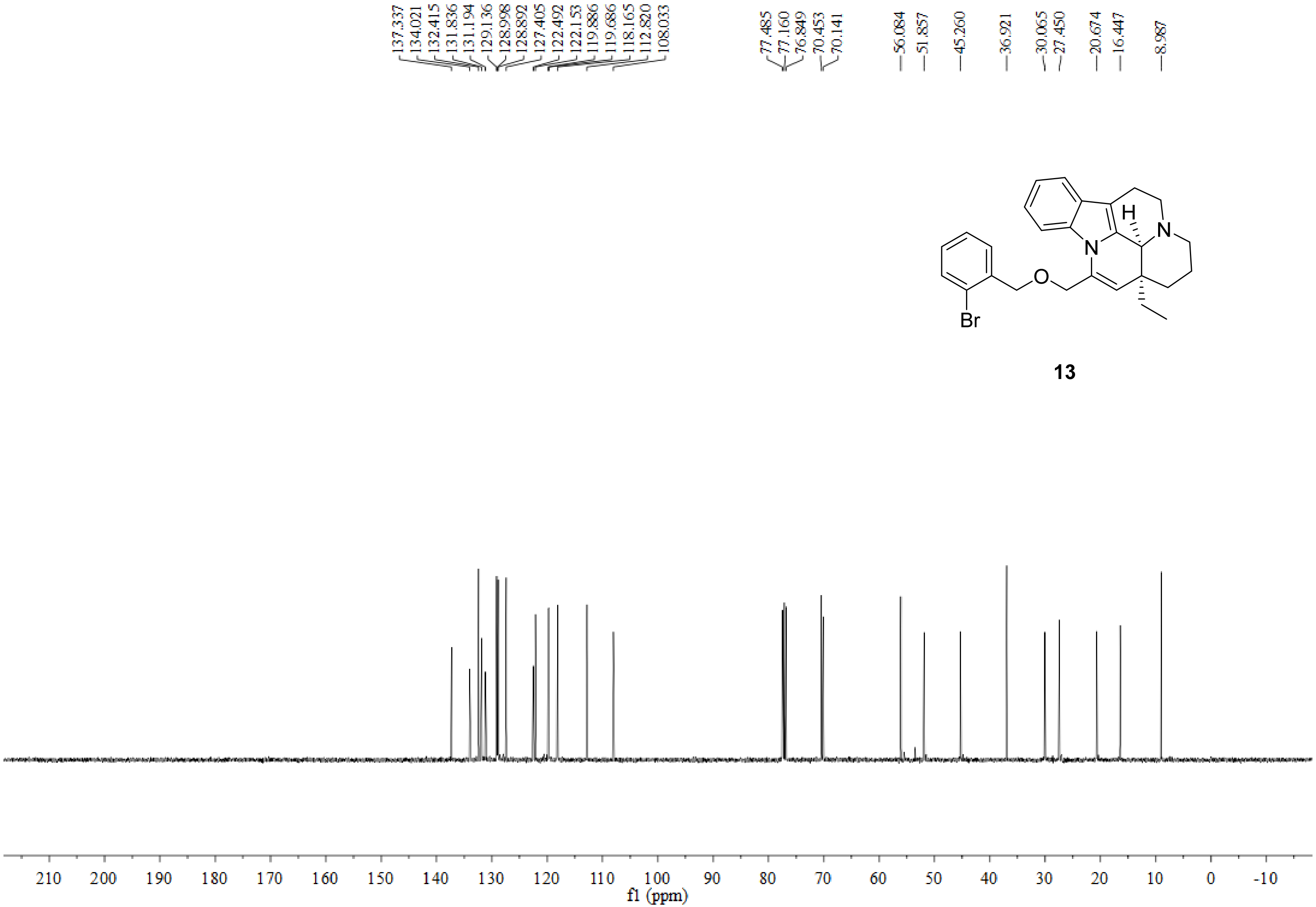

13

^{13}C NMR of Compound **13** (100 MHz, $CDCl_3$)

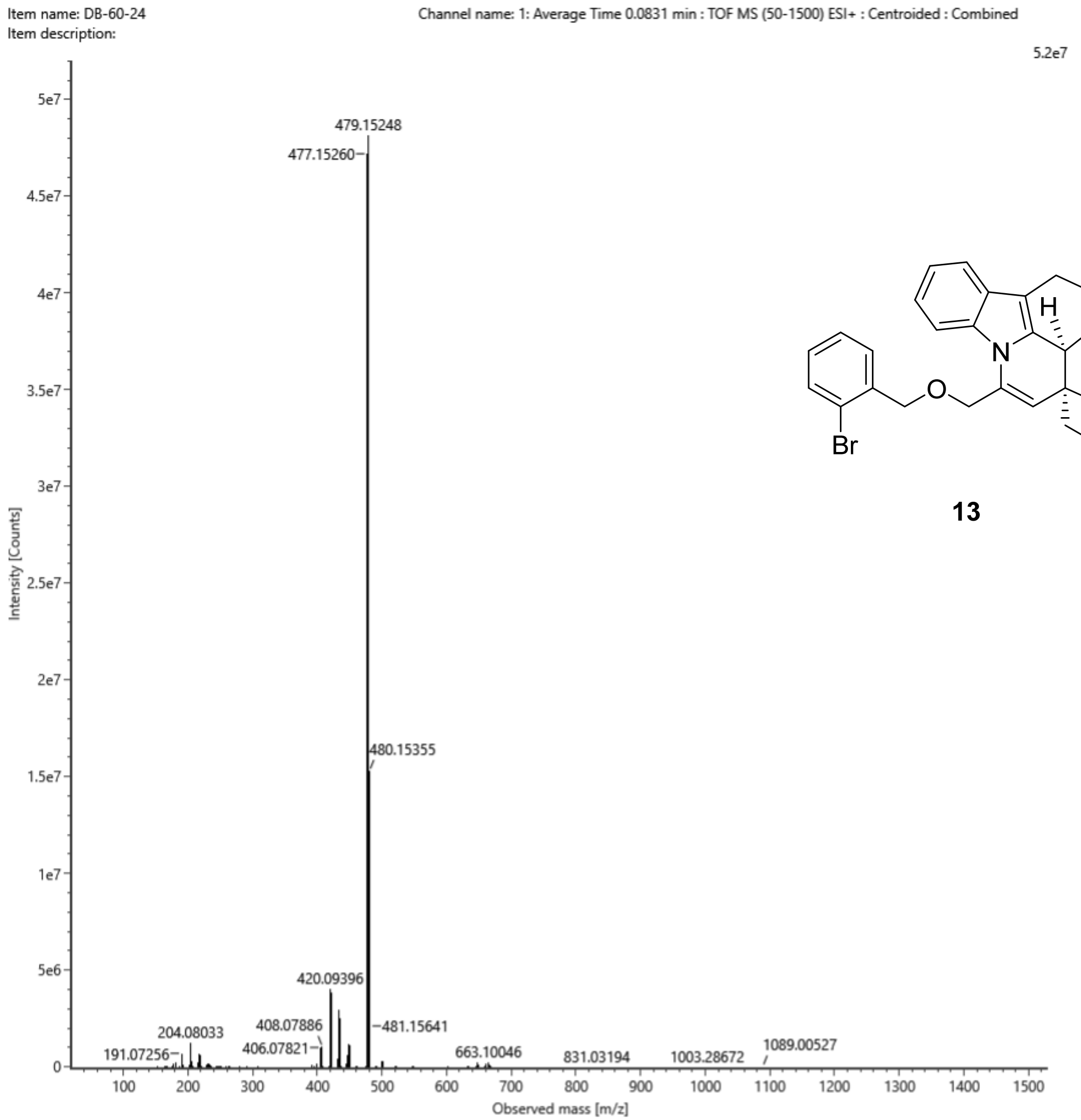

13

HRMS of Compound **13**

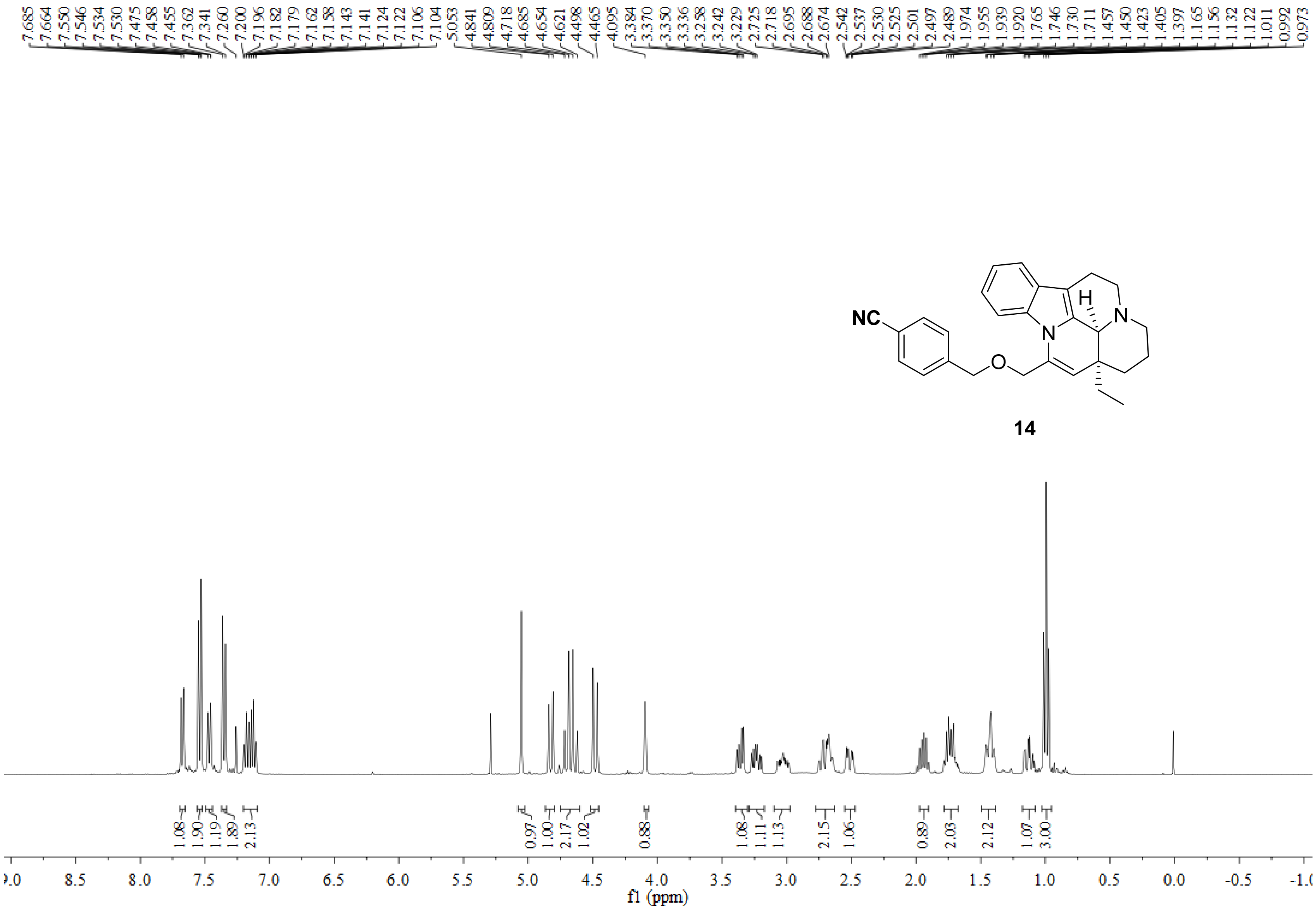

^{1}H NMR of Compound **14** (400 MHz, $CDCl_3$)

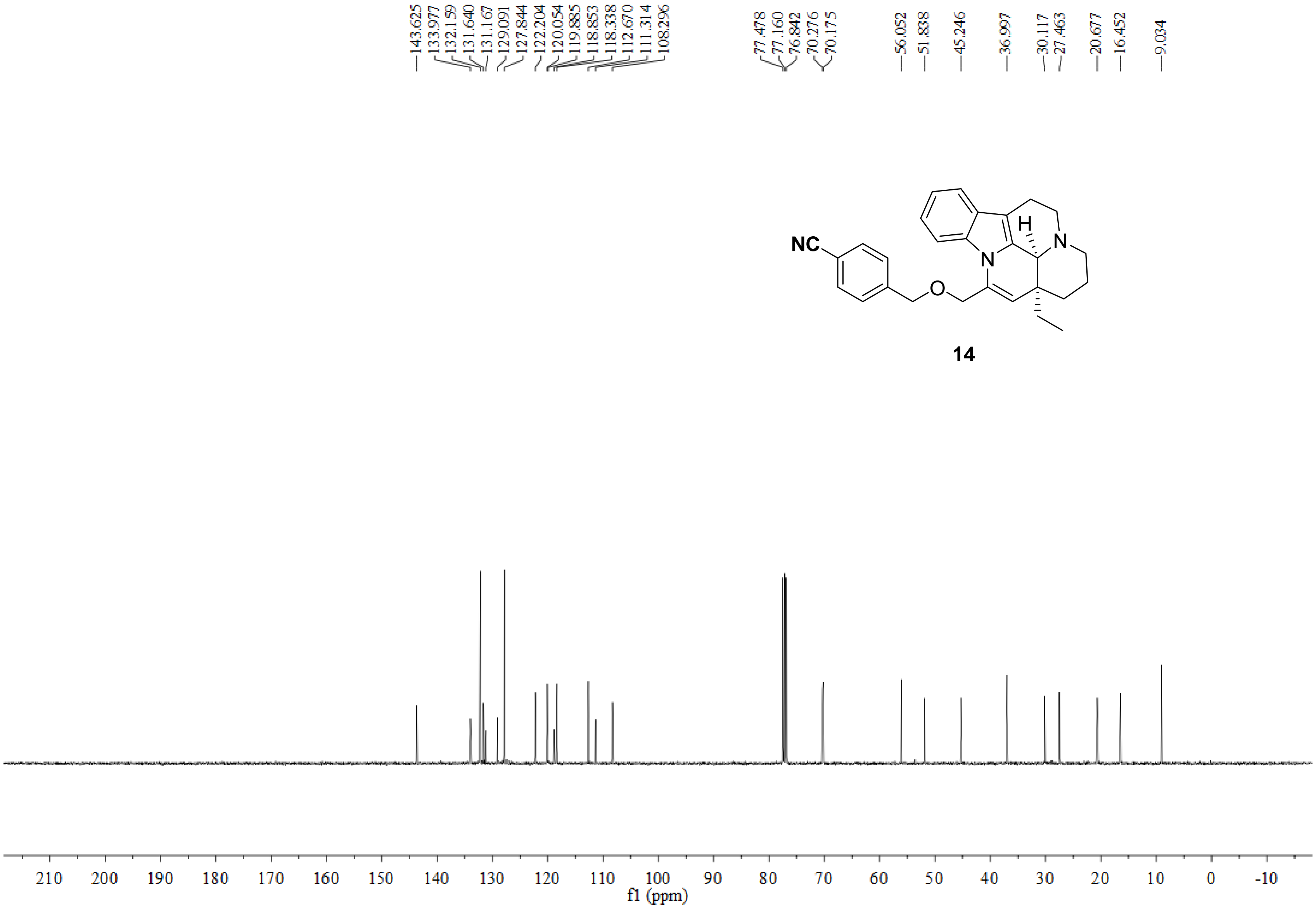

^{13}C NMR of Compound **14** (100 MHz, $CDCl_3$)

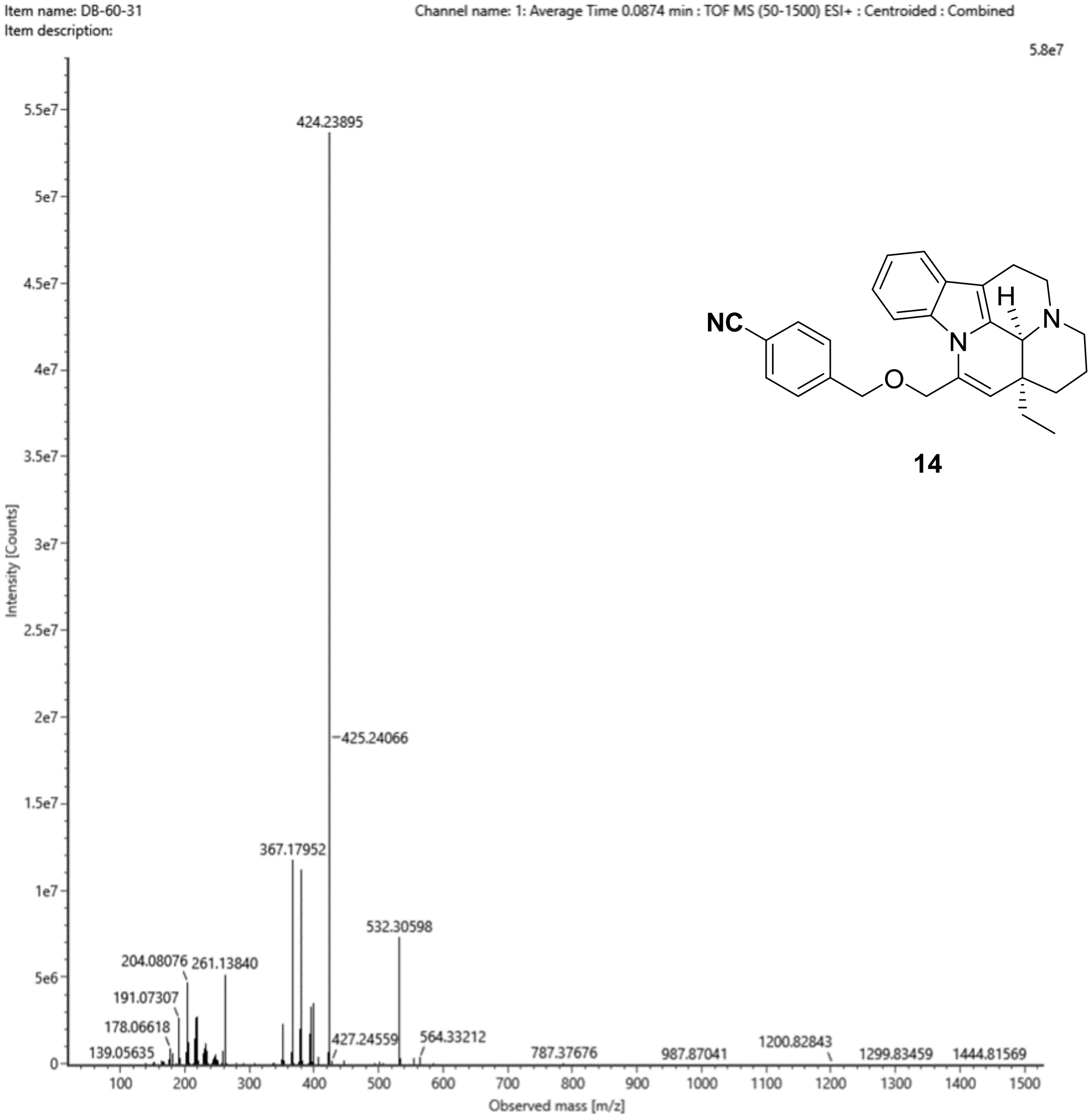

HRMS of Compound **14**

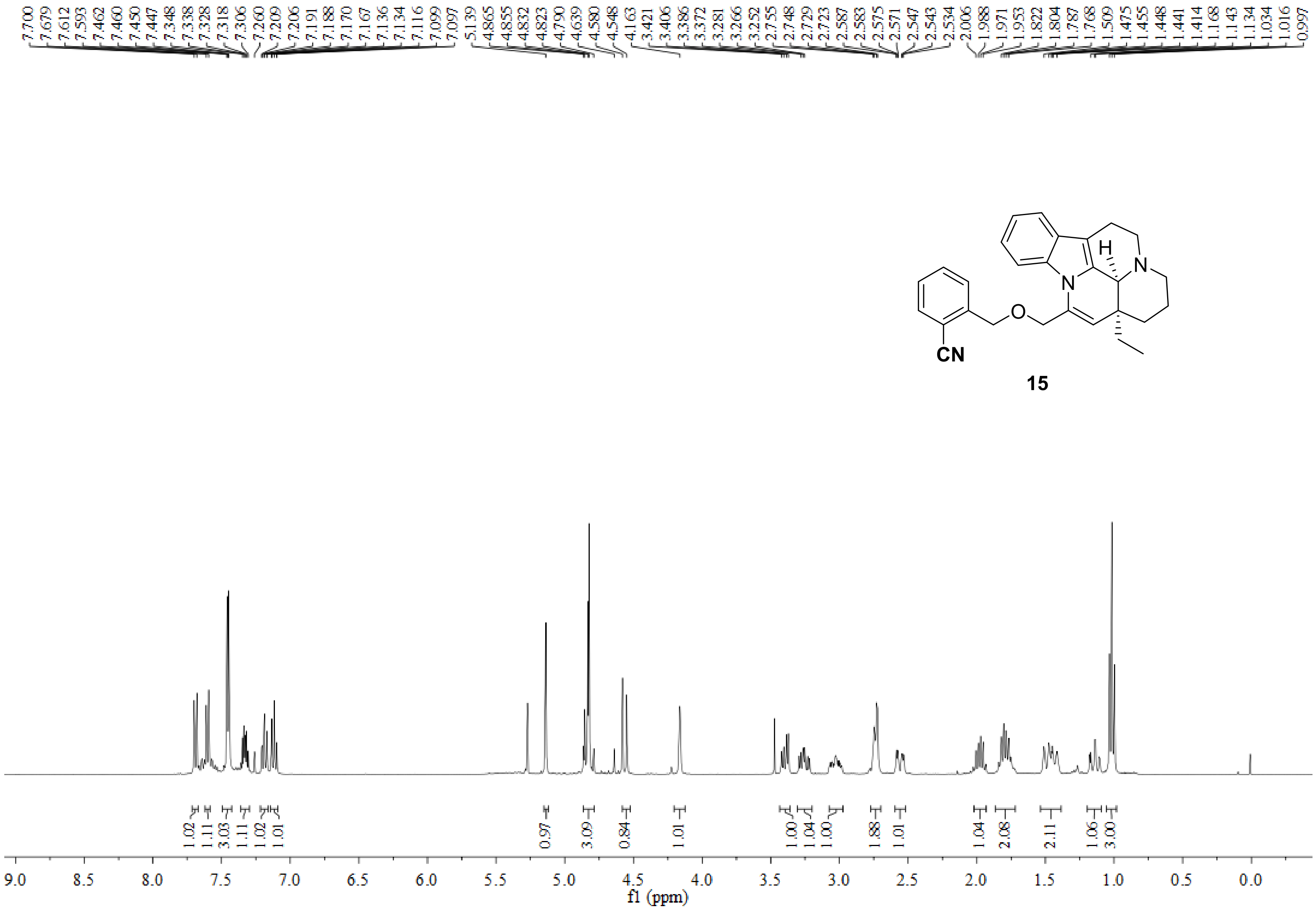

^{1}H NMR of Compound **15** (400 MHz, $CDCl_3$)

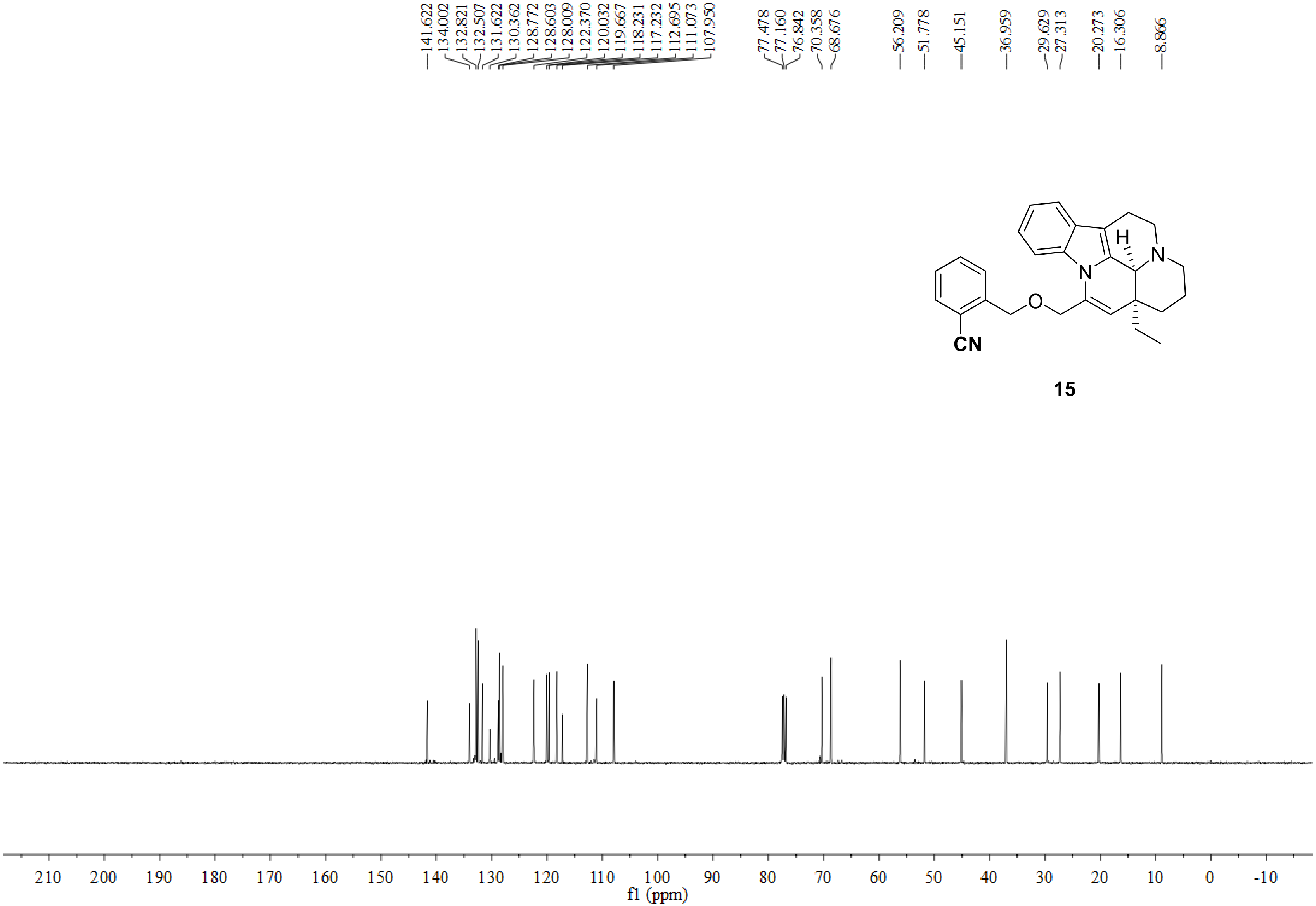

^{13}C NMR of Compound **15** (100 MHz, $CDCl_3$)

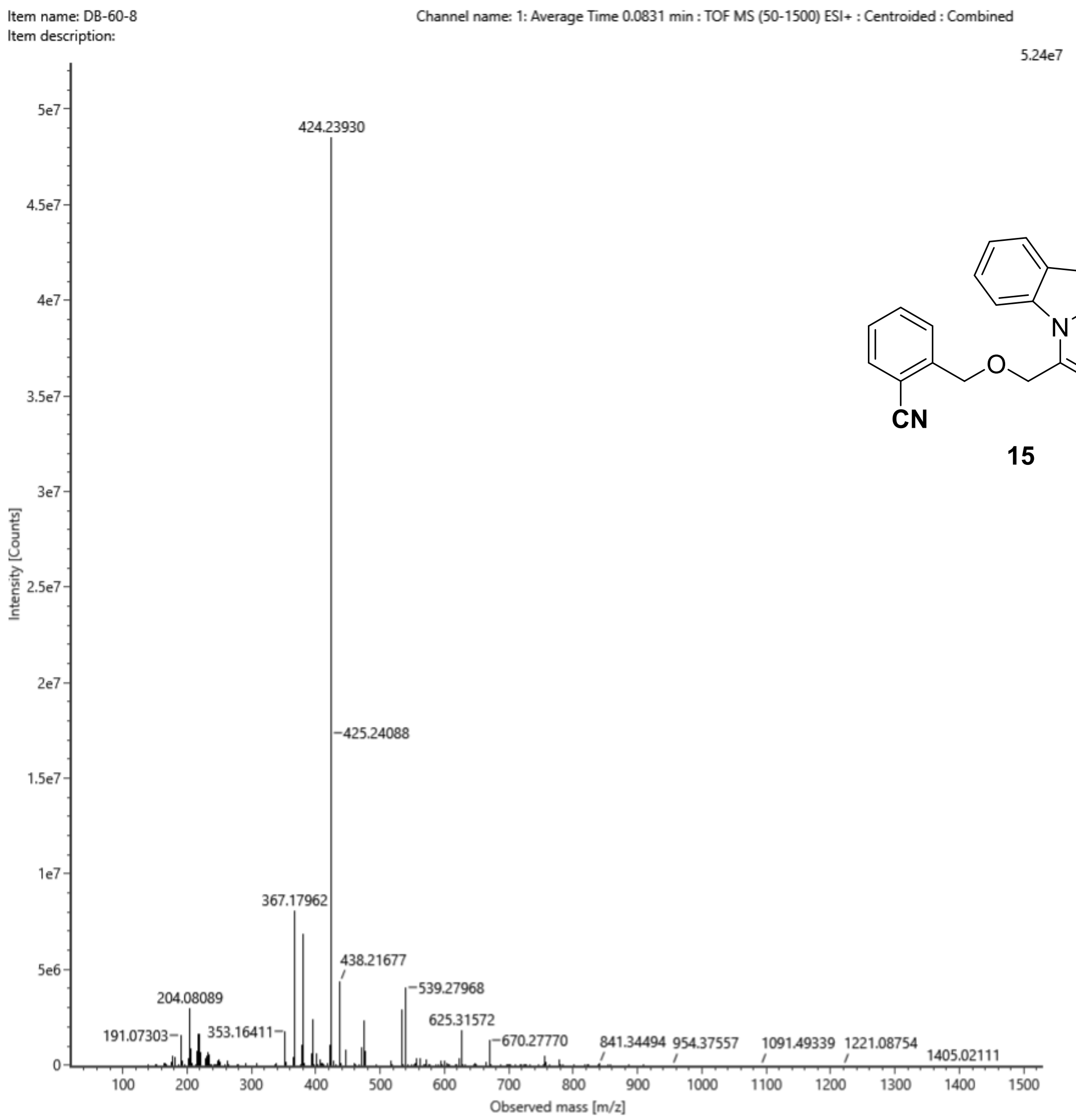

HRMS of Compound **15**

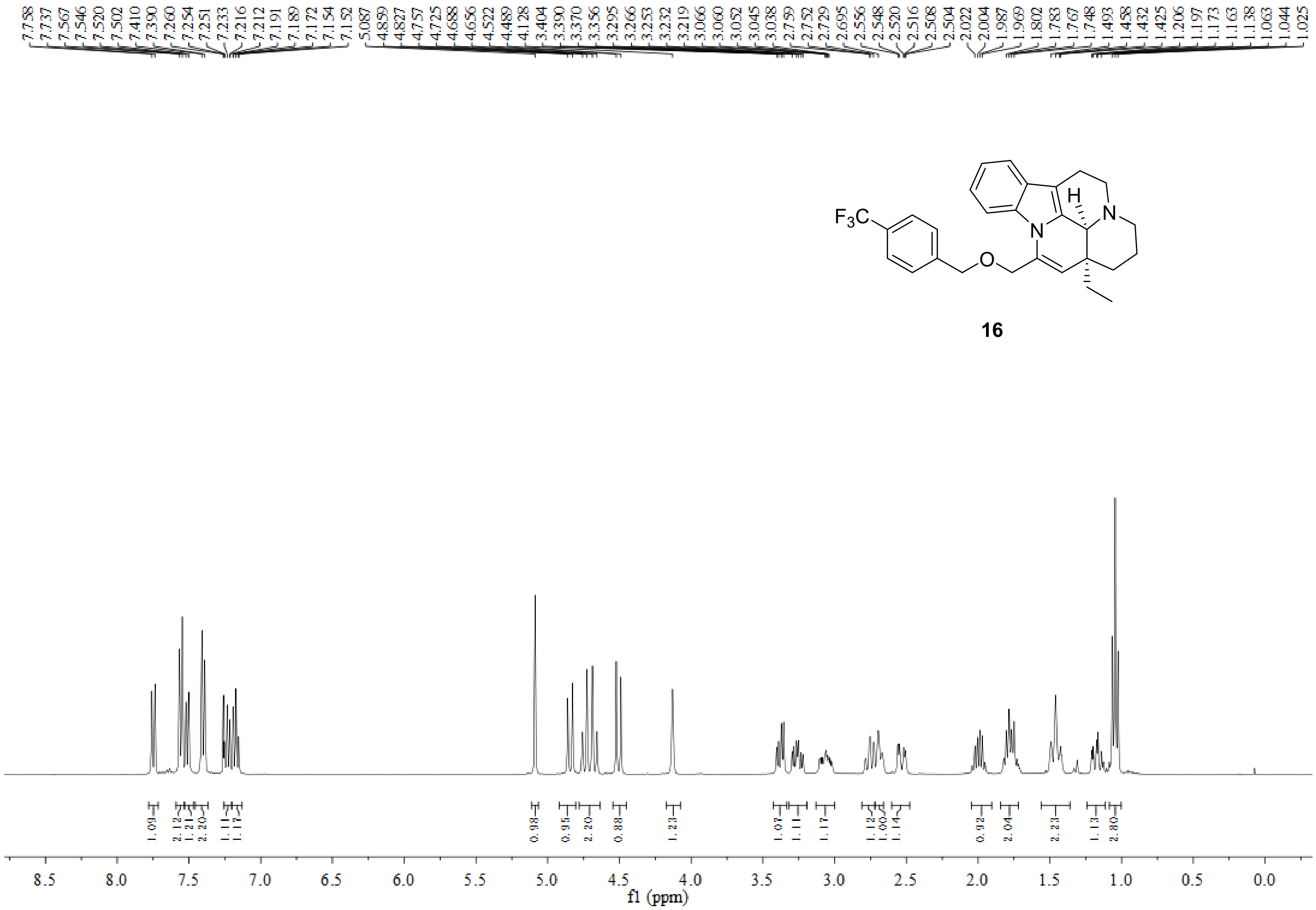

^{1}H NMR of Compound **16**(400 MHz, $CDCl_3$)

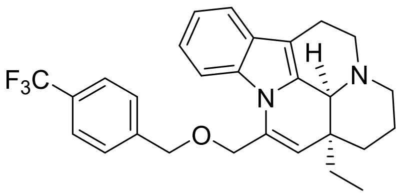

16

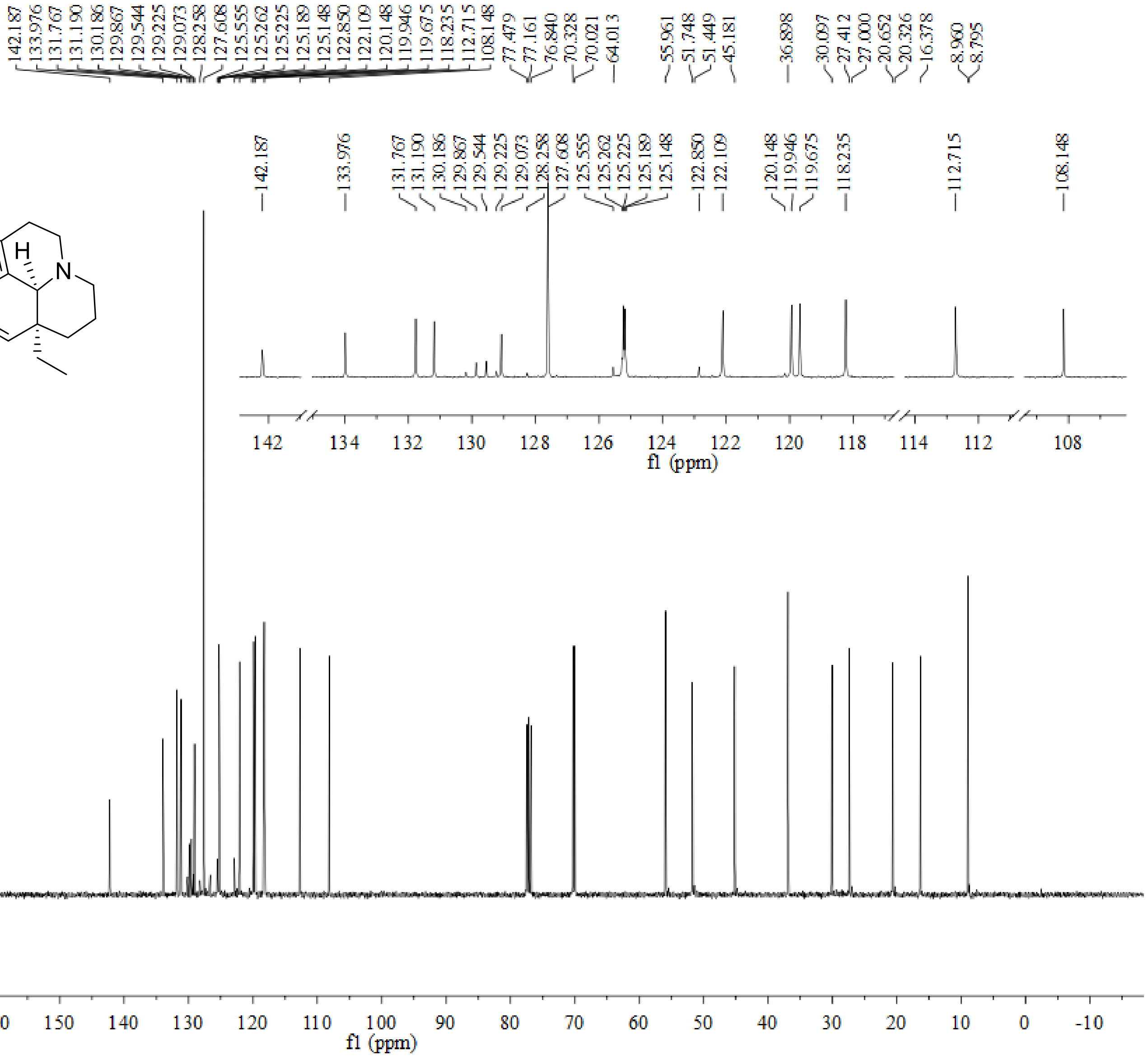

^{13}C NMR of Compound **16** (100 MHz, $CDCl_3$)

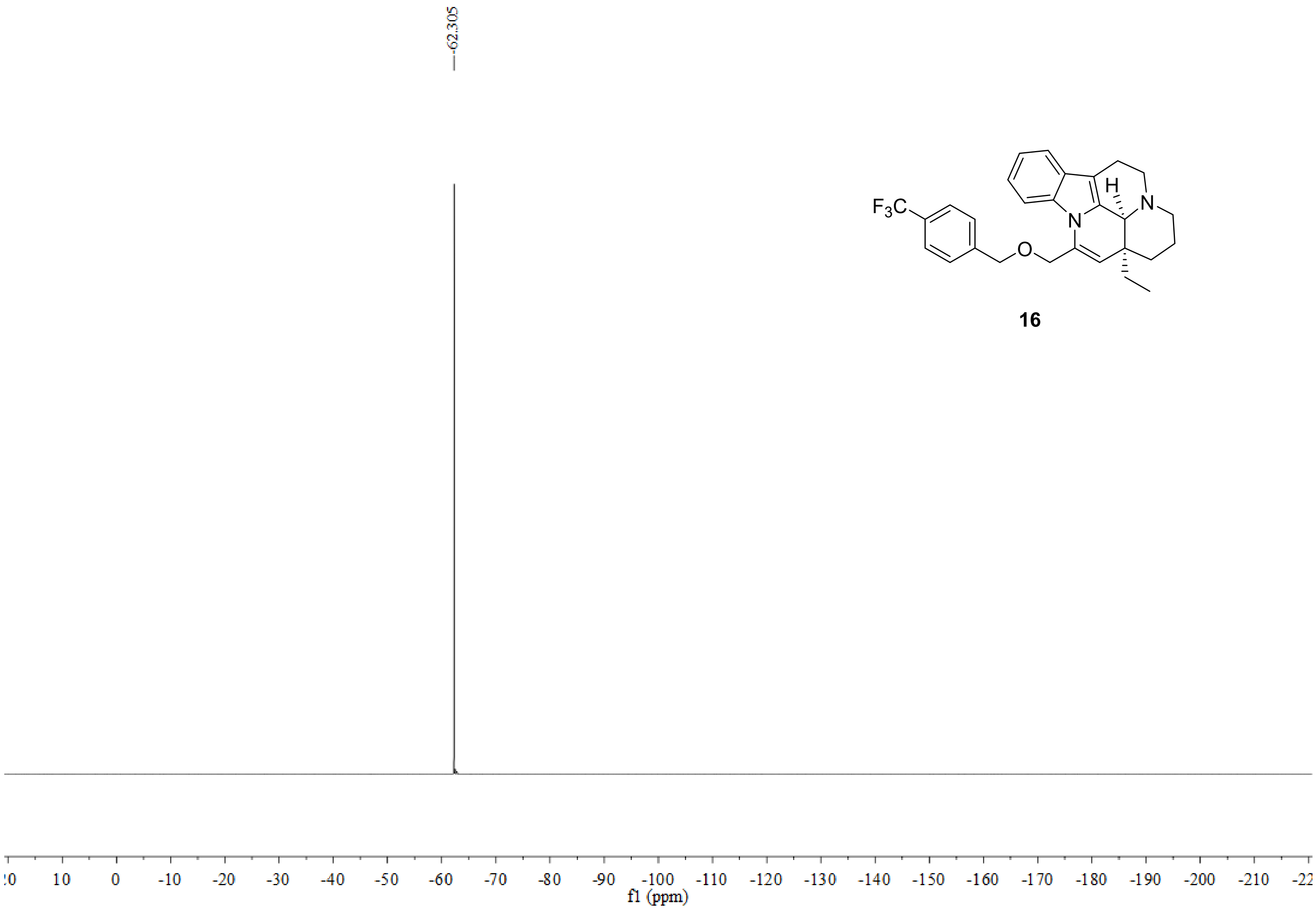

16

^{19}F NMR of Compound **16** (377 MHz, $CDCl_3$)

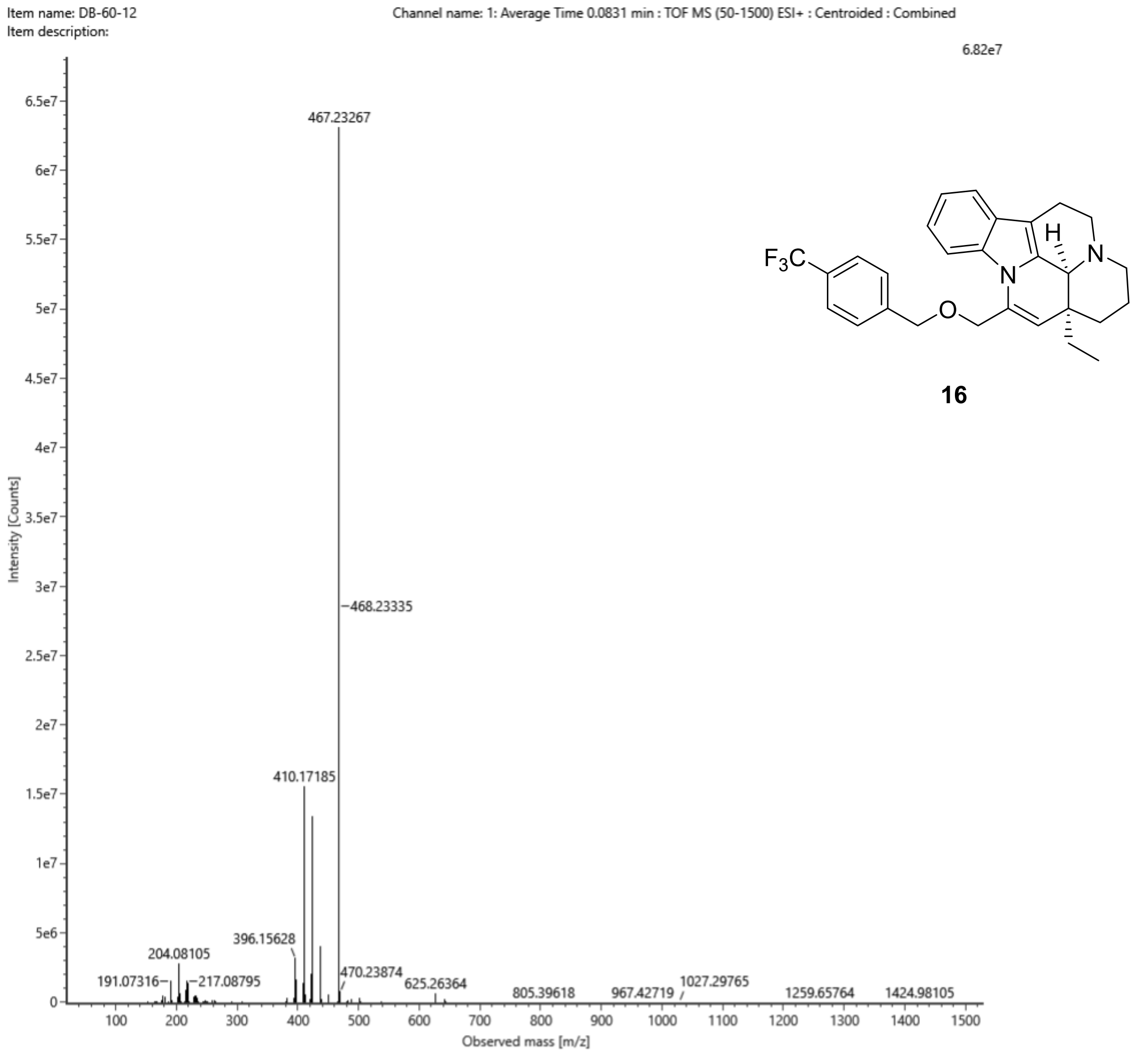

HRMS of Compound **16**

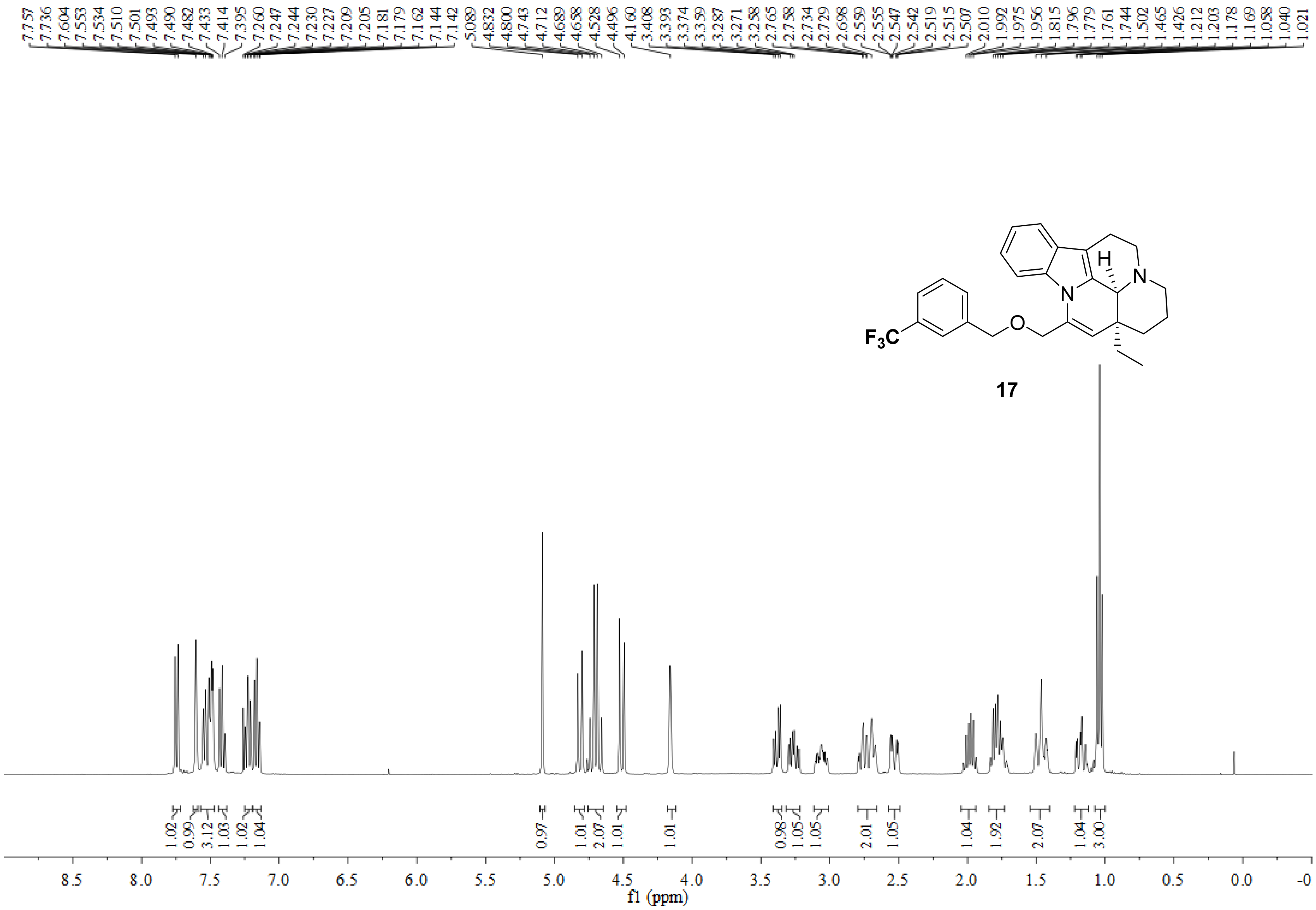

^{1}H NMR of Compound **17** (400 MHz, $CDCl_3$)

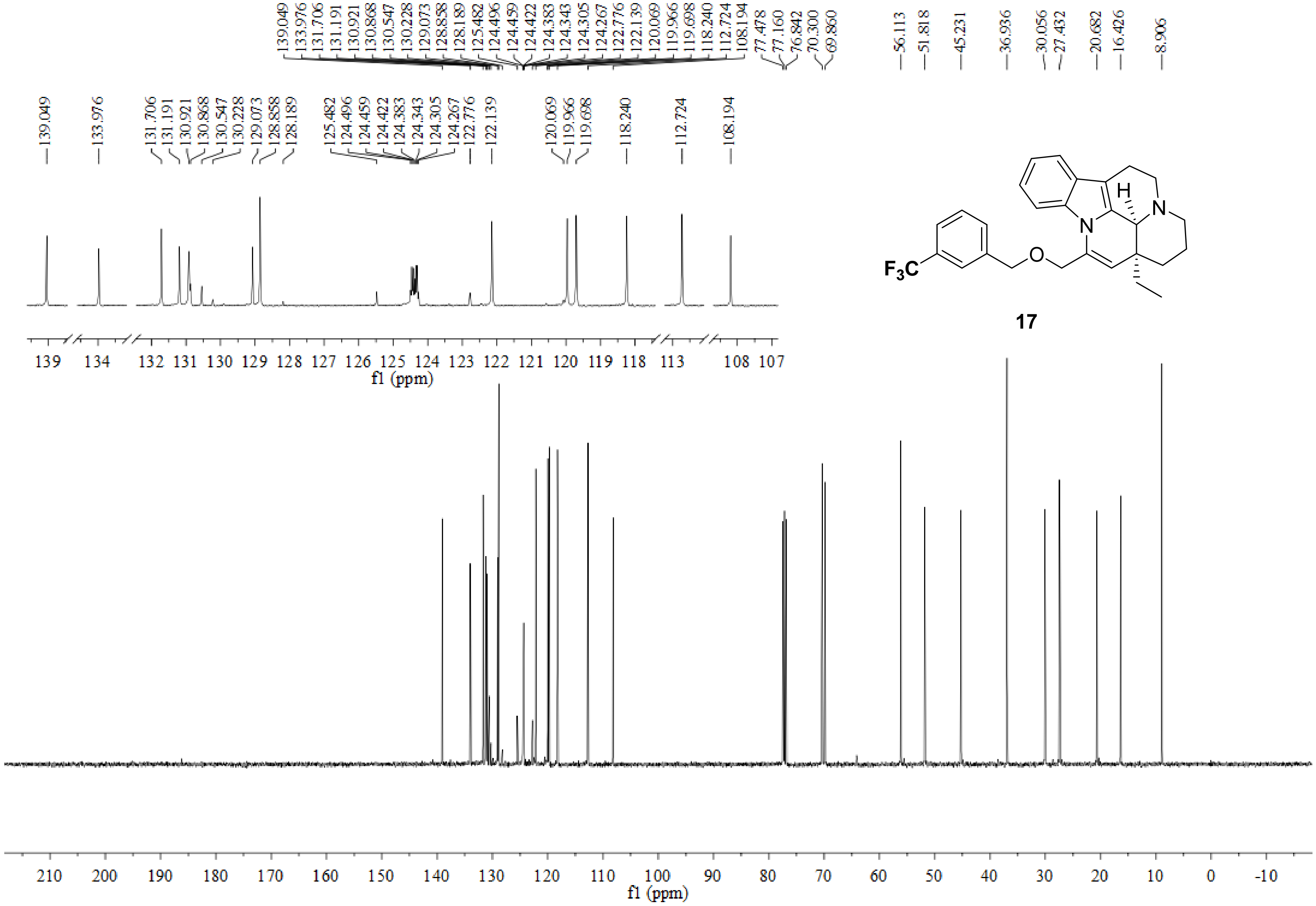

^{13}C NMR of Compound **17** (100 MHz, $CDCl_3$)

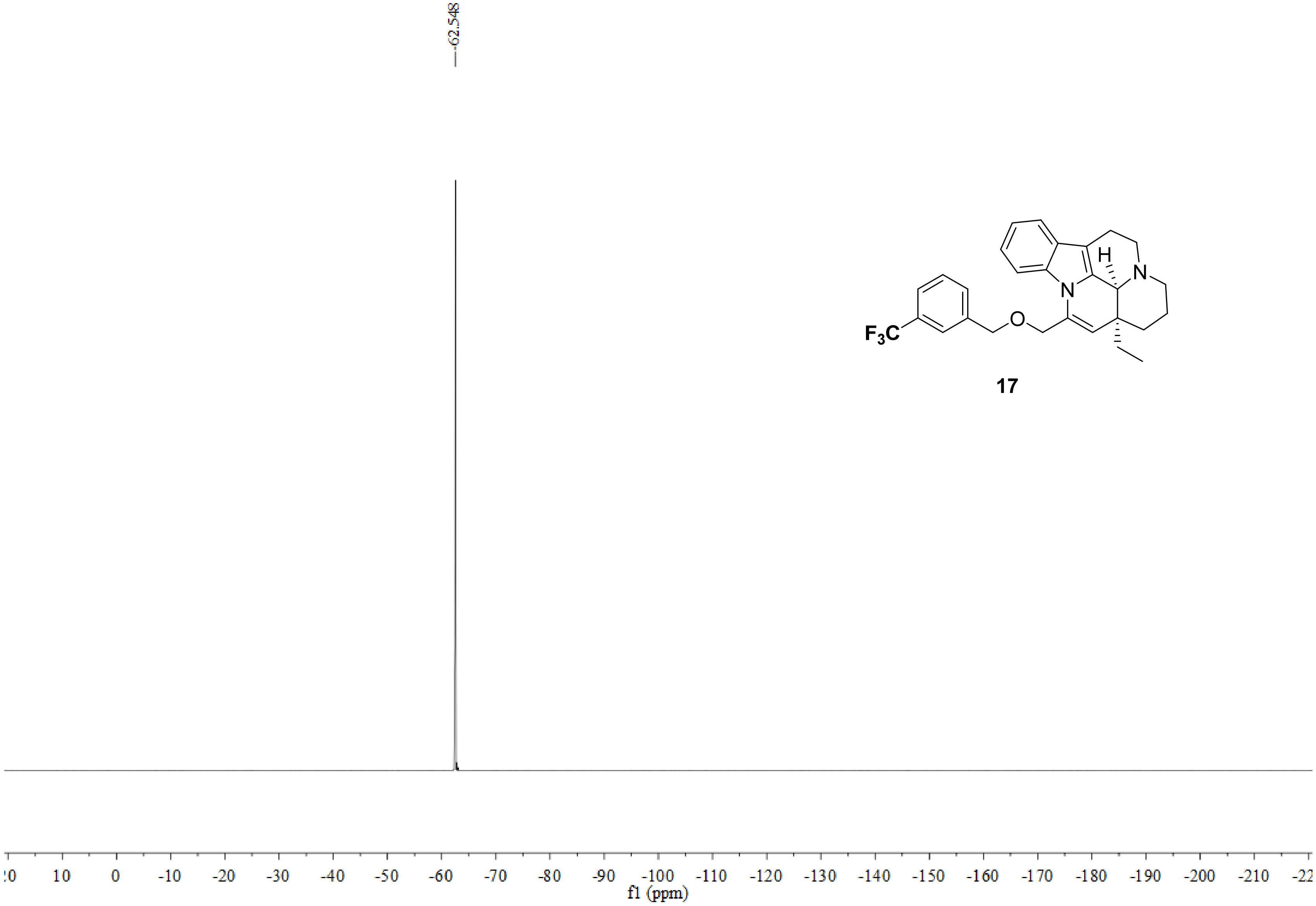

17

^{19}F NMR of Compound **17** (377 MHz, $CDCl_3$)

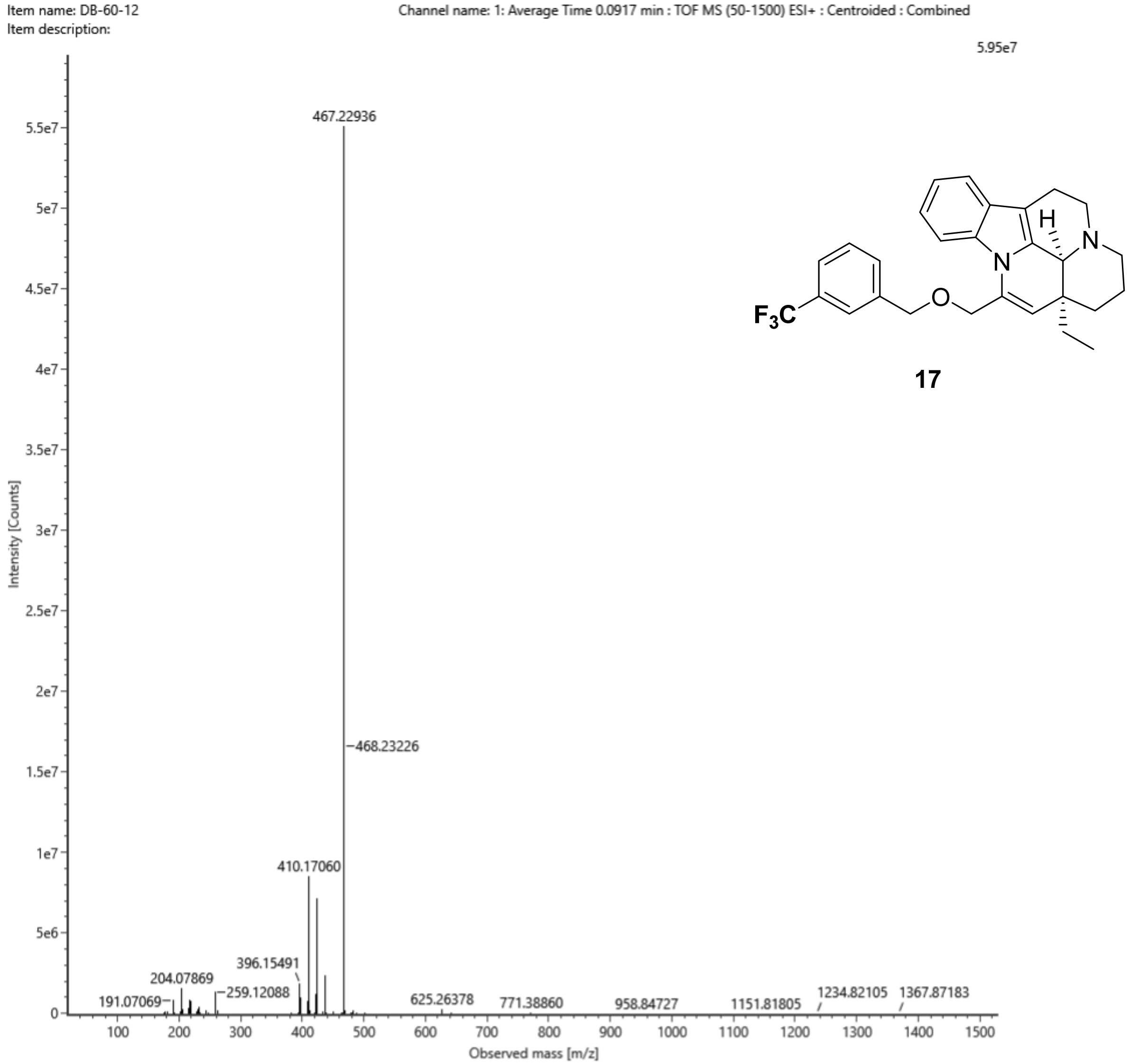

17

HRMS of Compound **17**

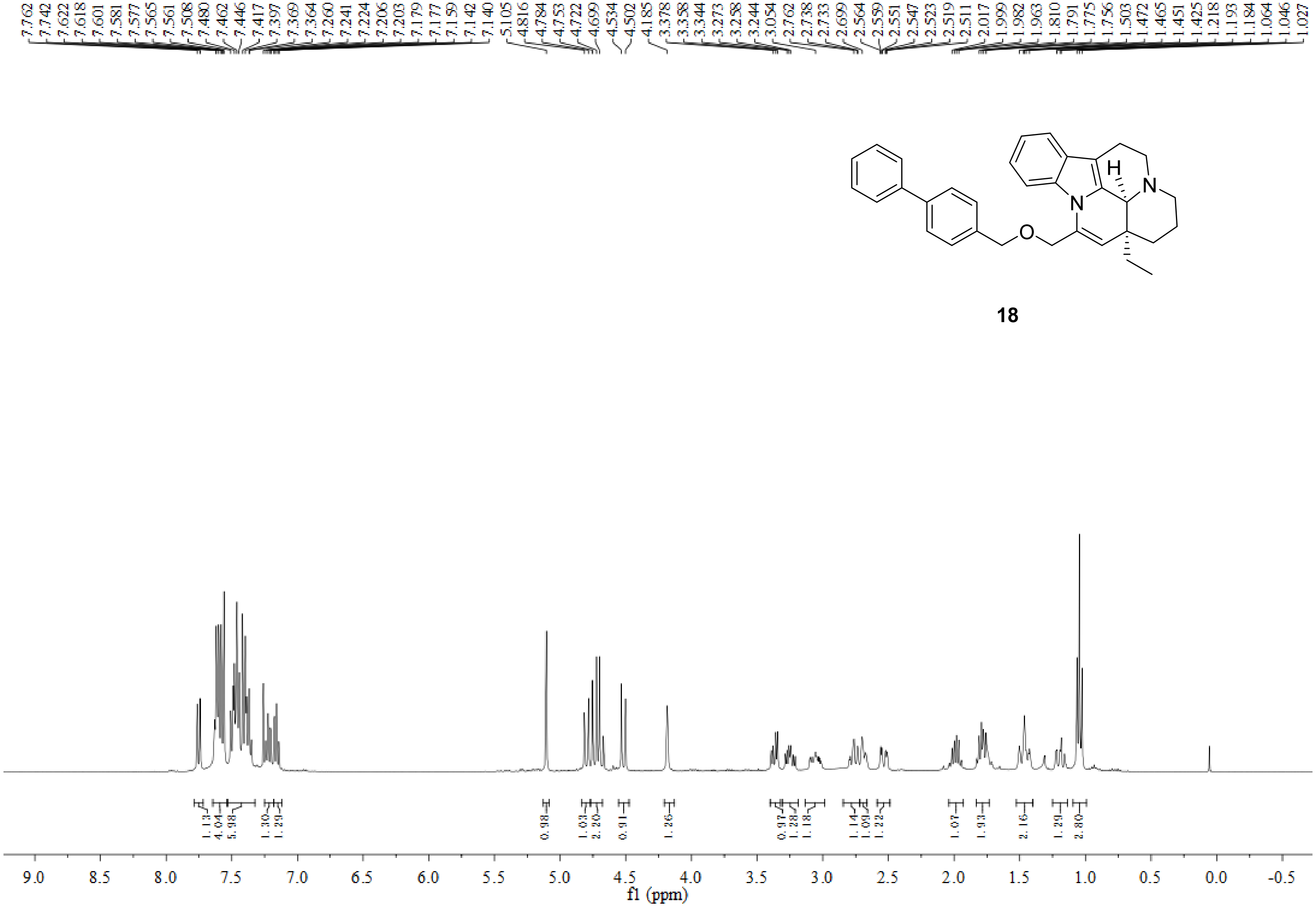

18

^{1}H NMR of Compound 18 (400 MHz, $CDCl_3$)

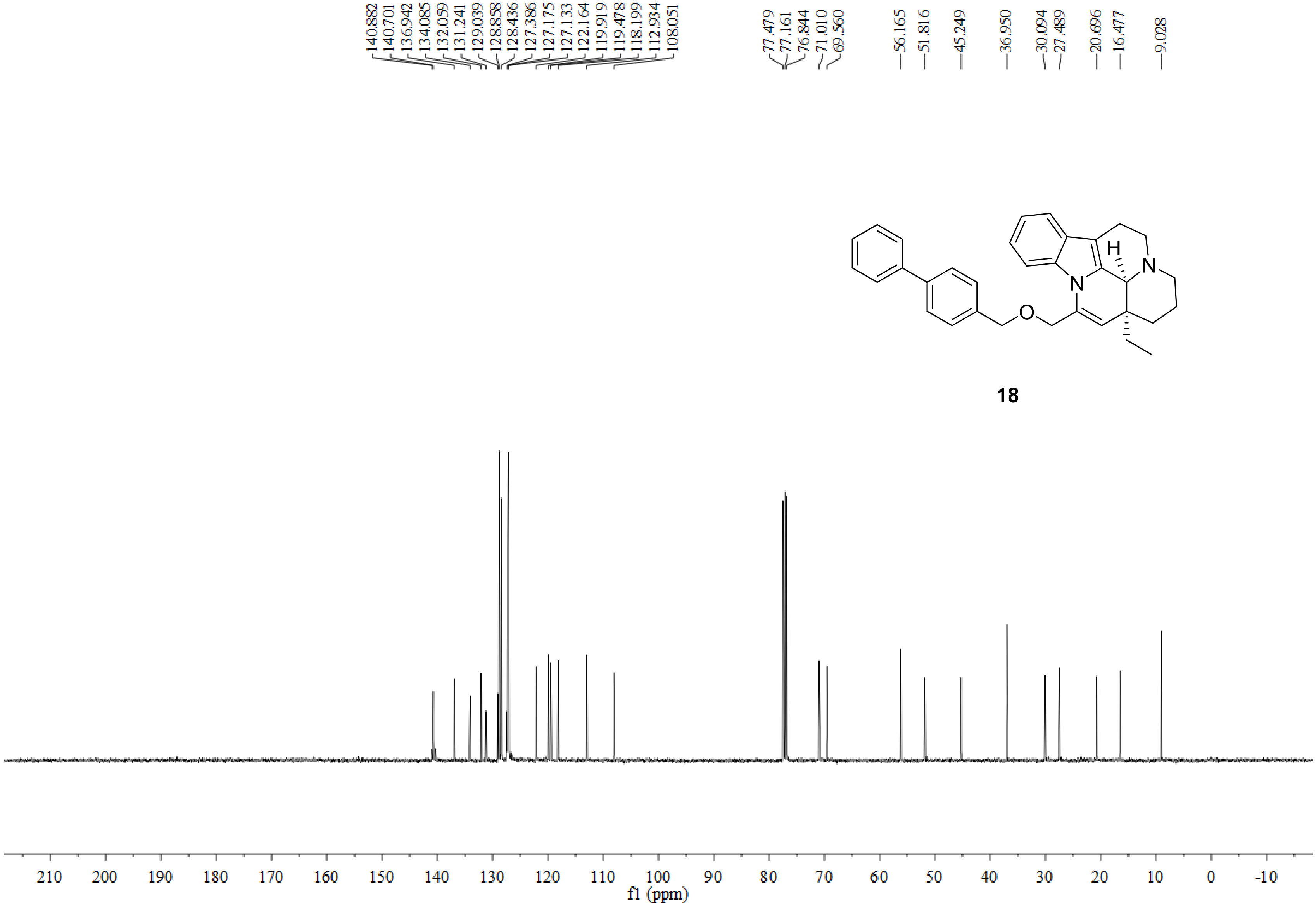

18

^{13}C NMR of Compound**18** (100 MHz, $CDCl_3$)

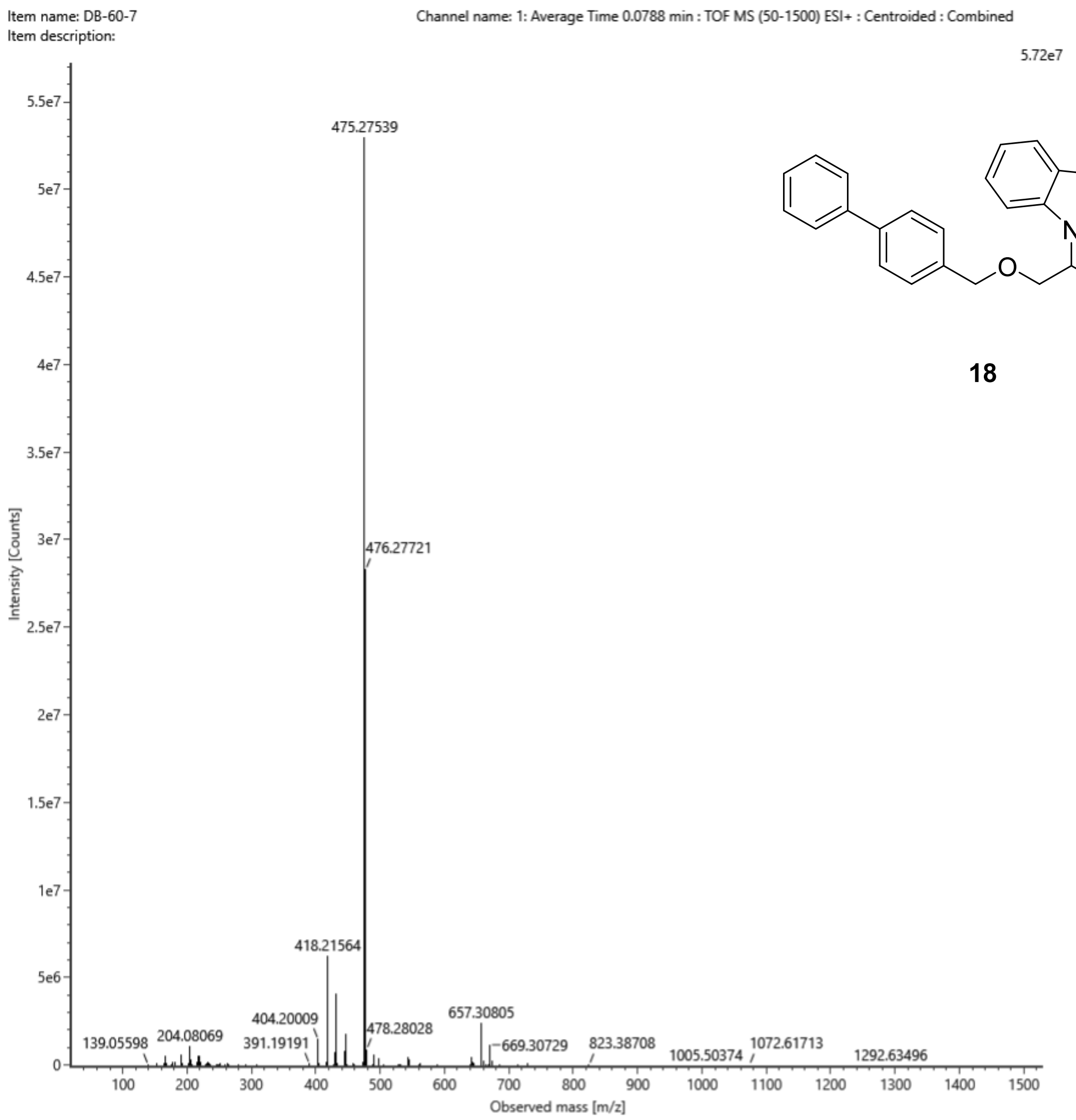

HRMS of Compound **18**

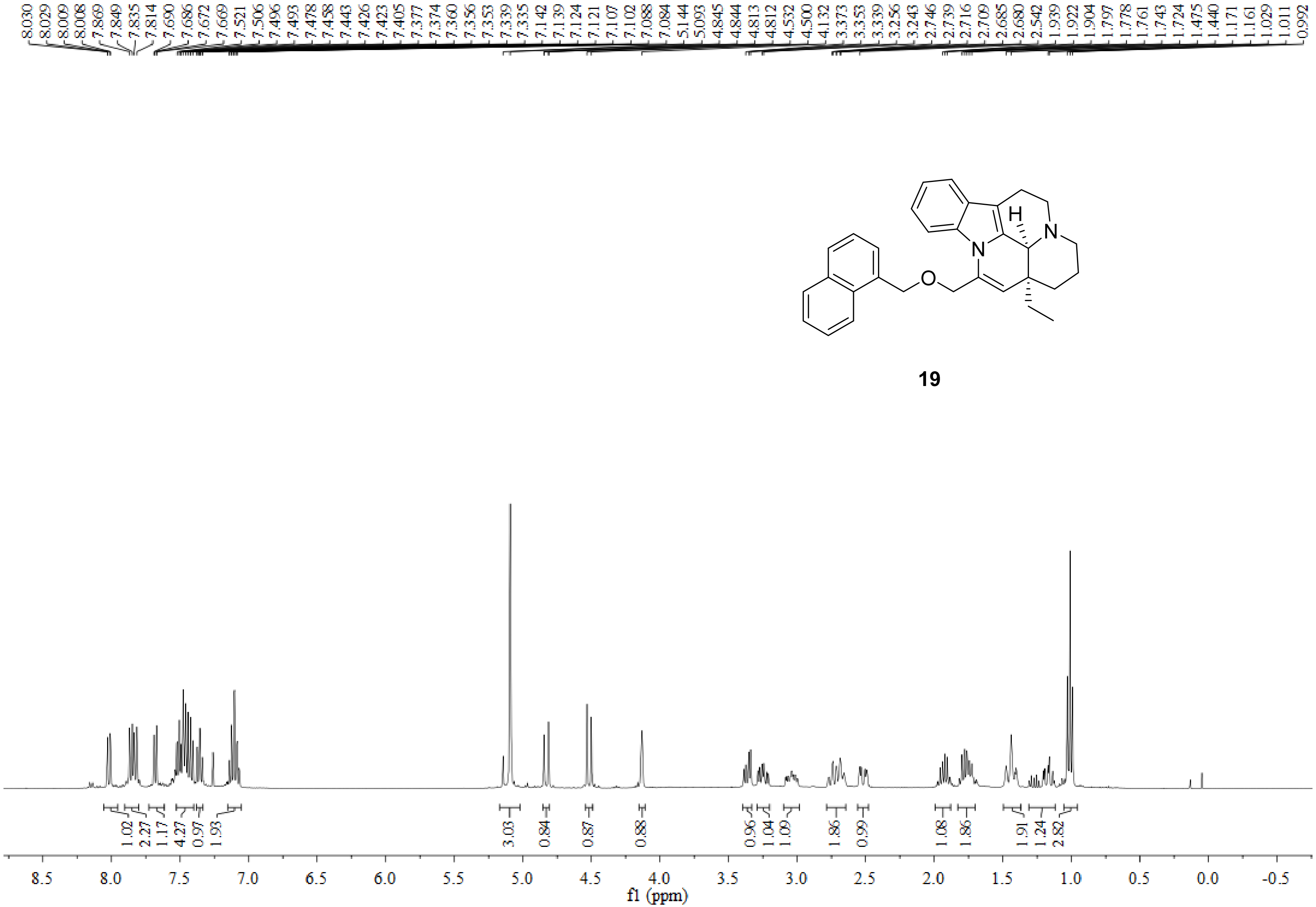

19

^{1}H NMR of Compound **19** (400 MHz, $CDCl_3$)

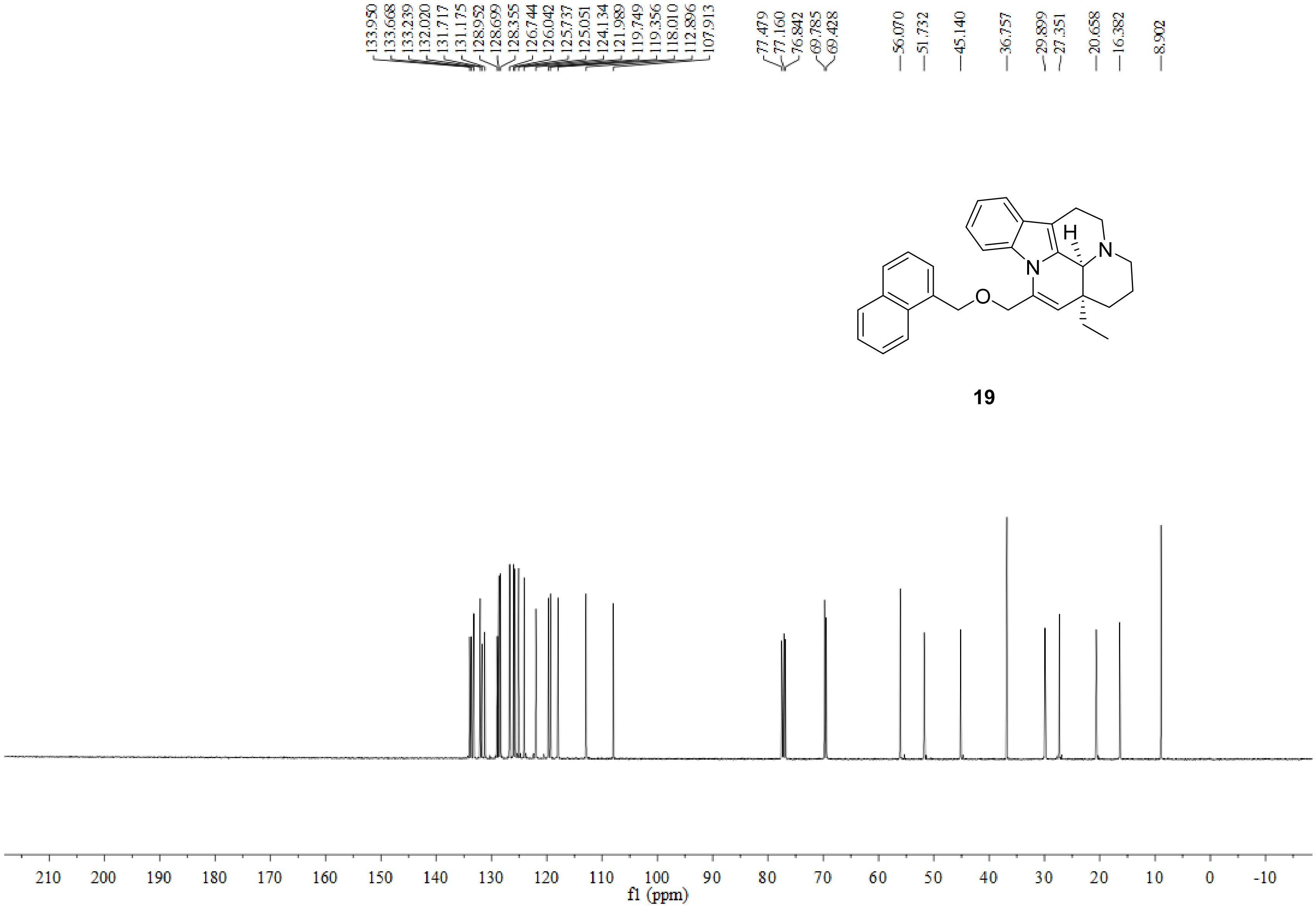

19

^{13}C NMR of Compound **19** (100 MHz, $CDCl_3$)

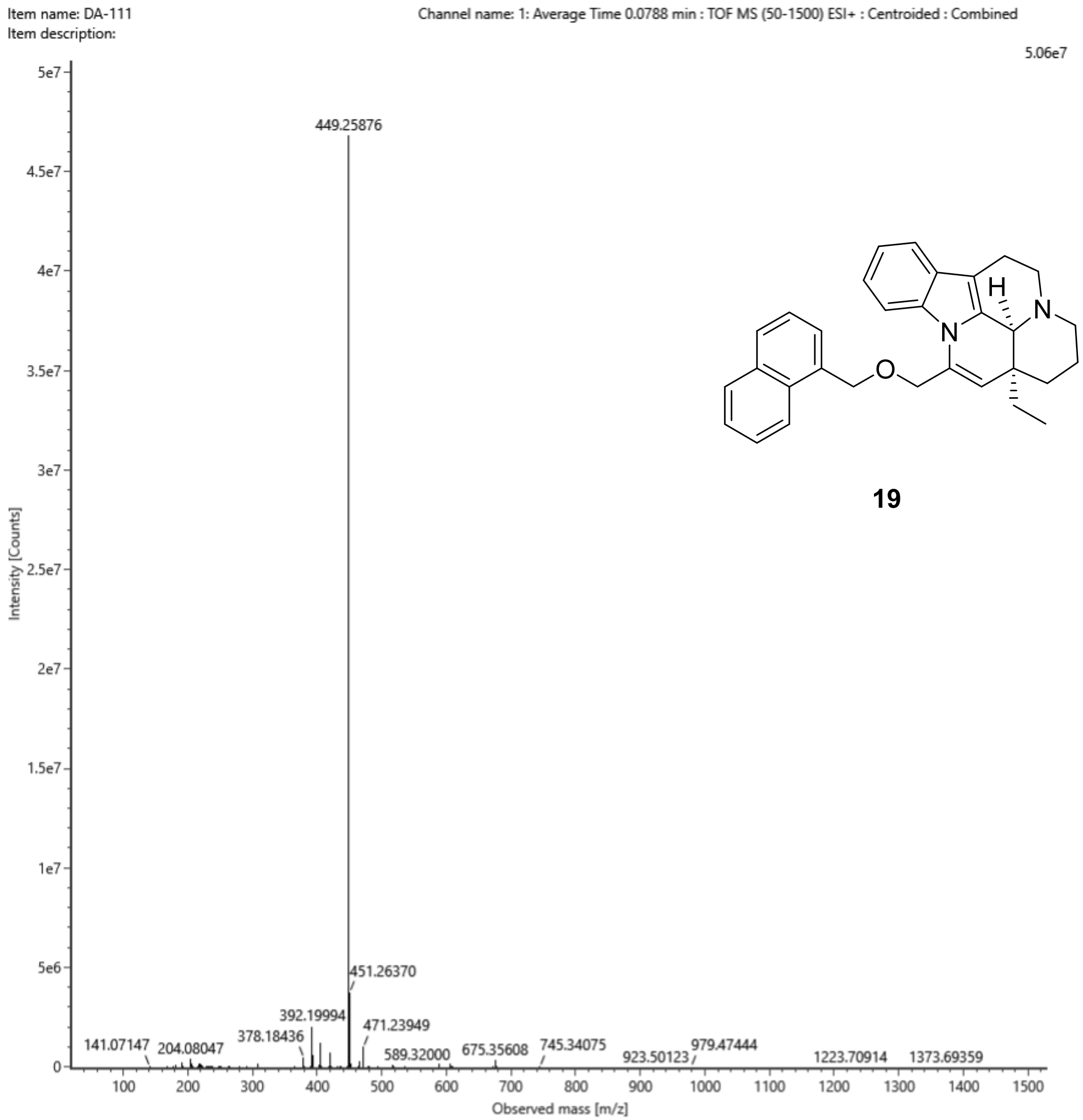

HRMS of Compound **19**

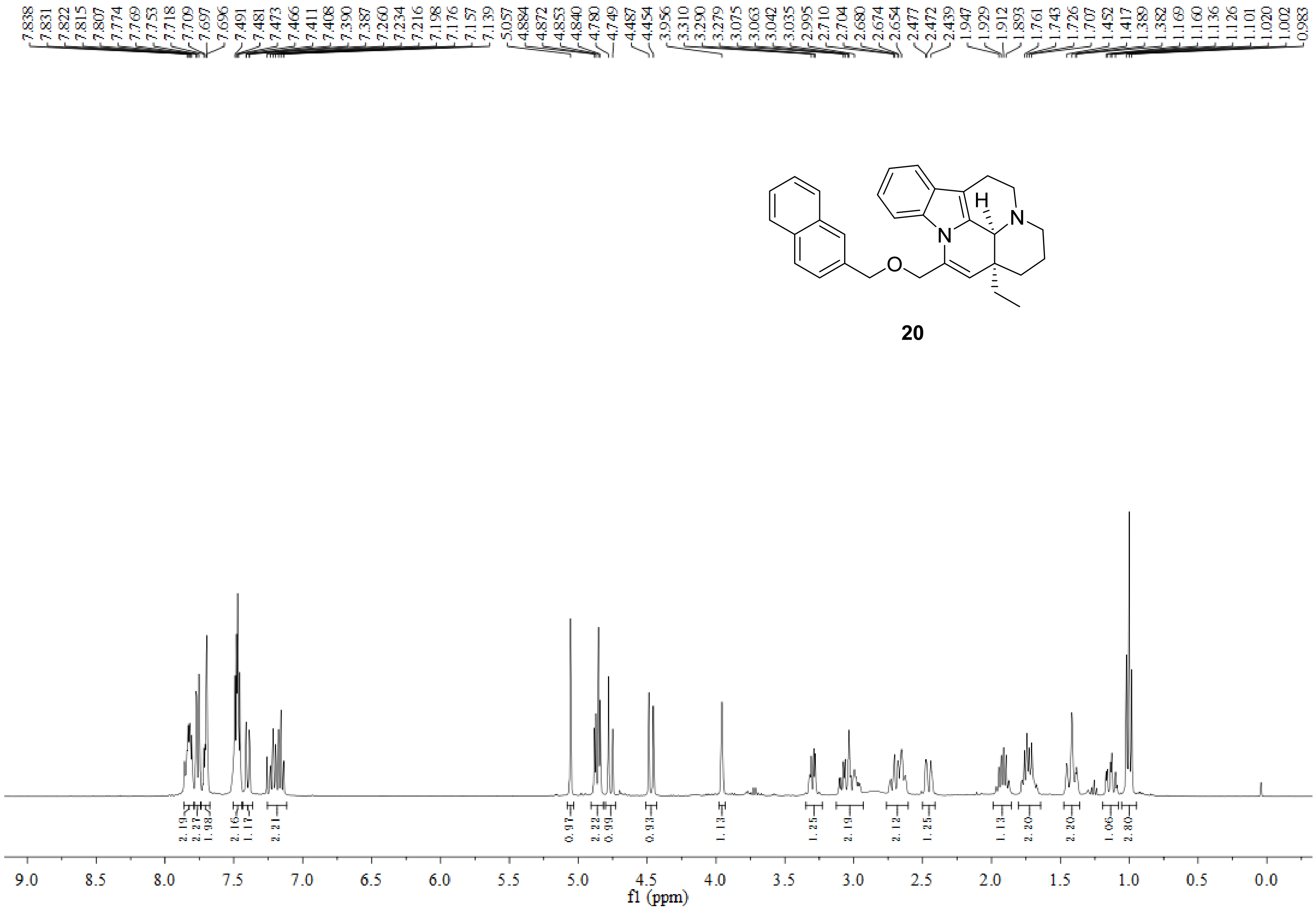

^{1}H NMR of Compound **20** (400 MHz, $CDCl_3$)

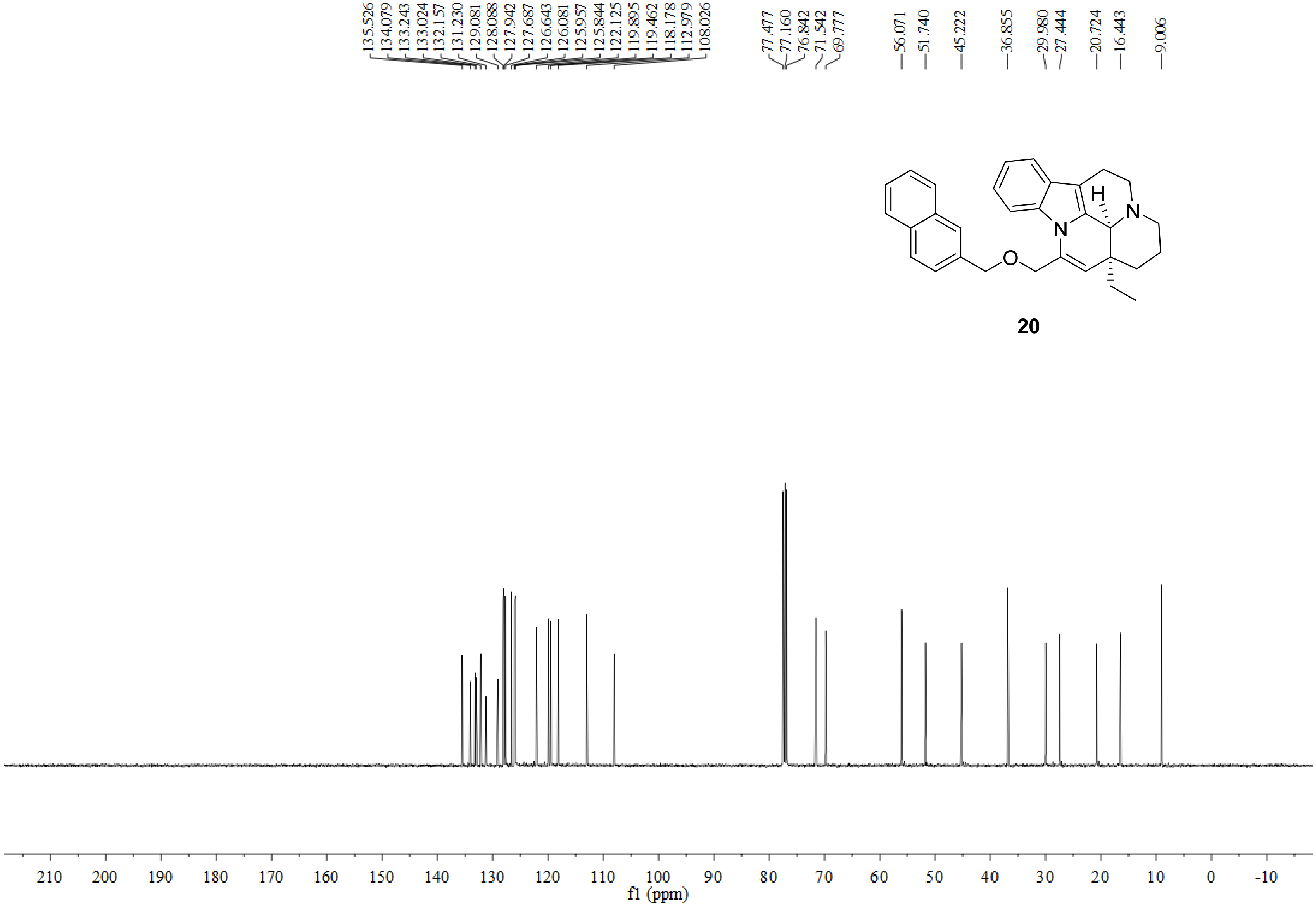

20

^{13}C NMR of Compound **20** (100 MHz, $CDCl_3$)

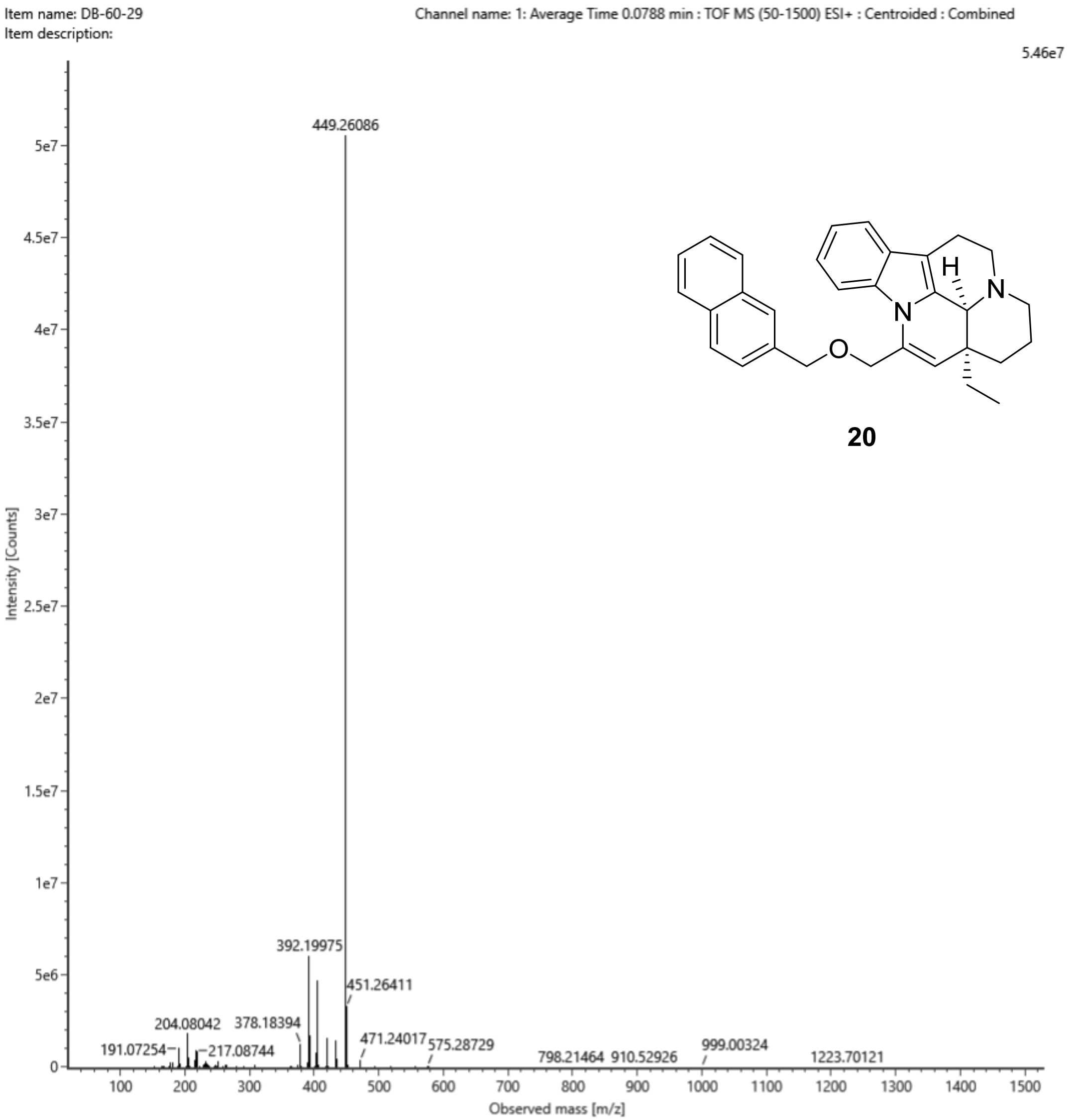

HRMS of Compound **20**

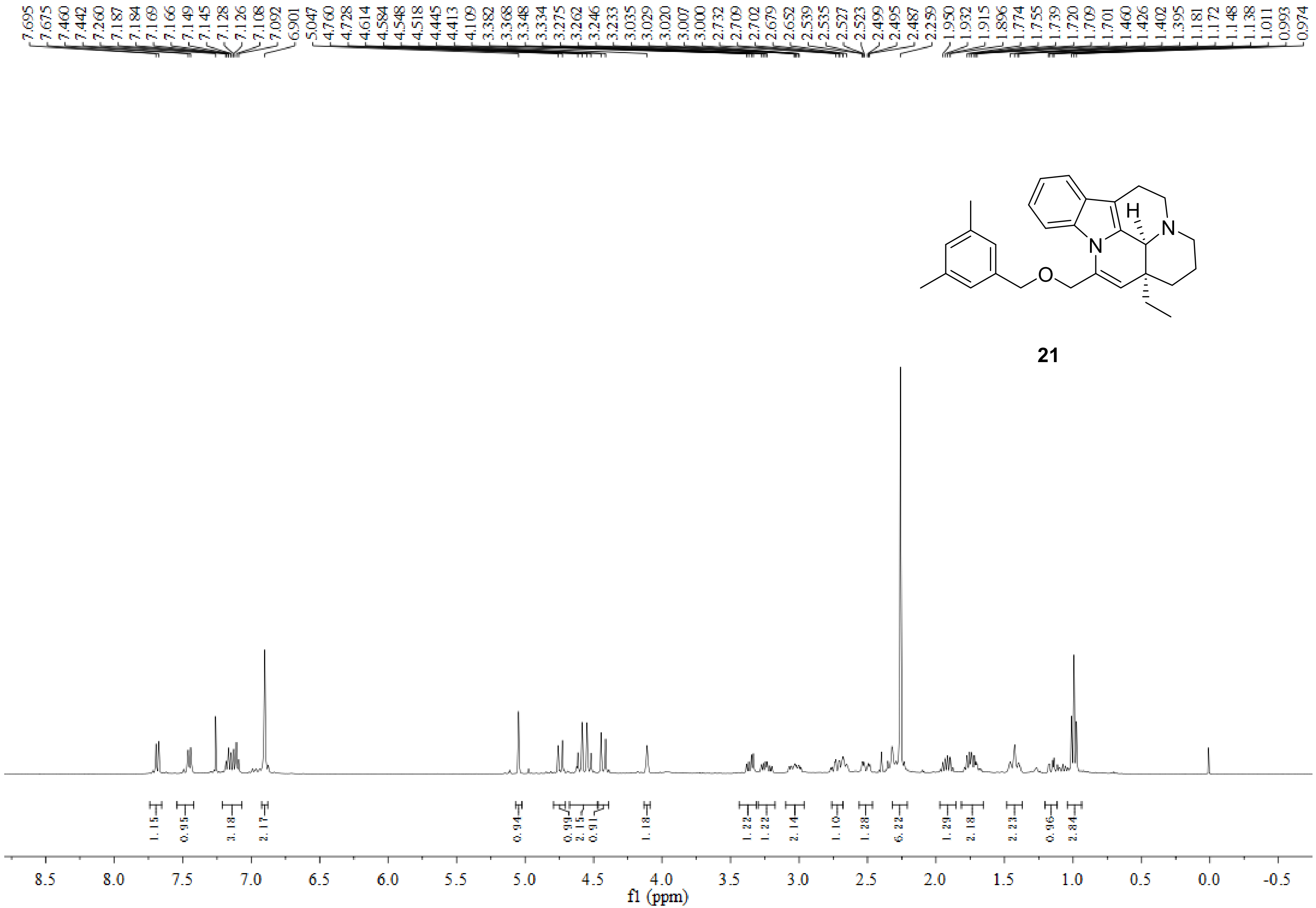

^{1}H NMR of Compound **21** (400 MHz, $CDCl_3$)

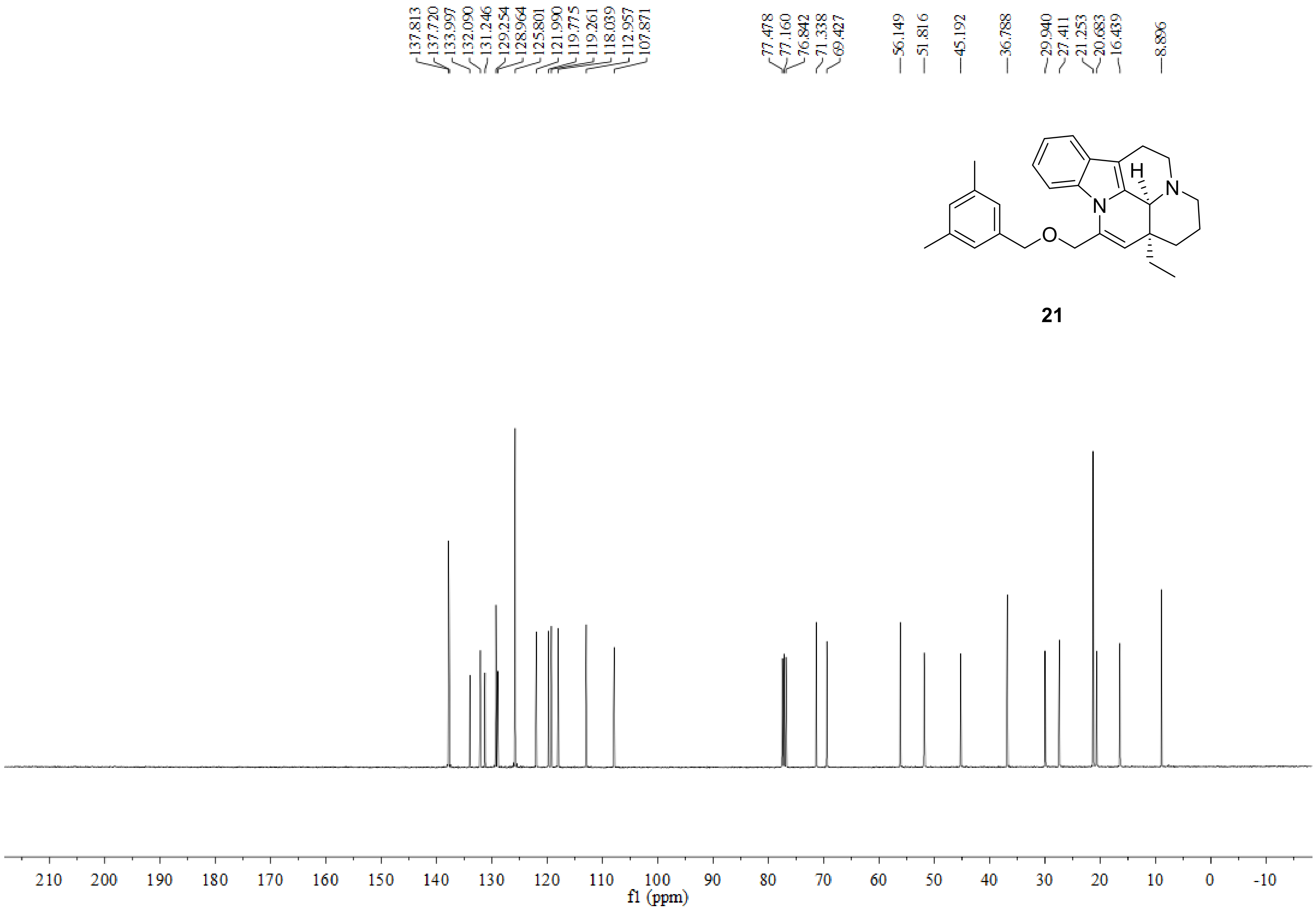

^{13}C NMR of Compound **21** (100 MHz, $CDCl_3$)

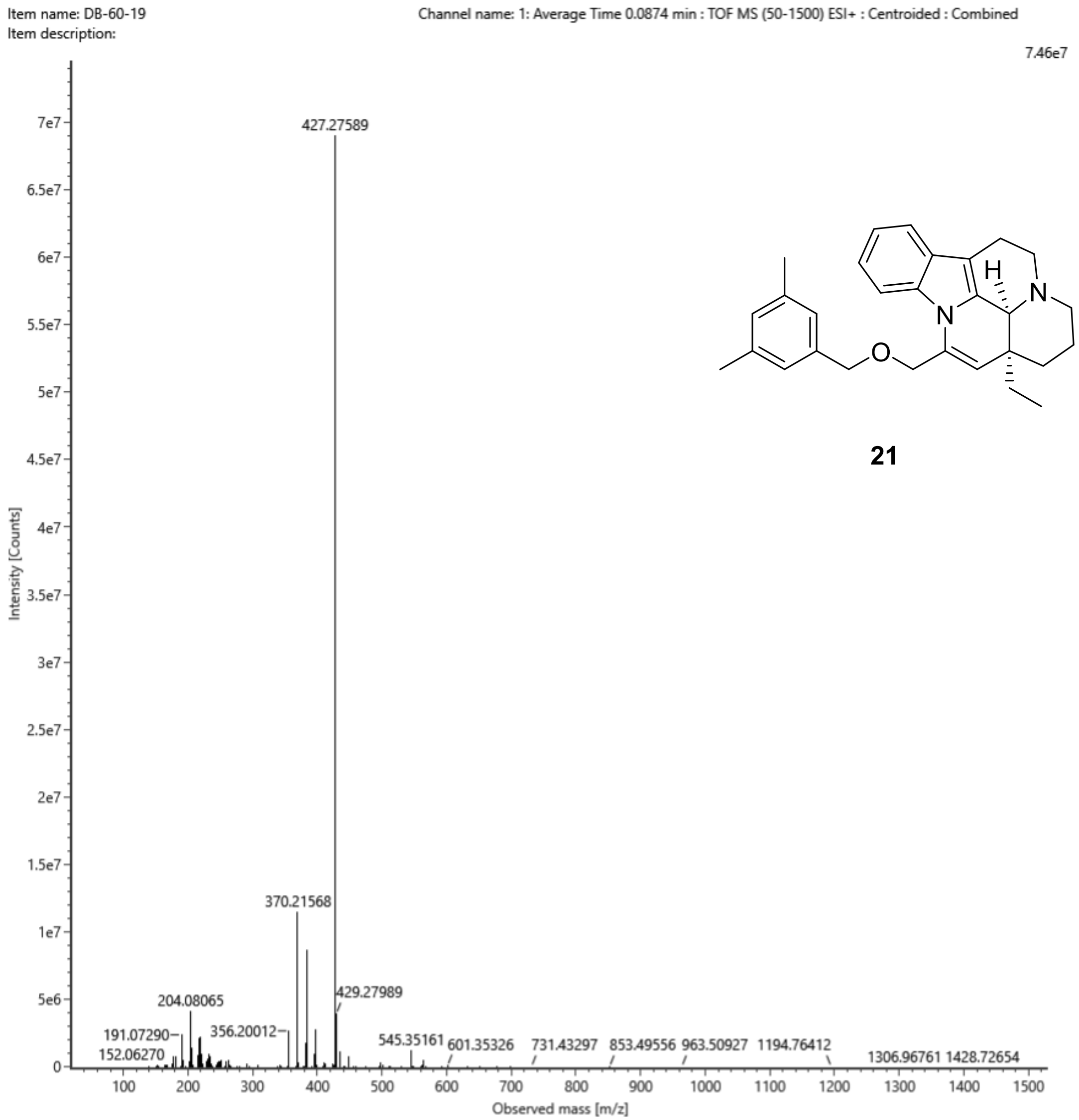

HRMS of Compound **21**

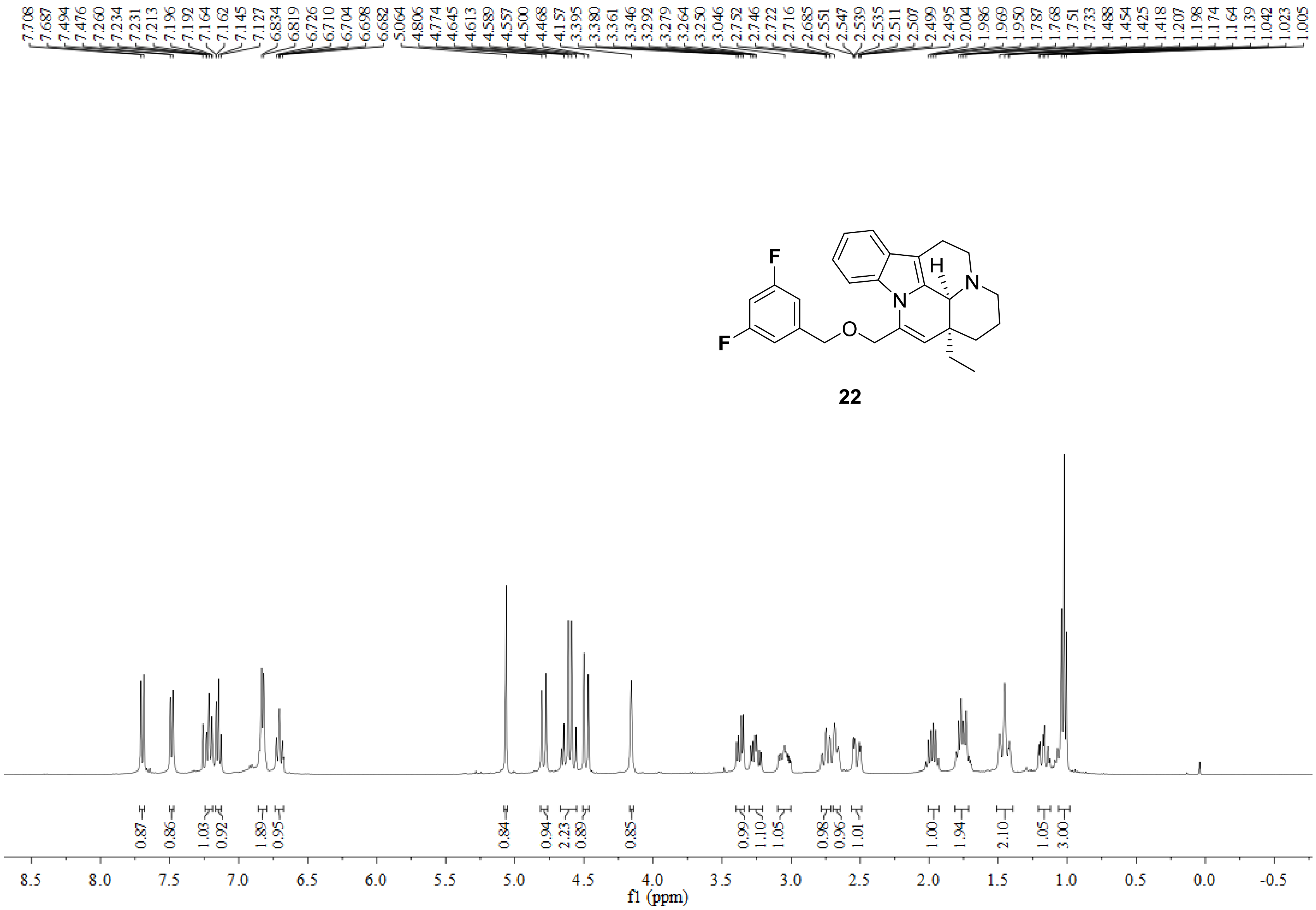

^{1}H NMR of Compound **22** (400 MHz, $CDCl_3$)

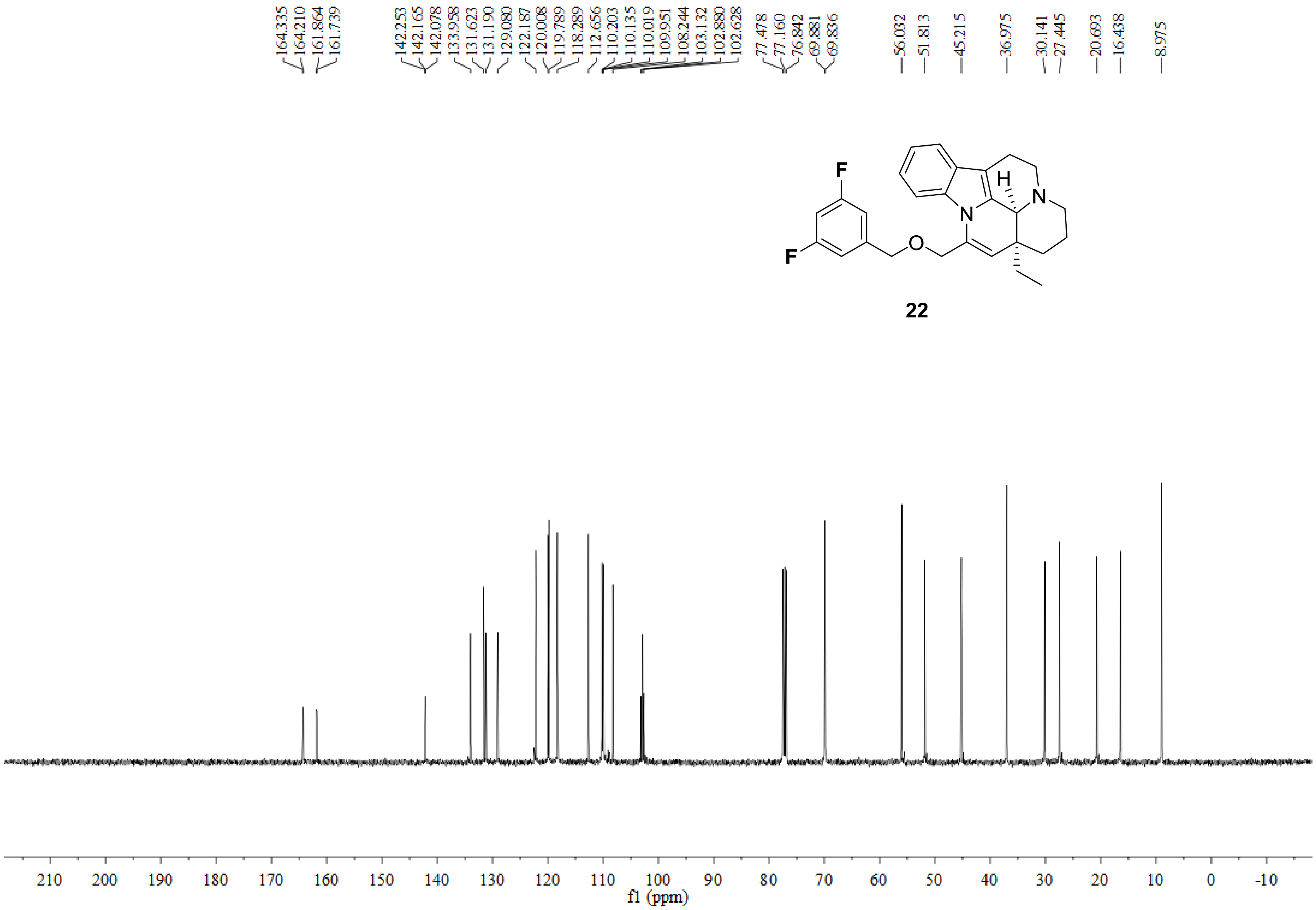

^{13}C NMR of Compound **22** (100 MHz, $CDCl_3$)

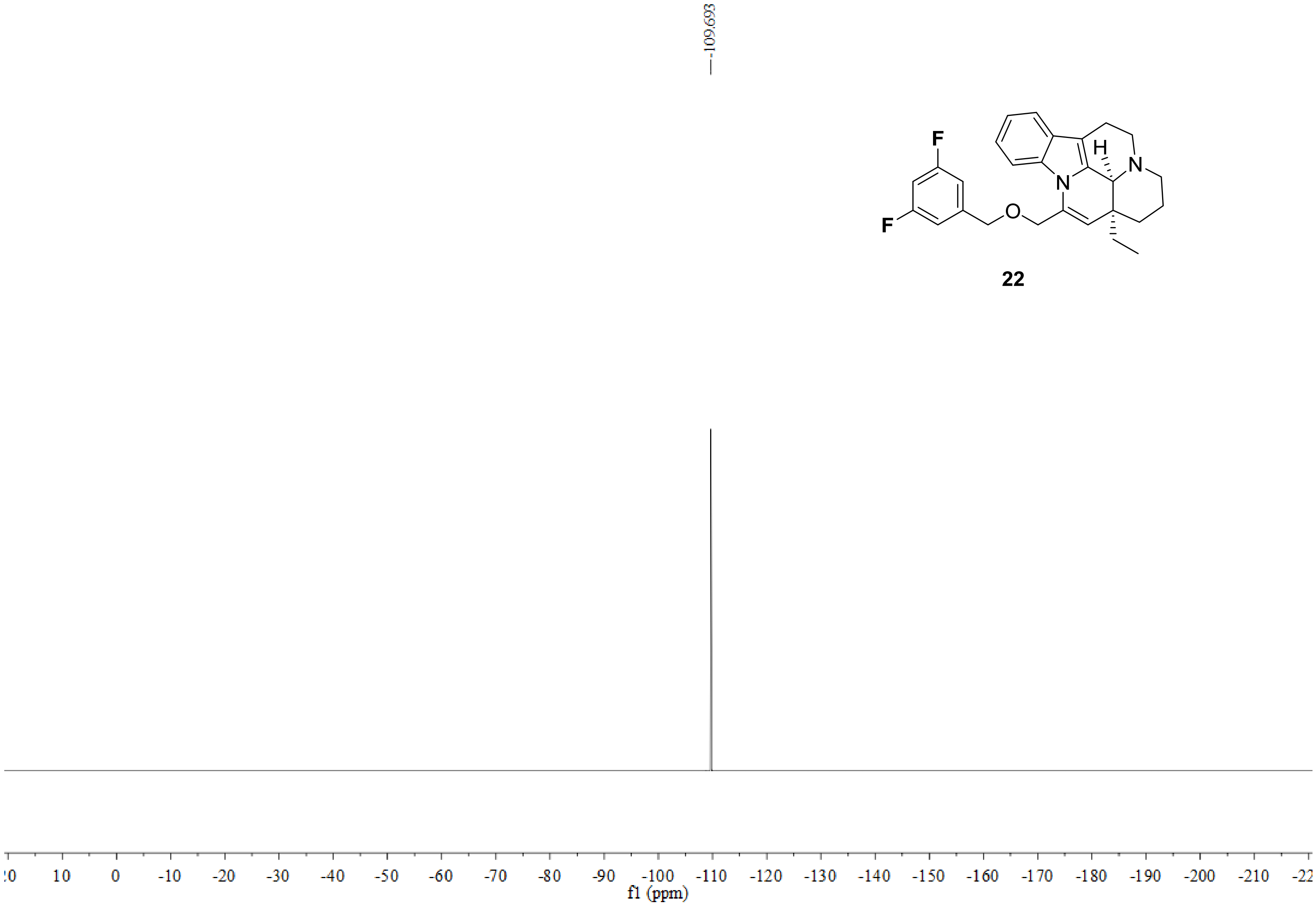

^{19}F NMR of Compound **22** (377 MHz, $CDCl_3$)

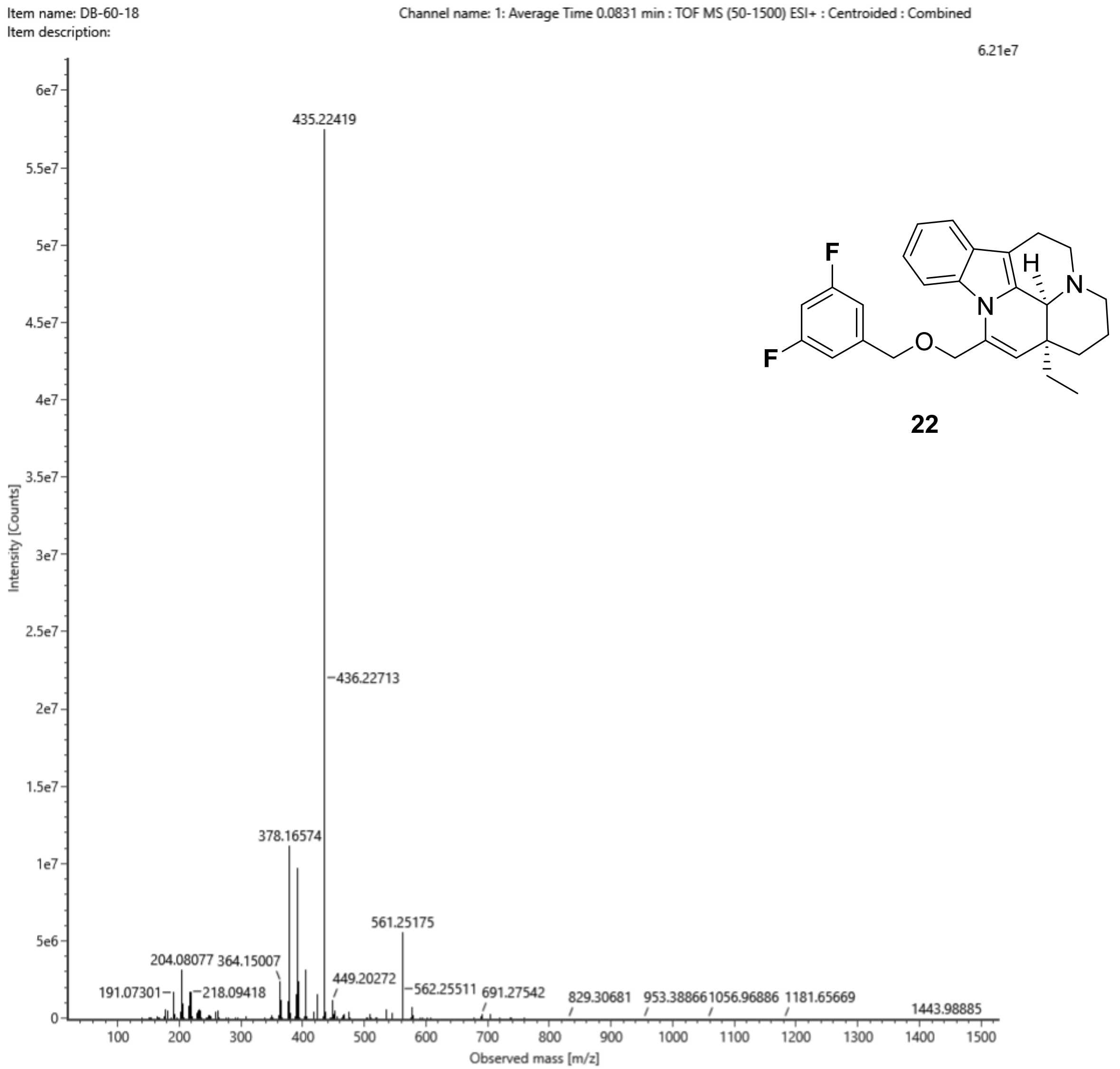

HRMS of Compound **22**

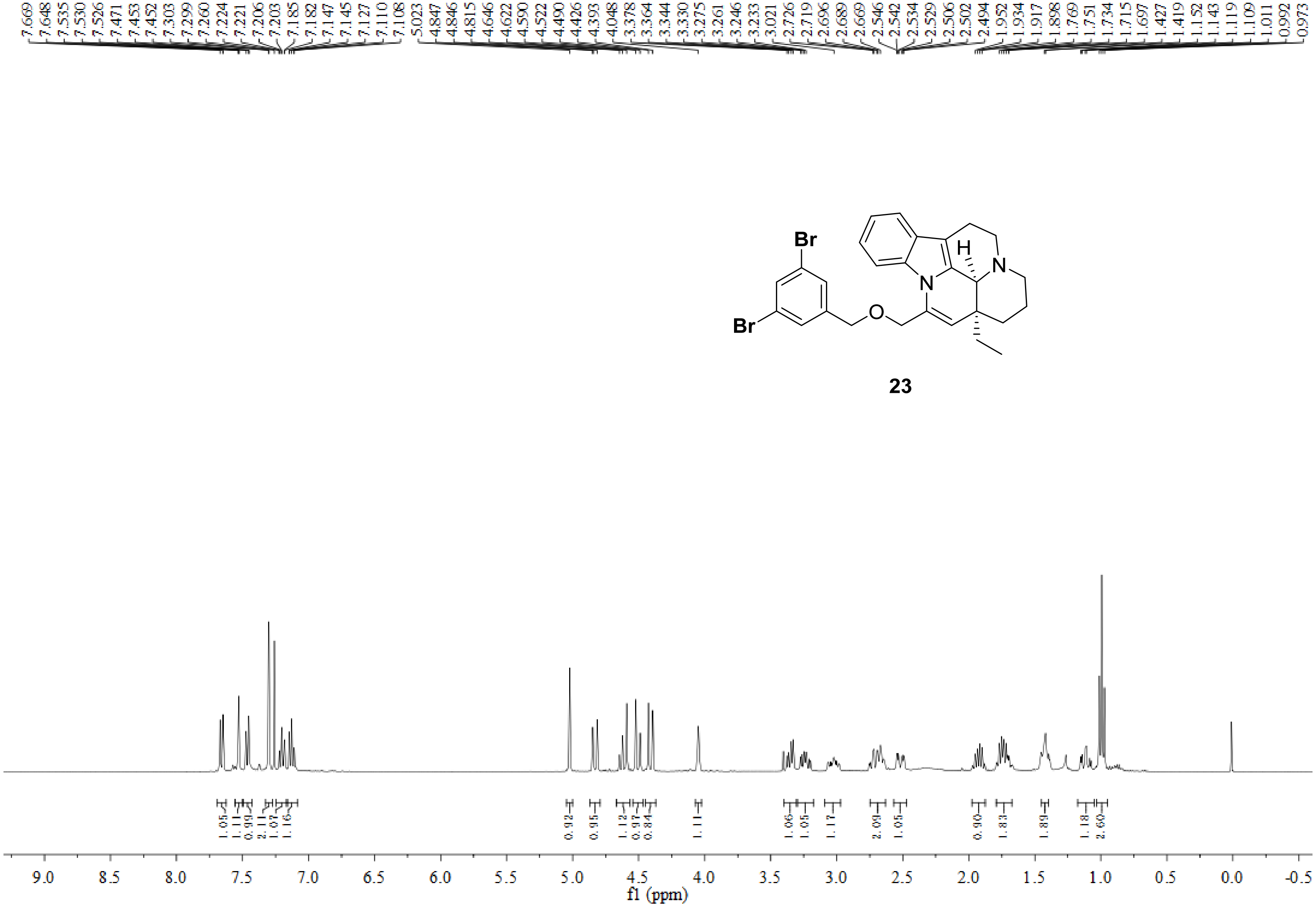

^{1}H NMR of Compound **23** (400 MHz, $CDCl_3$)

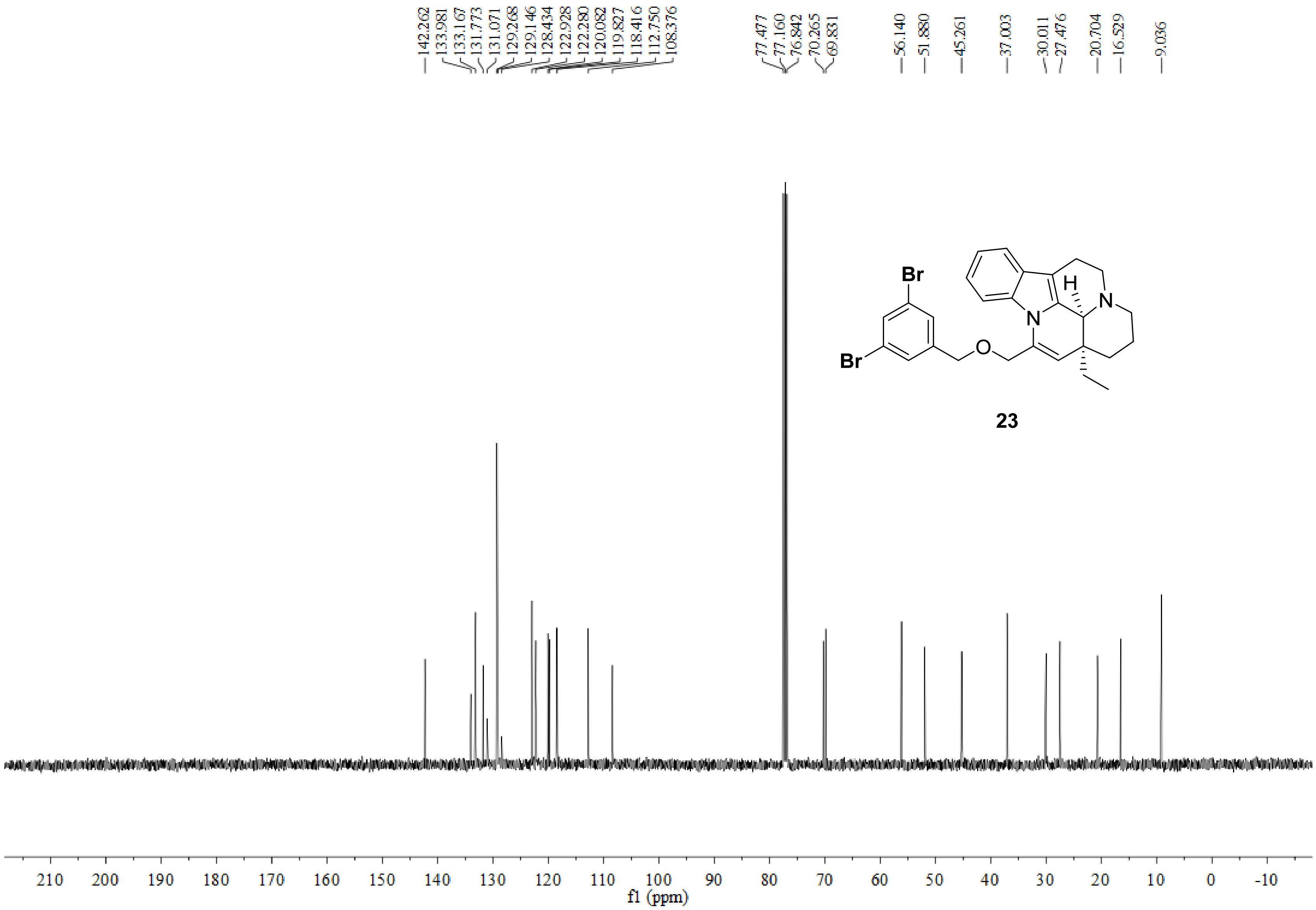

^{13}C NMR of Compound **23** (100 MHz, $CDCl_3$)

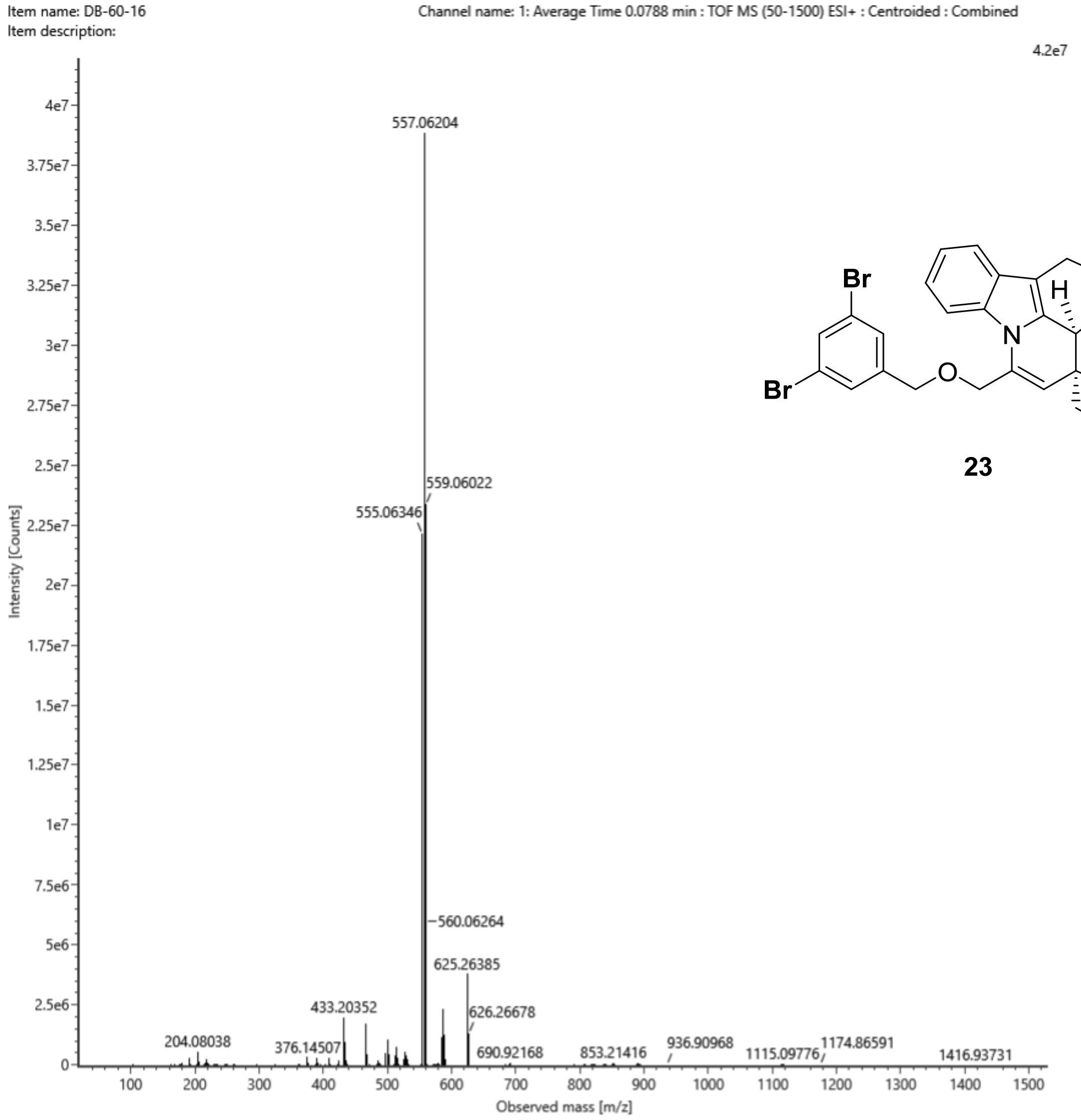

HRMS of Compound **23**

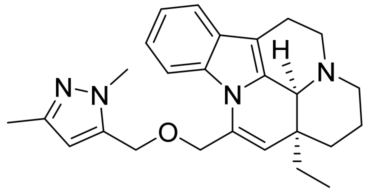

24

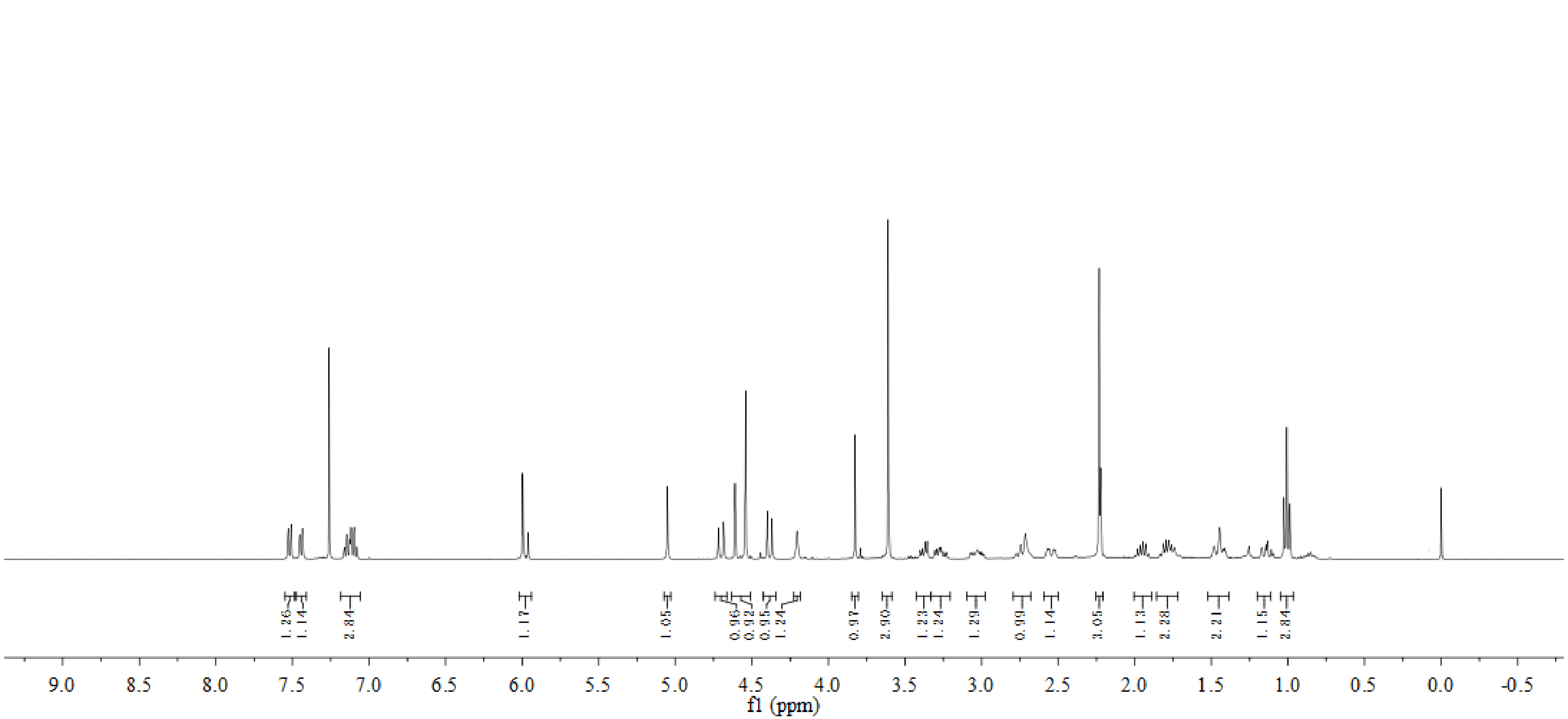

^{1}H NMR of Compound **24** (400 MHz, $CDCl_3$)

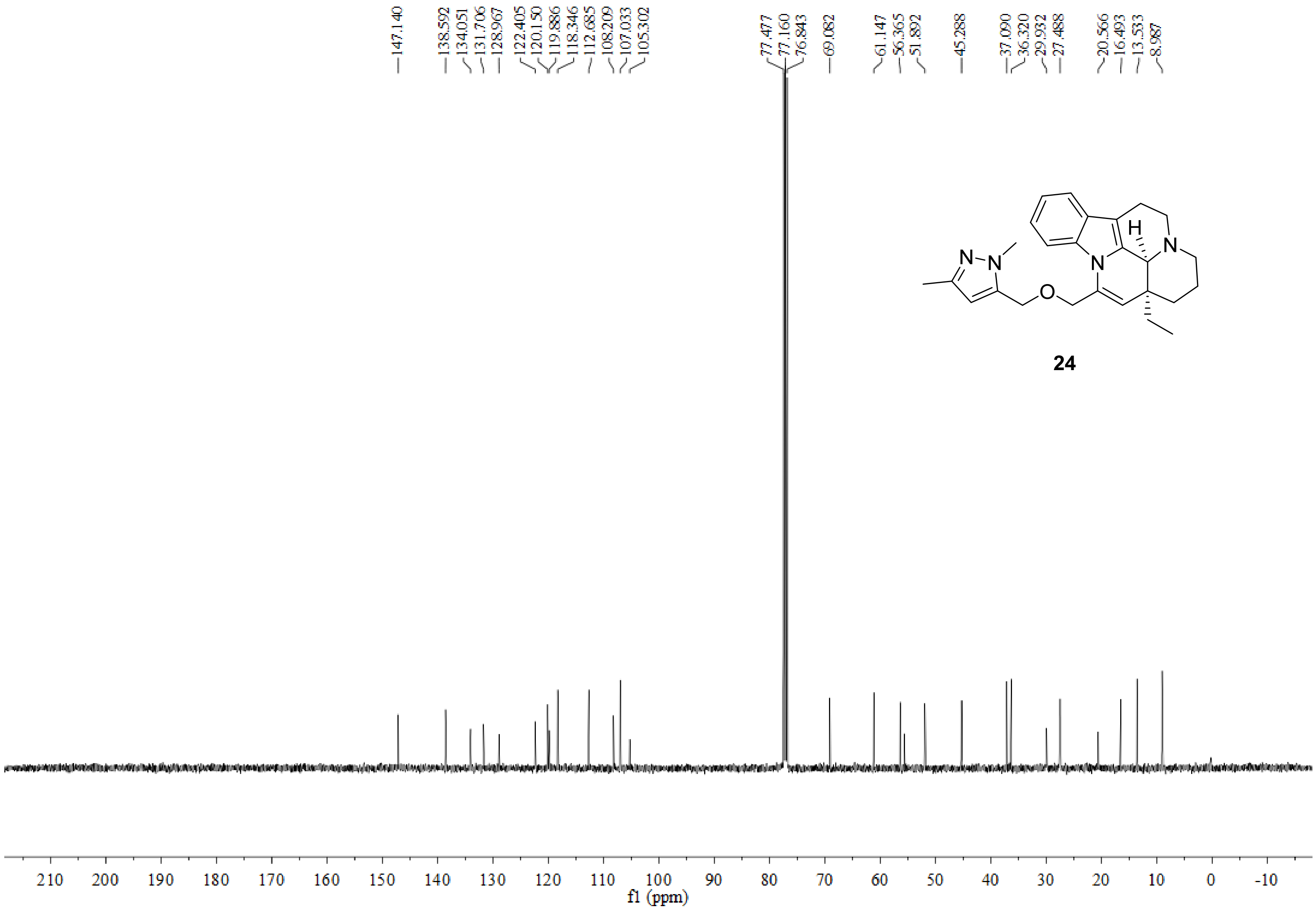

^{13}C NMR of Compound **24** (100 MHz, $CDCl_3$)

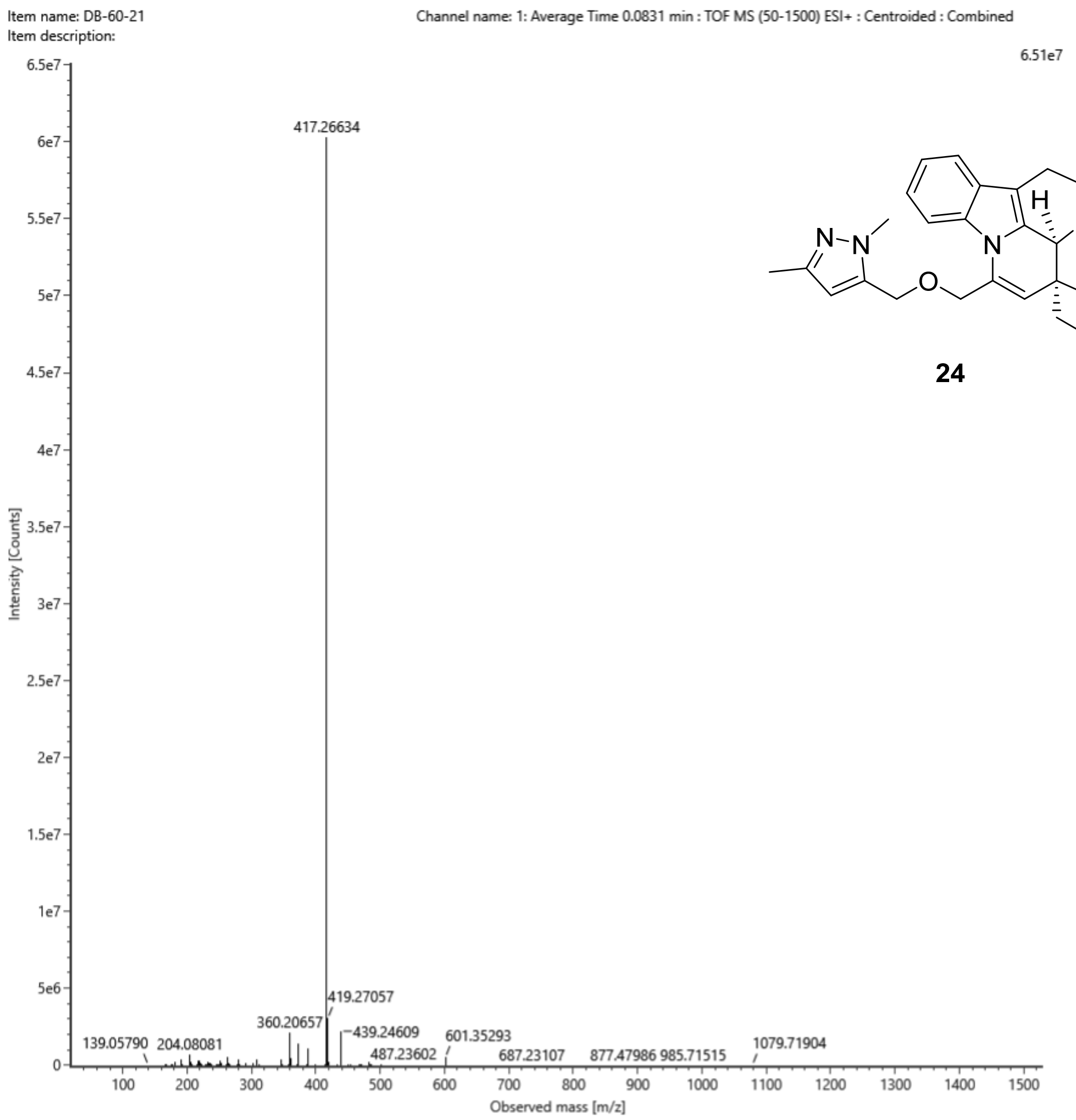

HRMS of Compound **24**

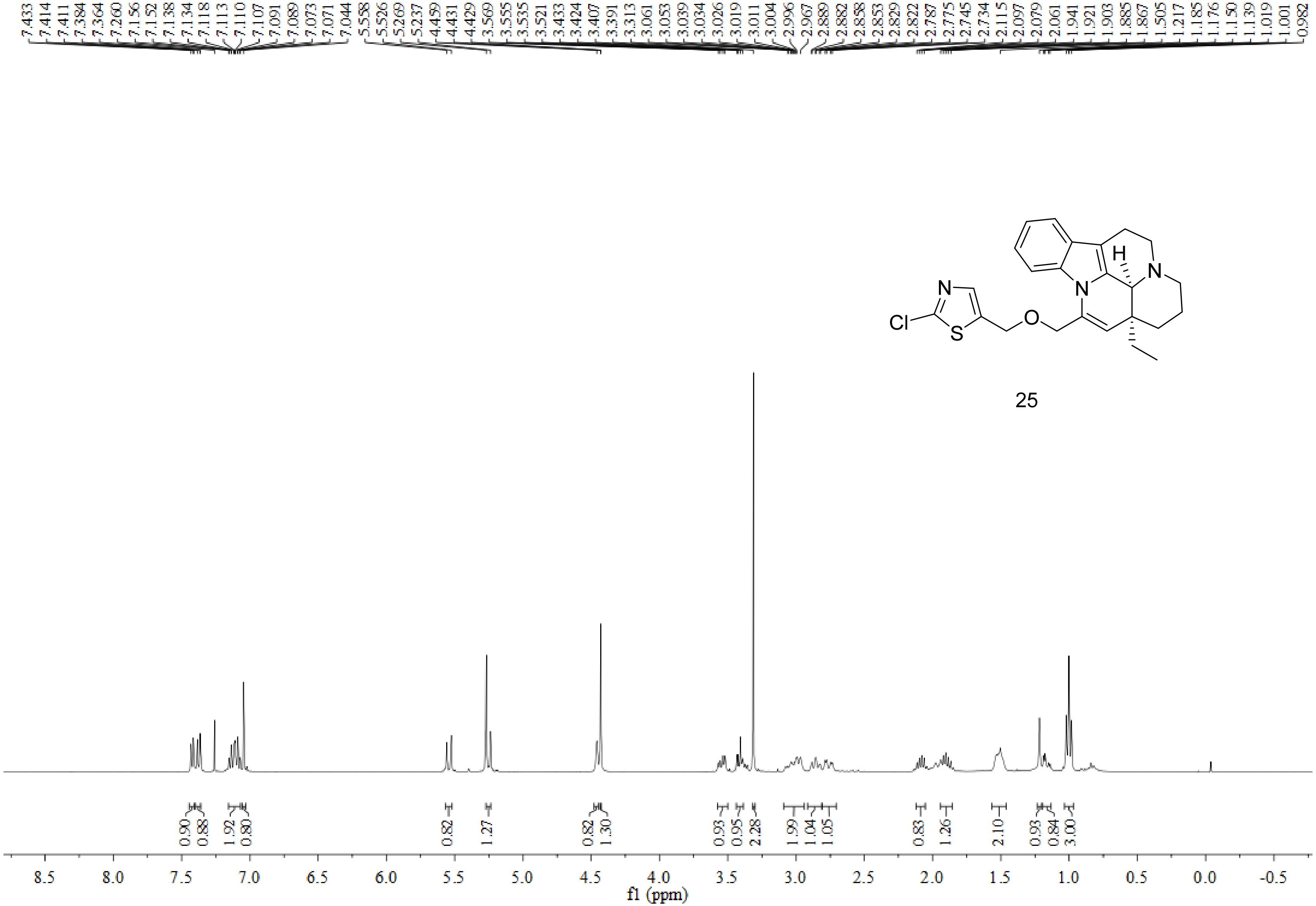

^{1}H NMR of Compound **25** (400 MHz, $CDCl_3$)

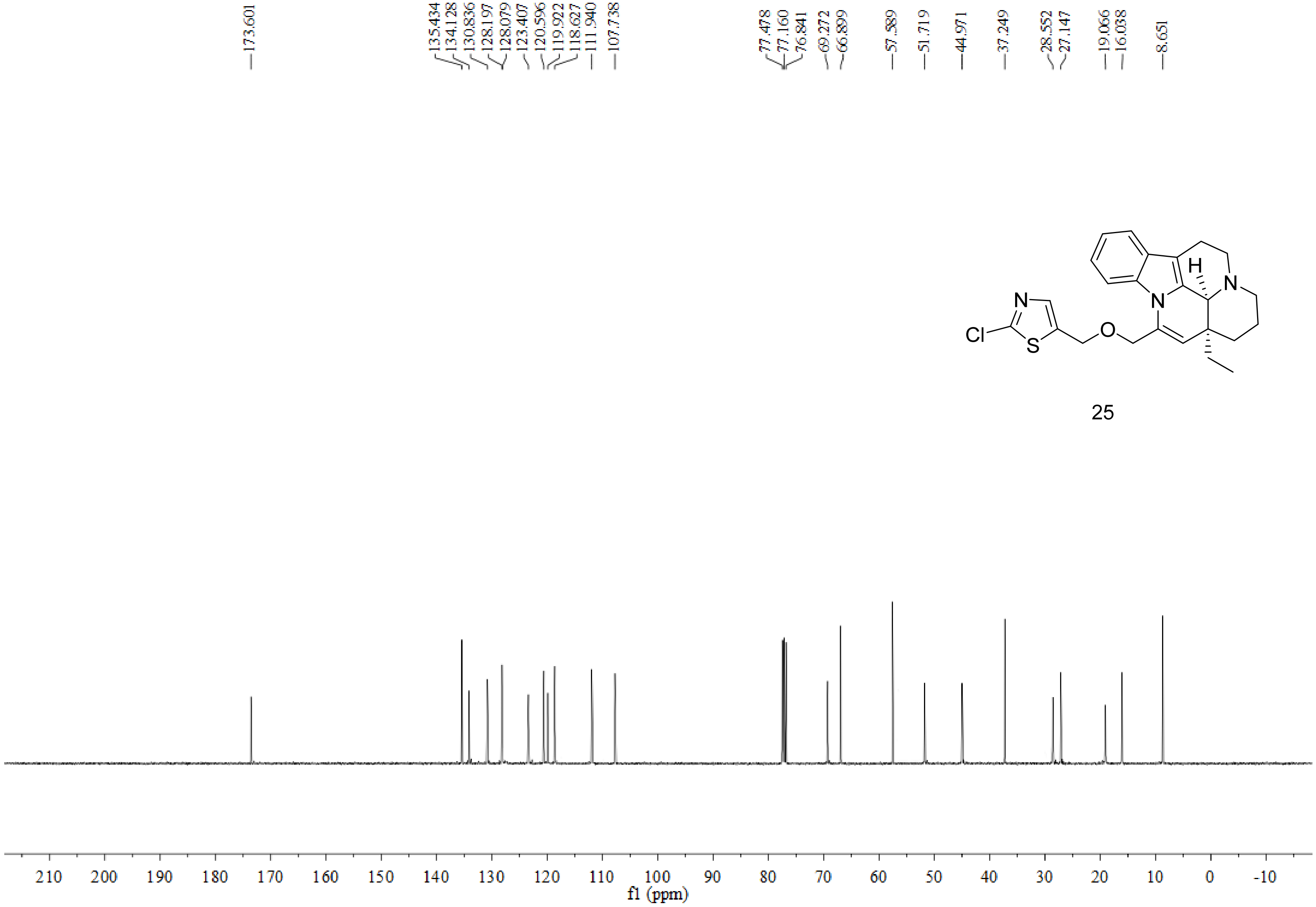

^{13}C NMR of Compound **25** (100 MHz, $CDCl_3$)

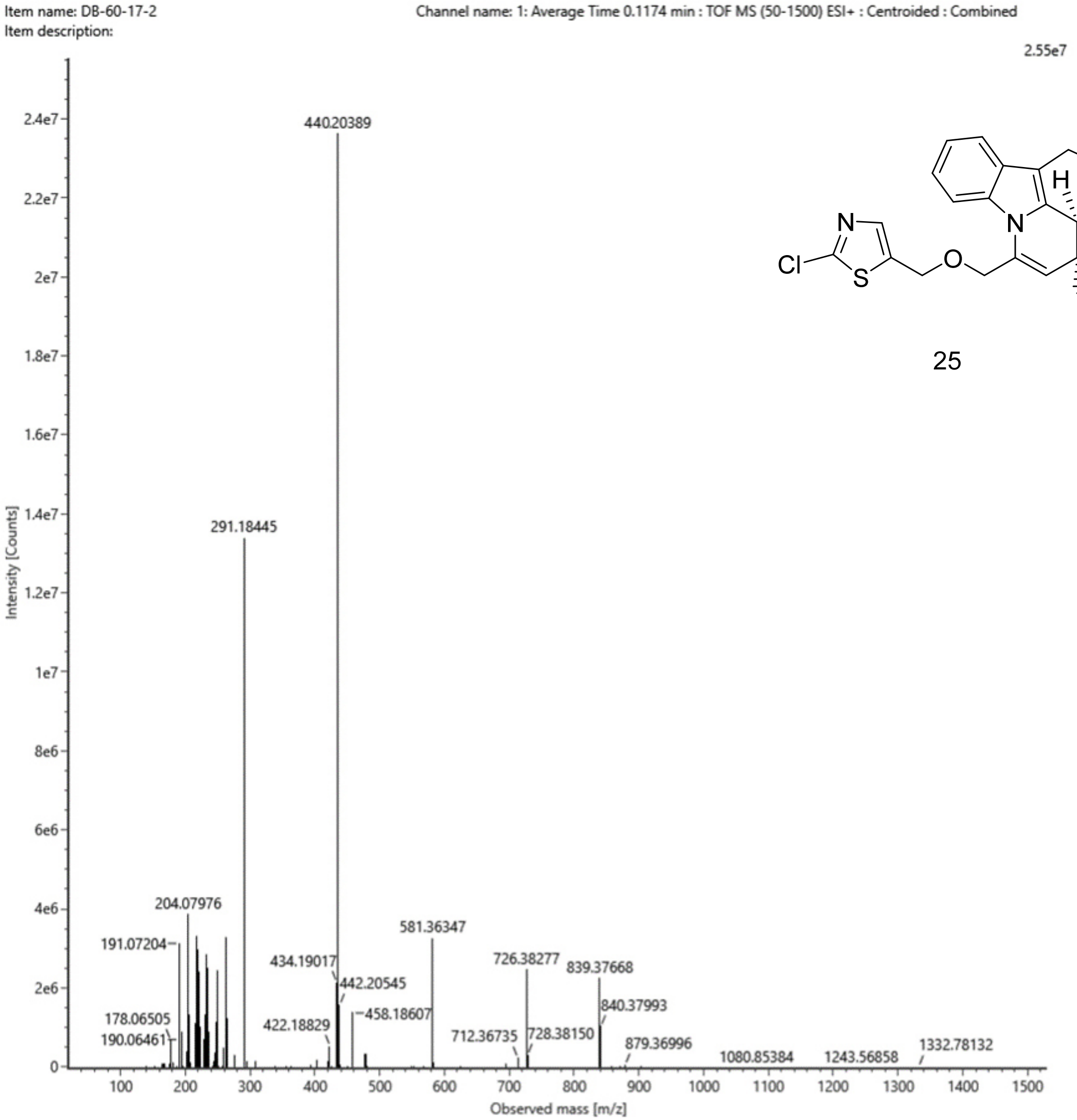

HRMS of Compound **25**
